# Pathway analysis of gene signatures predicting metastasis of node-negative primary breast cancer

**DOI:** 10.1186/1471-2407-7-182

**Published:** 2007-09-25

**Authors:** Jack X Yu, Anieta M Sieuwerts, Yi Zhang, John WM Martens, Marcel Smid, Jan GM Klijn, Yixin Wang, John A Foekens

**Affiliations:** 1 Veridex LLC, a Johnson & Johnson Company, 3210 Merryfield Row, San Diego, CA 92121, USA; 2 Erasmus MC, Josephine Nefkens Institute, Dr. Molewaterplein 50, 3015 GE Rotterdam, The Netherlands; 3 Veridex LLC, a Johnson & Johnson Company, 33 Technology Drive, Warren, NJ 07059, USA

## Abstract

**Background:**

Published prognostic gene signatures in breast cancer have few genes in common. Here we provide a rationale for this observation by studying the prognostic power and the underlying biological pathways of different gene signatures.

**Methods:**

Gene signatures to predict the development of metastases in estrogen receptor-positive and estrogen receptor-negative tumors were identified using 500 re-sampled training sets and mapping to Gene Ontology Biological Process to identify over-represented pathways. The Global Test program confirmed that gene expression profilings in the common pathways were associated with the metastasis of the patients.

**Results:**

The apoptotic pathway and cell division, or cell growth regulation and G-protein coupled receptor signal transduction, were most significantly associated with the metastatic capability of estrogen receptor-positive or estrogen-negative tumors, respectively. A gene signature derived of the common pathways predicted metastasis in an independent cohort. Mapping of the pathways represented by different published prognostic signatures showed that they share 53% of the identified pathways.

**Conclusion:**

We show that divergent gene sets classifying patients for the same clinical endpoint represent similar biological processes and that pathway-derived signatures can be used to predict prognosis. Furthermore, our study reveals that the underlying biology related to aggressiveness of estrogen receptor subgroups of breast cancer is quite different.

## Background

Microarray technology has become a popular tool to classify breast cancer patients into histological subtypes, subgroups with a different prognosis, different site of relapse, and different types of response to treatment [1-9]. A major challenge for application of gene expression profiling is stability of the gene list as a signature [10]. Considering that many genes have correlated expression on a gene expression array, especially for genes involved in the same biological process, it is quite possible that different genes may be present in different signatures when different training sets of patients and different statistical tools are used. Furthermore, genes are usually included in a classifier applying stringent statistical criteria. At these strict significance levels, there is only a small chance for any specific gene to be included. Reproducibility in gene signatures identified in different datasets is thus unlikely [11]. To our knowledge, so far prognostic gene signatures were identified based on the performance of individual genes, regardless of their biological functions. We and others have previously suggested that it might be more appropriate to interrogate the gene lists for biological themes, rather than individual genes [8,12-19]. Moreover, identification of the distinct biological processes between subtypes of cancer patients is more relevant to understand the mechanism of the disease development and for targeted drug development.

In this study we associated biological processes with the tumor's metastatic capability. We re-sampled our data set numerous times to get multiple gene lists whose expression correlated with patients' survival. Based on these gene lists, over-represented pathways defined in Gene Ontology Biological Process (GOBP) were identified for estrogen receptor (ER)-positive or ER-negative breast cancer patients, separately. One step further, we compared the pathways represented by different published prognostic gene signatures with the over-represented pathways associated with metastatic capability. This study also demonstrated it is feasible to construct a gene signature from the key pathways to predict clinical outcomes.

## Methods

### Patient population

The study was approved by the Medical Ethics Committee of the Erasmus MC Rotterdam, The Netherlands (MEC 02.953), and was performed in accordance to the Code of Conduct of the Federation of Medical Scientific Societies in the Netherlands [20]. A cohort of 344 breast tumor samples from our tumor bank at the Erasmus Medical Center (Rotterdam, Netherlands) was used in this study. All these samples were from patients with lymph node-negative breast cancer who had not received any adjuvant systemic therapy, and had more than 70% tumor content. Among them, 286 samples had been used to derive a 76-gene signature to predict distant metastasis [8]. Fifty-eight additional ER-negative cases were included to increase the numbers in this subgroup. According to our previous study [21], array-measured ER status and clinical ER status have the best correlation when the cutoff is set at 1000, after scaling the average intensity of probe sets on an Affymetrix HG-U133A chip to 600. Using array-based ER status allows us to avoid the variations of the measures of ER by either immunohistochemistry or biochemical assays, as well as including tumors whose ER status is undetermined. Therefore, ER status for a patient was determined based on the expression level of the ER gene on the chip in this study. A sample is considered ER-positive if its ER expression level is higher than 1000. Otherwise, the sample is ER-negative [21]. As a result, there are 221 ER-positive and 123 ER-negative patients in the 344-patient population. The mean age of the patients was 53 years (median 52, range 26–83 years), 197 (57%) were premenopausal and 147 (43%) postmenopausal. T1 tumors (≤ 2 cm) were present in 168 patients (49%), T2 tumors (> 2–5 cm) in 163 patients (47%), T3/4 tumors (> 5 cm) in 12 patients (3%), and 1 patient had unknown tumor stage. Pathological examination was carried out by regional pathologists as described previously [22] and the histological grade was coded as poor in 184 patients (54%), moderate in 45 patients (13%), good in 7 patients (2%), and unknown for 108 patients (31%). During follow-up 103 patients showed a relapse within 5 years and were counted as failures in the analysis for DMFS. Eighty two patients died after a previous relapse. The median follow-up time of patients still alive was 101 months (range 61–171 months).

### RNA isolation and hybridization

Total RNA was extracted from 20–40 cryostat sections of 30 um thickness with RNAzol B (Campro Scientific, Veenendaal, Netherlands). After being biotinylated, targets were hybridized to Affymetrix HG-U133A chips as described [8]. Gene expression signals were calculated using Affymetrix GeneChip analysis software MAS 5.0. Chips with an average intensity less than 40 or a background higher than 100 were removed. Global scaling was performed to bring the average signal intensity of a chip to a target of 600 before data analysis. For the validation dataset [23], quantile normalization was performed and ANOVA was used to eliminate batch effects from different sample preparation methods, RNA extraction methods, different hybridization protocols and scanners.

### Multiple gene signatures

**Figure 1:**
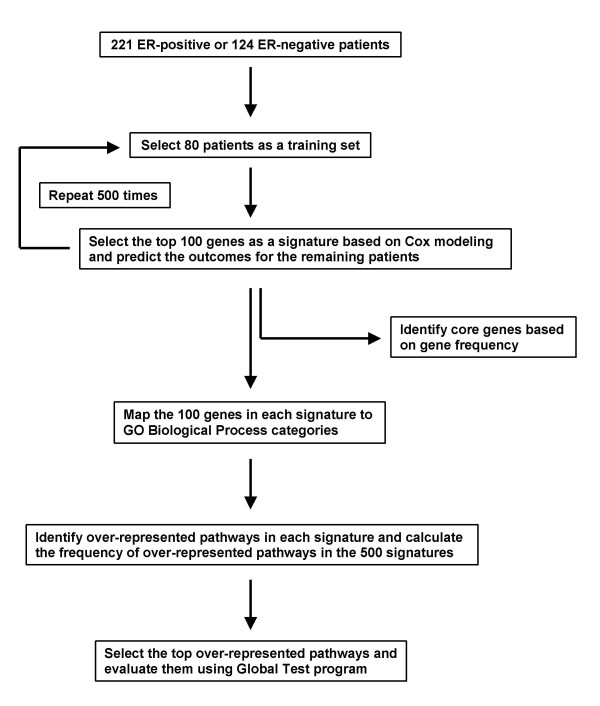
Work flow of data analysis for deriving core genes and over-represented pathways.

For ER-positive and ER-negative patients, 80 samples were randomly selected as a training set and univariate Cox proportional-hazards regression was performed to identify genes whose expression patterns were most correlated to patients' DMFS time. Our previous analysis suggested that 80 patients represent a minimum size of the training set for producing a prognostic gene signature with stable performance [8]. Because the majority of the published gene expression signatures had less than 100 genes, the top 100 genes from the Cox regression were used as a signature to predict tumor recurrence for the remaining patients. A relapse score for a patient was used to calculate a patient's risk of distant metastasis and was defined as the linear combination of logarithmically transformed gene expression levels weighted by the standardized Cox regression coefficient as described [8]. ROC analysis with distant metastasis within 5 years as a defining point was conducted. Patients who did not have 5-year follow-up were excluded from ROC analysis. The AUC of the ROC plots was used as a measure of the performance of a signature in the test set. The above procedure was repeated 500 times (Figure 1). Thus, 500 signatures of 100 genes each were obtained for both the ER-positive and ER-negative subgroups. The frequency of the selected genes in the 500 signatures was calculated and the genes were ranked based on the frequency.

As a control, the patient survival data for the ER-positive patients or ER-negative patients was permuted randomly and re-assigned to the chip data. As described above, 80 chips were then randomly selected as a training set and the top 100 genes were selected using the Cox modeling based on the permuted clinical information. The clinical information was permuted 10 times. For each permutation of the survival data, 50 training sets of 80 patients were created. For each training set, the top 100 genes were obtained as a control gene list based on the Cox modeling. Thus, a total of 500 control signatures were obtained. The predictive performance of the 100 genes was examined in the remaining patients. A ROC analysis was conducted and AUC was calculated in the test set.

### Mapping signatures to GOBP and identification of over-represented pathways

To identify over-representation of biological pathways in the signatures, genes on Affymetrix HG-U133A chip were mapped to the categories of GOBP based on the annotation table downloaded from [24]. Categories that contained at least 10 probe sets from the HG-U133A chip were retained for subsequent pathway analysis. As a result, 304 categories were used for following pathway analysis. The 100 genes of each signature were mapped to GOBP. Hypergeometric distribution probabilities for all included GOBP categories were calculated for each signature to evaluate its statistical significance. A pathway that had a hypergeometric distribution probability < 0.05 and was hit by two or more genes from the 100 genes was considered an over-represented pathway in a signature. The total number of times a pathway occurred in the 500 signatures was considered as the frequency of over-representation.

To evaluate the relationship between a pathway as a whole and the clinical outcome, each of the top 20 over-represented pathways that have the highest frequencies in the 500 signatures were subjected to Global Test program [12,14]. The Global Test examines the association of a group of genes as a whole to a specific clinical parameter such as DMFS. The contribution of individual genes in the top over-represented pathways to the association was also evaluated.

### Building pathway-based signatures

To explore the possibility of using the genes from over-represented pathways as a signature to predict distant metastasis, the top two pathways for ER-positive and ER-negative tumors that were in the top 20 list based on frequency of over-representation and had the smallest p values with the Global Test program were chosen to build a gene signature. First, genes in the pathway were selected if their z-score was greater than 1.96 from the Global Test program. A z-score greater than 1.96 indicates that the association of the gene expression with DMFS time is significant (p < 0.05) [12,14]. To determine the optimal number of genes in a given pathway used for building the signature, combinations of gene markers were tested by adding one gene at a time according to their z-scores. The number of significant genes that gave the highest AUC value of the ROC analysis with distant metastasis within 5 years as the defining point was considered optimal and used to build a pathway-based signature.

The relapse score for a given patient was calculated as the difference between the linear combination of the logarithmically transformed expression signals weighted by their z-scores for negatively correlated genes and that for positively correlated genes. The predicting performance of the gene signature was evaluated by ROC and Kaplan-Meier survival analysis in an independent patient group [23] for ER-positive patients and ER-negative patients both separately and combined.

### Comparing multiple gene signatures

To compare the genes from various prognostic signatures for breast cancer, five gene signatures were selected [3,8,23,25,26]. Identity of the genes between the signatures was determined by BLAST program. To examine the representation of the top 20 pathways in the signatures, genes in each of the signatures were mapped to GOBP.

### Data availability

The microarray data analyzed in this paper have been submitted to the NCBI/Genbank GEO database (series entry GSE2034 for the first 286 patients, and GSE5327 for the additional 58 patients). The microarray and clinical data used for the independent validation testing set analysis were obtained from the GEO database with accession number GSE2990.

## Results

### Multiple gene signatures

**Figure 2:**
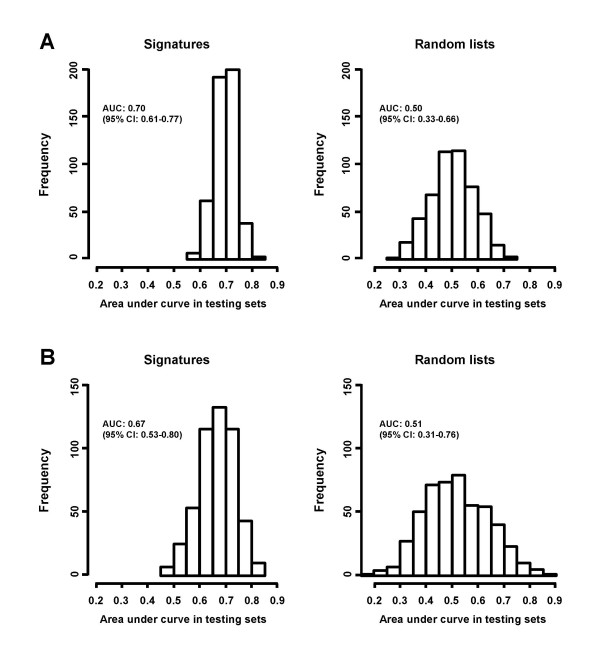
**Evaluation of the 500 gene signatures**. Each of the 100-gene signatures for 80 randomly selected tumors in the training set was used to predict relapsed patients in the corresponding test set. Its performance was measured by the AUC of the ROC analysis. (**A**) Performance of the gene signatures for ER-positive patients in test sets. (**B**) Performance of the gene signatures for ER-negative patients in test sets. (Left) Frequency of AUC in 500 prognostic signatures panels as derived following the flow chart presented in Figure 1. (Right) Frequency of AUC in 500 random gene lists. To generate a gene list as a control, the survival data for the ER-positive patients or ER-negative patients was permutated randomly and reassigned to the chip data.

Using re-sampling, we constructed a total of 1,000 prognostic gene signatures derived from different patient groups aiming to improve understanding of the underlying biological processes of breast cancer metastasis. Since gene expression patterns of ER-subgroups of breast tumors are quite different [1-4,8,27] data analysis to derive gene signatures and subsequent pathway analysis were conducted separately [8]. For both ER-positive and ER-negative patients, 80 samples were randomly selected as a training set and the 100 genes most significantly associated with distant metastasis-free survival (DMFS) were used as a signature to predict tumor recurrence for the remaining ER-positive and ER-negative patients, respectively (Figure 1). The area under the curve (AUC) of receiver operating characteristic (ROC) analysis with distant metastasis within 5 years as a defining point was used as a measure of the performance of a signature in a corresponding test set. The above procedure was repeated 500 times. The average of AUCs for the 500 signatures in the ER-positive test sets was 0.70 (95% confidence interval (CI): 0.61–0.77) whereas the average of AUCs for 500 random gene lists was 0.50 (95% CI: 0.33–0.66), indicating a non-random prediction for the true test sets (Figure 2A). For ER-negative datasets, these values of average AUCs were 0.67 (95% CI: 0.53–0.80) and 0.51 (95% CI: 0.31–0.76), respectively (Figure 2B). The results demonstrate that depending on the training set different gene signatures can be identified with comparable performance. This could explain the results obtained by earlier studies, which reported different gene signatures with similar power to predict risk groups. The 20 most frequently found genes in the 500 signatures for ER-positive and ER-negative tumors are listed in Table 1. The most frequent genes were KIAA0241 protein *(KIAA0241) *for ER-positive tumors, and zinc finger protein multitype 2 *(ZFPM2) *for ER-negative tumors. There was no overlap between genes of the ER-positive and -negative core gene lists suggesting that different molecular mechanisms are associated with the subtypes of breast cancer disease.

**Table 1:** Genes with highest frequencies in 500 signatures

Gene title	Gene symbol	Frequency
Top 20 core genes from ER-positive tumors		
KIAA0241 protein	KIAA0241	321
CD44 antigen (homing function and Indian blood group system)	CD44	286
ATP-binding cassette, sub-family C (CFTR/MRP), member 5	ABCC5	251
serine/threonine kinase 6	STK6	245
cytochrome c, somatic	CYCS	235
KIAA0406 gene product	KIA0406	212
uridine-cytidine kinase 1-like 1	UCKL1	201
zinc finger, CCHC domain containing 8	ZCCHC8	188
Rac GTPase activating protein 1	RACGAP1	186
staufen, RNA binding protein (Drosophila)	STAU	176
lactamase, beta 2	LACTB2	175
eukaryotic translation elongation factor 1 alpha 2	EEF1A2	172
RAE1 RNA export 1 homolog (S. pombe)	RAE1	153
tuftelin 1	TUFT1	150
zinc finger protein 36, C3H type-like 2	ZFP36L2	150
origin recognition complex, subunit 6 homolog-like (yeast)	ORC6L	143
zinc finger protein 623	ZNF623	140
extra spindle poles like 1	ESPL1	139
transcription elongation factor B (SIII), polypeptide 1	TCEB1	138
ribosomal protein S6 kinase, 70 kDa, polypeptide 1	RPS6KB1	127
		
Top 20 core genes from ER-negative tumors		
zinc finger protein, multitype 2	ZFPM2	445
ribosomal protein L26-like 1	RPL26L1	372
hypothetical protein FLJ14346	FLJ14346	372
mitogen-activated protein kinase-activated protein kinase 2	MAPKAPK2	347
collagen, type II, alpha 1	COL2A1	340
muscleblind-like 2 (Drosophila)	MBNL2	320
G protein-coupled receptor 124	GPR124	314
splicing factor, arginine/serine-rich 11	SFRS11	300
heterogeneous nuclear ribonucleoprotein A1	HNRPA1	297
CDC42 binding protein kinase alpha (DMPK-like)	CDC42BPA	296
regulator of G-protein signalling 4	RGS4	276
transient receptor potential cation channel, subfamily C, member 1	TRPC1	265
transcription factor 8 (represses interleukin 2 expression)	TCF8	263
chromosome 6 open reading frame 210	C6orf210	262
dynamin 3	DNM3	260
centrosome protein Cep63	Cep63	251
tumor necrosis factor (ligand) superfamily, member 13	TNFSF13	251
dapper, antagonist of beta-catenin, homolog 1 (Xenopus laevis)	DACT1	248
heterogeneous nuclear ribonucleoprotein A1	HNRPA1	245
reversion-inducing-cysteine-rich protein with kazal motifs	RECK	243

The top 20 genes are ranked by their frequency in the 500 signatures of 100 genes for ER-positive and ER-negative tumors (for details see Figure 1).

### Over-represented pathways in gene signatures and Global Test

The 100 genes in each of the 500 signatures for ER-positive and ER-negative tumors were mapped to the categories of GOBP. For a given gene signature, a pathway (or category) that had a hypergeometric distribution probability smaller than 0.05 and included two or more genes was considered an over-represented pathway. The "inclusion of 2 or more genes" as a selection criterion in addition to the statistical significance was to avoid selecting statistically significant pathways containing only one gene in the signature. The frequency of over-representation of GOBP in the 500 signatures for ER-positive and ER-negative dataset was calculated. Like the observation of most frequently found genes, the biological pathways over-represented in the gene signatures are distinct for ER-positive and ER-negative tumors (Table 2). For ER-positive tumors, cell division-related processes and immune-response-related pathways are frequently found in the top 20 over-represented pathways. All of the 20 pathways had a significant association with DMFS as analyzed by the Global Test program [12,14], with the 2 most significant being "apoptosis" (mainly containing genes of the extrinsic apoptotic pathway) and "regulation of cell cycle" (Table 2). For ER-negative tumors, many of the top 20 pathways are related with RNA processing, transportation and signal transduction. Eighteen of the top 20 pathways demonstrated a significant association with DMFS in the Global Test, the 2 most significant being "regulation of cell growth" and "regulation of G-protein coupled receptor signaling" (Table 2).

**Table 2:** Top 20 pathways in the 500 signatures of ER-positive and ER-negative tumors evaluated by Global Test

Pathways	GO_ID	P	Frequency
ER-positive tumors			
Apoptosis	6915	3.06E-7	250
Regulation of cell cycle	74	2.46E-5	203
Protein amino acid phosphorylation	6468	2.48E-5	114
Cytokinesis	910	6.13E-5	165
Cell motility	6928	0.00015	93
Cell cycle	7049	0.00028	138
Cell surface receptor-linked signal transd.	7166	0.00033	172
Mitosis	7067	0.00036	256
Intracellular protein transport	6886	0.00054	141
Mitotic chromosome segregation	70	0.00057	98
Ubiquitin-dependent protein catabolism	6511	0.00074	158
DNA repair	6281	0.00079	156
Induction of apoptosis	6917	0.00083	115
Immune response	6955	0.00094	167
Protein biosynthesis	6412	0.0010	145
DNA replication	6260	0.0015	92
Oncogenesis	7048	0.0020	228
Metabolism	8152	0.0021	83
Cellular defense response	6968	0.0025	131
Chemotaxis	6935	0.0027	89
			
ER-negative tumors			
Regulation of cell growth	1558	0.00012	136
Regul. of G-coupled receptor signaling	8277	0.00013	153
Skeletal development	1501	0.00024	160
Protein amino acid phosphorylation	6468	0.0051	151
Cell adhesion	7155	0.0065	110
Carbohydrate metabolism	5975	0.0066	86
Nuclear mRNA splicing, via spliceosome	398	0.0067	203
Signal transduction	7165	0.0078	160
Cation transport	6812	0.0098	160
Calciumion transport	6816	0.010	93
Protein modification	6464	0.011	132
Intracellular signaling cascade	7242	0.012	135
mRNA processing	6397	0.012	81
RNA splicing	8380	0.014	192
Endocytosis	6897	0.026	166
Regul. of transcription from PolII promoter	6357	0.031	109
Regulation of cell cycle	74	0.043	88
Protein complex assembly	6461	0.048	183
Protein biosynthesis	6412	0.063	99
Cell cycle	7049	0.084	72

Each of the top 20 over-represented pathways that have the highest frequencies in the 500 signatures of ER-positive and ER-negative tumors were subjected to Global Test program [12, 14]. The Global Test examines the association of a group of genes as a whole to a specific clinical parameter, in this case DMFS, and generates an asymptotic theory p value for the pathway. The pathways are ranked by their p value in the respective ER-subgroup of tumors.

**Figure 3:**
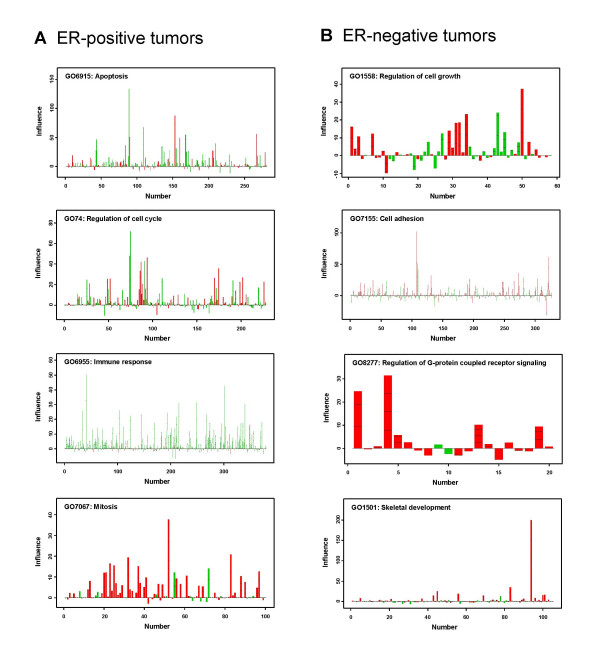
**Association of the expression of individual genes with DMFS time for selected over-represented pathways**. The Geneplot function in the Global Test program [12, 14] was applied and the contribution of the individual genes in each selected pathway is plotted. The numbers at the X-axis represent the number of genes in the respective pathway in ER-positive (Left) or ER-negative tumors (Right). The values at the Y-axis, represent the contribution (influence) of each individual gene in the selected pathway with DMFS. Negative values indicate there is no association between the gene expression and DMFS. Horizontal markers in a bar indicates one standard deviation away from the reference point, two or more horizontal markers in a bar indicates that the association of the corresponding gene with DMFS is statistically significant. The green bars reflect genes that are positively associated with DMFS, indicating a higher expression in tumors without metastatic capability. The red bars reflect genes that are negatively associated with DMFS, indicative of higher expression in tumors with metastatic capability. (**A**) ER-positive tumors: from top to bottom: "apoptosis" pathway consisting of 282 genes, "regulation of cell cycle" pathway consisting of 228 genes, "immune response" pathway consisting of 379 genes, and "mitosis"? pathway consisting of 100 genes. (**B**) ER-negative tumors: from top to bottom: "regulation of cell growth" pathway consisting of 58 genes, "cell adhesion" pathway consisting of 327 genes, "regulation of G-coupled receptor signaling" pathway consisting of 20 genes, and "skeletal development" pathway consisting of 105 genes.

The contribution and significance of individual genes in the top over-represented pathways to the association with DMFS were determined for ER-positive [see Additional files 1 and 2] and ER-negative tumors [see Additional files 3 and 4]. Genes can either show a positive association with DMFS, indicating a higher expression in tumors without metastatic capability, or a negative association, indicative of a higher expression in metastatic tumors. In ER-positive tumors, pathways with a mixed association include the 2 most significant pathways "apoptosis" and "regulation of cell cycle" (Figure 3A). There were also a number of pathways that had a predominant positive or negative correlation with DMFS. For example, the pathway "immune response" is associated with 379 probe sets, of which the majority showed positive correlation to DMFS (Figure 3A). Similarly in the biological processes "cellular defense response" and "chemotaxis", most genes displayed a strong positive correlation with DMFS [see Additional file 1]. On the other hand, genes in "mitosis" (Figure 3A), "mitotic chromosome segregation" and "cell cycle" showed a predominant negative correlation with DMFS [see Additional file 1].

In ER-negative tumors (Figure 3B), examples of pathways with genes that had both positive and negative correlation to DMFS include "regulation of cell growth", the most significant pathway, and "cell adhesion". Of the top 20 pathways in ER-negative tumors, none showed a dominant positive association with DMFS. Although for some pathways most genes correlated negatively with DMFS [see Additional file 3], including "regulation of G-protein coupled receptor signaling" and "skeletal development" (Figure 3B), ranked among the top 3 pathways in significance (Table 2). Of the top 20 core pathways 4 overlapped between ER-positive and -negative tumors, i.e., "regulation of cell cycle", "protein amino acid phosphorylation", "protein biosynthesis", and "cell cycle" (Table 2).

### Pathway-derived gene expression profiles as a predictor

**Figure 4:**
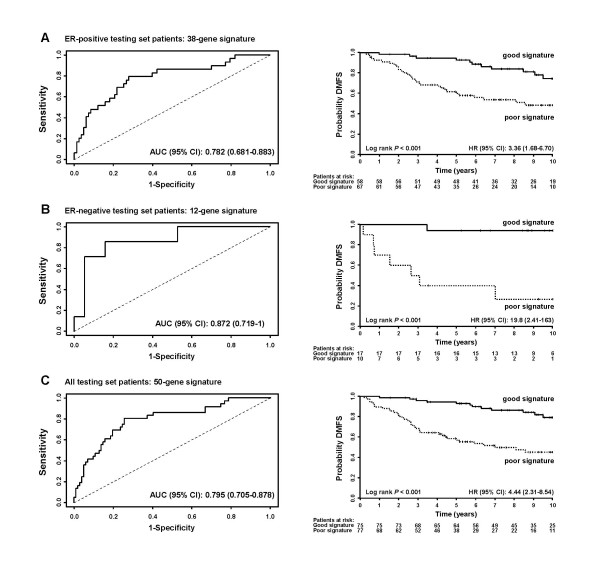
**Validation of pathway-based breast cancer classifiers constructed from the optimal significant genes**. To find the optimal number of genes as a signature, ROC analyses, with 5-year DMFS as defining point, with an increasing number of genes were conducted in the training set of ER-positive tumors or ER-negative tumors. For ER-positive tumors, in the "apoptosis" pathway, 24 genes (reaching an AUC of 0.784) were considered optimal (Table 3). For the "regulation of cell cycle pathway" in ER-positive tumors, 17 genes (AUC of 0.777) were considered optimal (Table 4). For ER-negative tumors, the optimal number of genes was 7 (AUC of 0.790) for the "regulation for cell growth" pathway (Table 5), and 5 (AUC of 0.788) for the "regulation of G-protein coupled receptor signaling" pathway (Table 6), respectively. The selected genes for the top 2 pathways for ER-positive and ER-negative tumors were subsequently used to construct prognostic gene signatures separately for the 2 ER-subgroups of tumors. The 152-patient test set [23] consisted of 125 ER-positive tumors and 27 ER-negative tumors based on the expression level of ER gene on the chip. (**A**) ROC (Left) and Kaplan-Meier (Right) analysis of the 38-gene signature for ER-positive tumors. Thirteen patients with less than 5-year follow-up were excluded from ROC analysis. (**B**) ROC (Left) and Kaplan-Meier (Right) analysis of the 12-gene signature for ER-negative tumors. One patient with less than 5-year follow-up was excluded from ROC analysis. (**C**) ROC (Left) and Kaplan-Meier (Right) analysis of a combined 50-gene signature for ER-positive and ER-negative tumors. Fourteen patients with less than 5-year follow-up were excluded from ROC analysis.

In an attempt to use gene expression profiles in the most significant biological processes to predict distant metastases we used the genes of the top 2 significant pathways in both ER-positive (Table 3, Table 4) and -negative tumors (Table 5, Table 6) to construct a gene signature for the prediction of distant recurrence. A 50-gene signature was constructed by combining the 38 genes from the top 2 ER-positive pathways ("apoptosis", "regulation of cell cycle") and 12 genes for the top 2 ER-negative pathways ("regulation of cell growth", "regulation of G-coupled receptor signaling"). This signature was further validated using an independent 152-patient cohort [23], which consisted of 125 ER-positive tumors and 27 ER-negative tumors (after removing 36 lymph node positive patients and a patient who died 15 days after surgery). When the 38 genes was applied to the 125 ER-positive patients, a ROC analysis gave an AUC of 0.782 (95% CI: 0.681–0.883) (Figure 4A, left), and Kaplan-Meier analysis for DMFS showed a clear separation in risk groups (p < 0.001, HR: 3.36 and 95% CI: 1.68–6.70) (Figure 4A, right). For the 12 genes for the 27 ER-negative patients, an AUC of 0.872 (95% CI: 0.719–1) (Figure 4B, left) and separation between risk groups with a p < 0.001 and a HR of 19.8 (95% CI: 2.41–163) (Figure 4B, right) was obtained. The combined 50-gene signature for ER-positive and ER-negative patients gave an AUC of 0.795 (95% CI: 0.705–0.878) (Figure 4C, left) and a p < 0.001 and a HR of 4.44 (95% CI: 2.31–8.54) for separation between risk groups (Figure 4C, right).

**Table 3:** Significant genes in the Apoptosis pathway in ER-positive tumors

Probe Set	z-score	DMFS	Gene Symbol	Gene Title
208905_at	4.29	-	CYCS	cytochrome c, somatic
204817_at	3.73	-	ESPL1	extra spindle poles like 1
38158_at	3.41	-	ESPL1	extra spindle poles like 1
204947_at	3.04	-	E2F1	E2F transcription factor 1
201111_at	3.04	-	CSE1L	CSE1 chromosome segregation 1-like
201636_at	2.97	-	FXR1	fragile X mental retardation, autosomal homolog 1
220048_at	2.82	-	EDAR	ectodysplasin A receptor
210766_s_at	2.75	-	CSE1L	CSE1 chromosome segregation 1-like
221567_at	2.66	-	NOL3	nucleolar protein 3 (apoptosis repressor with CARD domain)
213829_x_at	2.65	-	TNFRSF6B	tumor necrosis factor receptor superfamily, member 6b, decoy
201112_s_at	2.57	-	CSE1L	CSE1 chromosome segregation 1-like
212353_at	2.51	-	SULF1	sulfatase 1
208822_s_at	2.47	-	DAP3	death associated protein 3
209462_at	2.37	-	APLP1	amyloid beta (A4) precursor-like protein 1
203005_at	2.29	-	LTBR	lymphotoxin beta receptor (TNFR superfamily, member 3)
202731_at	4.01	+	PDCD4	programmed cell death 4
206150_at	3.57	+	TNFRSF7	tumor necrosis factor receptor superfamily, member 7
202730_s_at	3.18	+	PDCD4	programmed cell death 4
209539_at	3.14	+	ARHGEF6	Rac/Cdc42 guanine nucleotide exchange factor (GEF) 6
212593_s_at	3.07	+	PDCD4	programmed cell death 4
204933_s_at	2.96	+	TNFRSF11B	tumor necrosis factor receptor superfamily, member 11b
209831_x_at	2.43	+	DNASE2	deoxyribonuclease II, lysosomal
203187_at	2.38	+	DOCK1	dedicator of cytokinesis 1
210164_at	2.34	+	GZMB	granzyme B

Genes were sorted based on their "z-score" (significance), reflecting their association with distant metastasis-free survival time ("DMFS") time.

**Table 4:** Significant genes in the Regulation of cell cycle pathway in ER-positive tumors

Probe Set	z-score	DMFS	Gene Symbol	Gene Title
204817_at	3.73	-	ESPL1	extra spindle poles like 1 (S. cerevisiae)
38158_at	3.41	-	ESPL1	extra spindle poles like 1 (S. cerevisiae)
214710_s_at	3.10	-	CCNB1	cyclin B1
212426_s_at	3.08	-	YWHAQ	tyrosine 3-/tryptophan 5-monooxygenase activation protein
204009_s_at	3.08	-	KRAS	v-Ki-ras2 Kirsten rat sarcoma viral oncogene homolog
204947_at	3.04	-	E2F1	E2F transcription factor 1
201947_s_at	3.04	-	CCT2	chaperonin containing TCP1, subunit 2 (beta)
204822_at	2.91	-	TTK	TTK protein kinase
209096_at	2.57	-	UBE2V2	ubiquitin-conjugating enzyme E2 variant 2
204826_at	2.53	-	CCNF	cyclin F
212022_s_at	2.46	-	MKI67	antigen identified by monoclonal antibody Ki-67
202647_s_at	2.42	-	NRAS	neuroblastoma RAS viral (v-ras) oncogene homolog
201076_at	3.09	+	NHP2L1	NHP2 non-histone chromosome protein 2-like 1 (S. cerevisiae)
201601_x_at	3.00	+	IFITM1	interferon induced transmembrane protein 1 (9–27)
204015_s_at	2.90	+	DUSP4	dual specificity phosphatase 4
220407_s_at	2.68	+	TGFB2	transforming growth factor, beta 2
206404_at	2.38	+	FGF9	fibroblast growth factor 9 (glia-activating factor)

Genes were sorted based on their "z-score" (significance), reflecting their association with distant metastasis-free survival time ("DMFS") time.

**Table 5:** Significant genes in the Regulation of cell growth pathway in ER-negative tumors

Probe Set	z-score	DMFS	Gene Symbol	Gene Title
209648_x_at	4.01	-	SOCS5	suppressor of cytokine signaling 5
208127_s_at	3.75	-	SOCS5	suppressor of cytokine signaling 5
209550_at	3.18	-	NDN	necdin homolog (mouse)
201162_at	3.14	-	IGFBP7	insulin-like growth factor binding protein 7
213910_at	2.87	-	IGFBP7	insulin-like growth factor binding protein 7
212279_at	2.91	+	MAC30	hypothetical protein MAC30
213337_s_at	2.88	+	SOCS1	suppressor of cytokine signaling 1

Genes were sorted based on their "z-score" (significance), reflecting their association with distant metastasis-free survival time ("DMFS") time.

**Table 6:** Significant genes in the Regulation of G-protein coupled receptor signaling pathway in ER-negative tumors

Probe Set	z-score	DMFS	Gene Symbol	Gene Title
204337_at	3.99	-	RGS4	regulator of G-protein signalling 4
209324_s_at	3.73	-	RGS16	regulator of G-protein signalling 16
220300_at	2.61	-	RGS3	regulator of G-protein signalling 3
202388_at	2.61	-	RGS2	regulator of G-protein signalling 2, 24 kDa
204396_s_at	2.34	-	GRK5	G protein-coupled receptor kinase 5

Genes were sorted based on their "z-score" (significance), reflecting their association with distant metastasis-free survival time ("DMFS") time.

### Pathway analysis of published prognostic gene signatures

To compare genes from various prognostic signatures for breast cancer, five published gene signatures were selected [3,8,23,25,26]. We first compared the gene sequence identity between each pair of the gene signatures and found, consistent with previous reports, very few overlapping genes (Table 7). The grade index gene expression signature comprising 97 genes, of which most are associated with cell cycle regulation and proliferation [23], showed the highest number of overlapping genes between the various signatures ranging from 5 of the 16 genes of Genomic Health [25] to 10 with Yu's 62 genes [26]. The other 4 gene signatures showed only 1 gene overlap in a pair-wise comparison, and there was no common gene for all signatures. In spite of the low number of overlapping genes across signatures, we hypothesized that the representation of common pathways in the various signatures may underlie their individual prognostic value [8]. Therefore, we examined the representation of the core prognostic pathways (Table 2) in the 5 signatures. The Genomic Health 16-gene signature mapped to 10 of the 36 distinct core pathways (20 for both ER-positive and -negative tumors but counting the 4 overlapping pathways once) whereas it mapped to a total of 25 out of 304 GOBPs. The statistical significance for the enrichment of GOBP, as computed by hypergeometric distribution probability was 2 × 10^-5^. Each of the other 4 signatures have 62 or more genes and were mapped to 19 (53%) distinct prognostic pathways and their statistical significance of enrichment was 1 × 10^-7 ^for Wang and van 't Veer, 1 × 10^-6 ^for Sotiriou and 6 × 10^-11 ^for Yu's signature (Table 8). Of these 19 pathways, 9 were identical for all 4 signatures, i.e., "mitosis", "apoptosis", "regulation of cell cycle", "DNA repair", "cell cycle", "protein amino acid phosphorylation", "DNA replication", "intracellular signaling cascade", and "cell adhesion".

**Table 7:** Number of common genes between different gene signatures for breast cancer prognosis

	Wang's 76 genes	van 't Veer's 70 genes	Paik's 16 genes	Yu's 62 genes
Wang's 76 genes^a^		CCNE2	No genes	No genes
van 't Veer's 70 genes^b^	CNNE2		SCUBE2	AA962149
Paik's 16 genes^c^	No genes	SCUBE2		BIRC5
Yu's 62 genes^a^	No genes	AA962149	BIRC5	
Sotiriou's 97 genes^a^	PLK1, FEN1, CCNE2, GTSE1, KPNA2, MLF1IP, POLQ	MELK, CENPA, CCNE2, GMPS, DC13, PRC1, NUSAP1, KNTC2	MYBL2, BIRC5, STK6, MKI67, CCNB1	URCC6, FOXM1, DLG7, DKFZp686L20222, DC13, FLJ32241, HSP1CDC21, CDC2, KIF11, EXO1

^a^Affymetrix HG-U133A Genechip

^b^Agilent Hu25K microarray

^c^No genome-wide assessment; RT-PCR

To compare genes from various prognostic signatures for breast cancer, five gene signatures were selected, the 76-gene signature [8], the 70-gene signature [3], the 16-gene signature [25], the 62-gene signature [26], and the 97-gene signature [23]. Identity of genes was determined by BLAST program when gene signatures were derived from different platforms. Except for the 97-gene expression grade index [23], which showed an overlap with 5 to 16 genes with the other gene signatures, a maximum overlap of only 1 identical gene was found between the other gene signatures. The initially reported 3-gene overlap between the 76-gene and the 70-gene prognostic signatures [8]included genes with high similarity in sequences. In this study, only genes with an identical sequence in two signatures are considered overlapped based on results from BLAST program. Therefore, CCNE2 gene is the only common gene between the two signatures.

**Table 8:** Mapping various gene signatures to core pathways

		Published gene signatures^a^				
		
Pathways	GO_ID	Wang	Van 't Veer	Paik	Yu	Sotiriou
ER-positive tumors						
Apoptosis	6915	X	X	X	X	X
Regulation of cell cycle	74	X	X	X	X	X
Protein amino acid phosphorylation	6468	X	X	X	X	X
Cytokinesis	910	X	X	X		X
Cell motility	6928				X	X
Cell cycle	7049	X	X	X	X	X
Cell surface receptor-linked signal transd.	7166			X		
Mitosis	7067	X	X	X	X	X
Intracellular protein transport	6886	X	X			X
Mitotic chromosome segregation	70	X	X			X
Ubiquitin-dependent protein catabolism	6511		X		X	X
DNA repair	6281	X	X		X	X
Induction of apoptosis	6917	X				
Immune response	6955	X			X	X
Protein biosynthesis	6412			X	X	X
DNA replication	6260	X	X		X	X
Oncogenesis	7048			X	X	X
Metabolism	8152	X	X			
Cellular defense response	6968	X			X	X
Chemotaxis	6935				X	X
						
ER-negative tumors						
Regulation of cell growth	1558		X			
Regul. of G-coupled receptor signaling	8277					
Skeletal development	1501	X	X			
Protein amino acid phosphorylation	6468	X	X	X	X	X
Cell adhesion	7155	X	X		X	X
Carbohydrate metabolism	5975	X	X			
Nuclear mRNA splicing, via spliceosome	398					
Signal transduction	7165	X	X	X	X	
Cation transport	6812					
Calciumion transport	6816					
Protein modification	6464					
Intracellular signaling cascade	7242	X	X		X	X
mRNA processing	6397					
RNA splicing	8380					
Endocytosis	6897					
Regul. of transcription from PolII promoter	6357				X	
Regulation of cell cycle	74	X	X	X		
Protein complex assembly	6461		X		X	
Protein biosynthesis	6412			X		X
Cell cycle	7049	X	X	X	X	X

^a^Published gene signatures that were studied include the 76-gene signature [8], the 70-gene signature [3], the 16-gene signature [25], the 62-gene signature [26], and the 97-gene signature [23]. Individual genes in each signature were mapped to the top 20 core pathways for ER-positive and ER-negative tumors, a cross indicates a match.

## Discussion

Gene-expression profiling for separating patients into different subtypes and risk groups have been focused on the identification of differential expression of individual genes rather than obtaining biological insight. In the present study we have used an alternative approach to identify in ER-positive and ER-negative populations of breast cancer patients the underlying biological processes associated with metastasis. Using a stringent re-sampling and permutation methodology we were able to show that indeed multiple signatures can be identified showing similar prognostic power while the genes from these different samplings have similar functions. Similar observations were made when we mapped the core prognostic pathways to 5 published prognostic signatures [3,8,23,25,26]. Thus, we showed that in spite of the low number of overlapping genes between the various published gene signatures, the signatures had many pathways in common, implying that different prognostic gene signatures represent common biology. In a recent study, comparing the prognostic performance of different gene-signatures, agreement in outcome predictions were found as well [28]. However, in contrast to our present approach, the underlying pathways were not investigated. Instead, the performance of various gene signatures on a single patient cohort, heterogeneous with respect to nodal status and adjuvant systemic therapy [29], was compared [28]. It is important to note, however, that although similar pathways are represented in various signatures, it does not necessarily mean the individual genes in a pathway are equally significant or are all similarly associated with tumor aggressiveness [see Additional files 1 and 3].

The fact that none of the 20 genes most frequently present in the 500 signatures for the ER-positive tumors were among in the top 20 core gene list of the ER-negative tumors, was not surprising and is in line with the fact that ER-subgroups of tumors are biologically very different entities [1-4,8,27]. Furthermore, although among the top 20 over-represented pathways, 4 were common for ER-positive tumors and ER-negative tumors, there were in total only 2 shared genes pointing into the same direction with respect to metastatic capability of the tumors. Both genes, *KIAA0256 *in the "protein biosynthesis" pathway and *CCNT2 *in the "cell cycle pathway", were associated with an aggressive tumor behavior. These results imply that the underlying biological processes between ER-subgroups of tumors with respect to their metastatic behavior have little if any in common. Of the top 20 core prognostic pathways for the ER-positive tumors many biological processes are related to cell division activities, immunity, signal transduction, and extrinsic apoptosis-related biological processes. The cell division-related pathways have predominantly negative correlation with survival time, while immune-related pathways have predominantly positive correlation. This indicates that ER-positive tumors with metastatic capability tend to have higher cell division rates, are more resistant to external apoptotic stimuli, and induce a poor immune reaction in the host body. In ER-positive tumors, one or more of these pathways, or genes in these pathways, have also been described to be associated with the efficacy of tamoxifen therapy in recurrent breast cancer [7], in the various prognostic signatures described in the present paper [8,23,25,26], as well as in other published signatures not specifically designed for ER-positive tumors, such as the 70-gene prognostic signature [3], the stromal signatures [30], and the hypoxia signature [31]. The differences in metastatic behavior between ER-subgroups of tumors is further substantiated by the finding that in ER-negative tumors other pathways showed the strongest involvement, including those related with cell growth regulation, possibly through JAK/STAT signaling, and modulation of G-protein receptor signal transduction, RNA splicing or processing, and ion transport. No comparison can be made with the literature since no other studies so far have described prognostic of predictive pathways specifically in ER-negative breast cancer.

We were able to construct a 50-gene signature by combining the genes from the 2 most significant ER-positive and ER-negative pathways. This signature was validated and performed well on an independent published patient cohort [23], herewith showing the feasibility to derive a gene signature from biological pathways. Although further methodology and analysis would be required to optimize the selection of such a pathway-based prognostic signature, our example provides not only a new way to derive gene signatures for cancer prognosis, but also gives insight into the distinct biological processes between subgroups of tumors.

## Conclusion

Our study for the first time applied a method that systematically evaluated the biological pathways related to patient outcomes of breast cancer and showed that various published prognostic gene signatures providing similar outcome predictions are based on the representation of largely overlapping biological processes. Identification of the key biological processes, rather than the assessment of signatures based on individual genes, allows not only to build a biological meaningful gene signature from functionally related genes, but also provides insight into the mechanism of the disease development and, as spin off, potential targets for future drug development. In this respect, as pharmacologic inhibitors for specific pathways become available for the clinic, the signatures that define tumors according to their vital pathways may provide crucial guidance for designing appropriate drug combinations [32].

## Abbreviations

AUC, area under the curve; DMFS, distant metastasis-free survival; ER, estrogen receptor. GOBP, gene ontology biological process; ROC, receiver operating characteristic.

## Competing interests

Regarding conflict of interest, three co-authors are employed by Veridex LLC, a Johnson & Johnson company (Jack Yu, Yi Zhang, Yixin Wang). Johnson & Johnson is a healthcare company that is in the business of commercialising diagnostic products. The Erasmus Medical Center (Anieta Sieuwerts, John Martens, Marcel Smid, Jan Klijn, John Foekens) was financially supported by Veridex LLC for tissue processing and isolating RNA for microarray analysis.

## Authors' contributions

JXY assisted in the study design study, carried out the data analysis and drafted the manuscript; AMS processed the tumor tissues, isolated the RNA and did the quality control; YZ JXY assisted in the study design study and carried out the data analysis; JWMM assisted in the study design and contributed to the drafting of the manuscript; MS contributed to the data analysis; JGMK assisted in the collection of the clinical data and the study design; YW assisted in the study design, data analysis and drafting of the manuscript; JAF assisted in the study design, provided the clinical samples with the follow-up data, and contributed to the drafting of the manuscript. All authors have read and approved the final paper.

## Pre-publication history

The pre-publication history for this paper can be accessed here:



## Supplementary Material

Additional file 1 Top 20 prognostic pathways in ER-positive tumors. The data provided represent the results of the Geneplot function in the Global test program. The contribution of each individual gene in the top 20 prognostic pathways with distant metastasis-free survival in ER-positive tumors is plotted. Click here for file

Additional file 2 Significant genes in the top 20 prognostic pathways for ER-positive tumors. The data provided represent the contribution, standard deviation, and z-scores of each individual gene with distant metastasis-free survival in ER-positive tumors in the top 20 prognostic pathways. Click here for file

Additional file 3 Top 20 prognostic pathways in ER-negative tumors. The data provided represent the results of the Geneplot function in the Global test program. The contribution of each individual gene in the top 20 prognostic pathways with distant metastasis-free survival in ER-negative tumors is plotted. Click here for file

Additional file 4 Significant genes in the top 20 prognostic pathways for ER-negative tumors. The data provided represent the contribution, standard deviation, and z-scores of each individual gene with distant metastasis-free survival in ER-negative tumors in the top 20 prognostic pathways. Click here for file
