# Supplementary material for: Pathway analysis of gene signatures predicting metastasis of node-negative primary breast cancer
Source: BMC Cancer. 2007 Sep 25;7:182. doi: 10.1186/1471-2407-7-182 (PMC2077336; doi:10.1186/1471-2407-7-182)
Supplement: Additional file 1 — Top 20 prognostic pathways in ER-positive tumors. The data provided represent the results of the Geneplot function in the Global test program. The contribution of each individual gene in the top 20 prognostic pathways with distant metastasis-free survival in ER-positive tumors is plotted. [file 1471-2407-7-182-S1.pdf]

## Additional file 1: Top 20 prognostic pathways in ER-positive tumors

Association of the expression of individual genes with DMFS time for selected over-represented pathways. Geneplot function in the Global Test program [1, 2] was applied and the contribution of the individual genes in each selected pathway is plotted. The numbers at the X-axis represent the number of genes in the respective pathway in ER-positive tumors. The values at the Y-axis, represent the contribution (influence) of each individual gene in the selected pathway with DMFS. Negative values indicate there is no association between the gene expression and DMFS. Each thin horizontal line in a bar (influence) indicates one standard deviation away from the reference point, two or more horizontal lines in a bar indicates that the association of the corresponding gene with DMFS is statistically significant. The green bars reflect genes that are positively associated with DMFS, indicating a higher expression in tumors without metastatic capability. The red bars reflect genes that are negatively associated with DMFS, indicative of higher expression in tumors with metastatic capability. The individual positive and or negative contribution of the significant genes are presented in Addional file 2.

### References

1. Goeman JJ, van de Geer SA, de Kort F, van Houwelingen HC: **A global test for groups of genes: testing association with a clinical outcome.** *Bioinformatics* 2004, **20:**93-99.
2. Goeman JJ, Oosting J, Cleton-Jansen AM, Anninga JK, van Houwelingen HC: **Testing association of a pathway with survival using gene expression data.** *Bioinformatics* 2005, **21:**1950-1957.

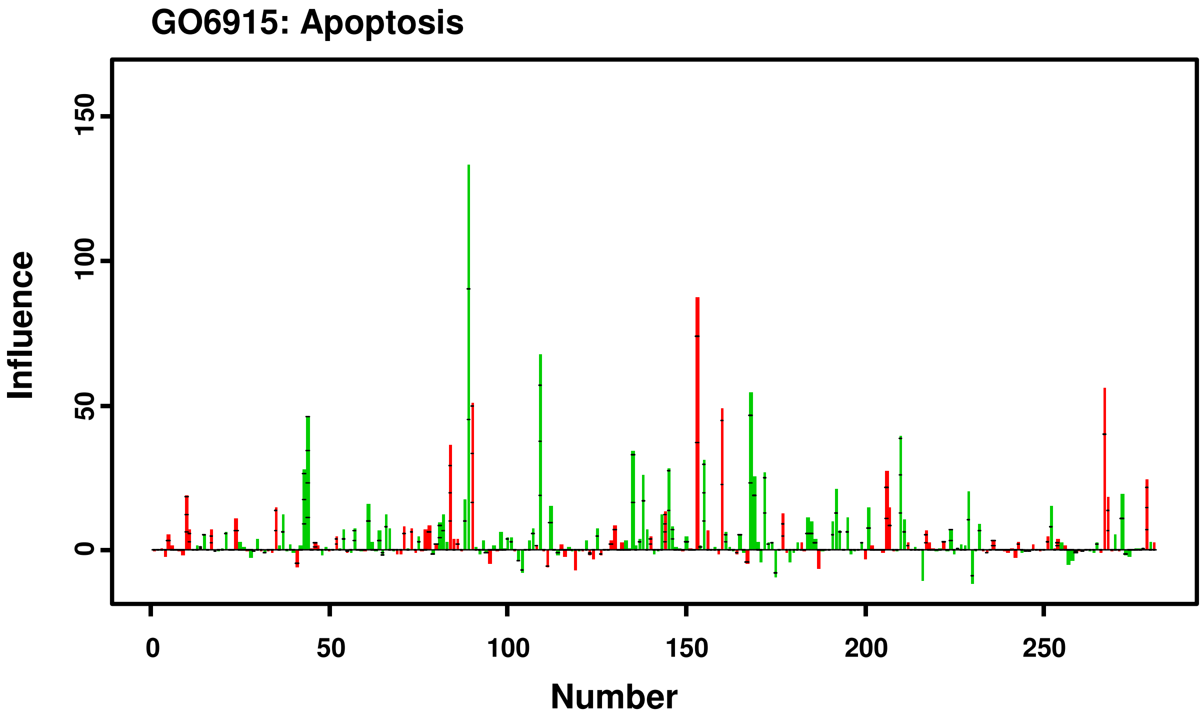

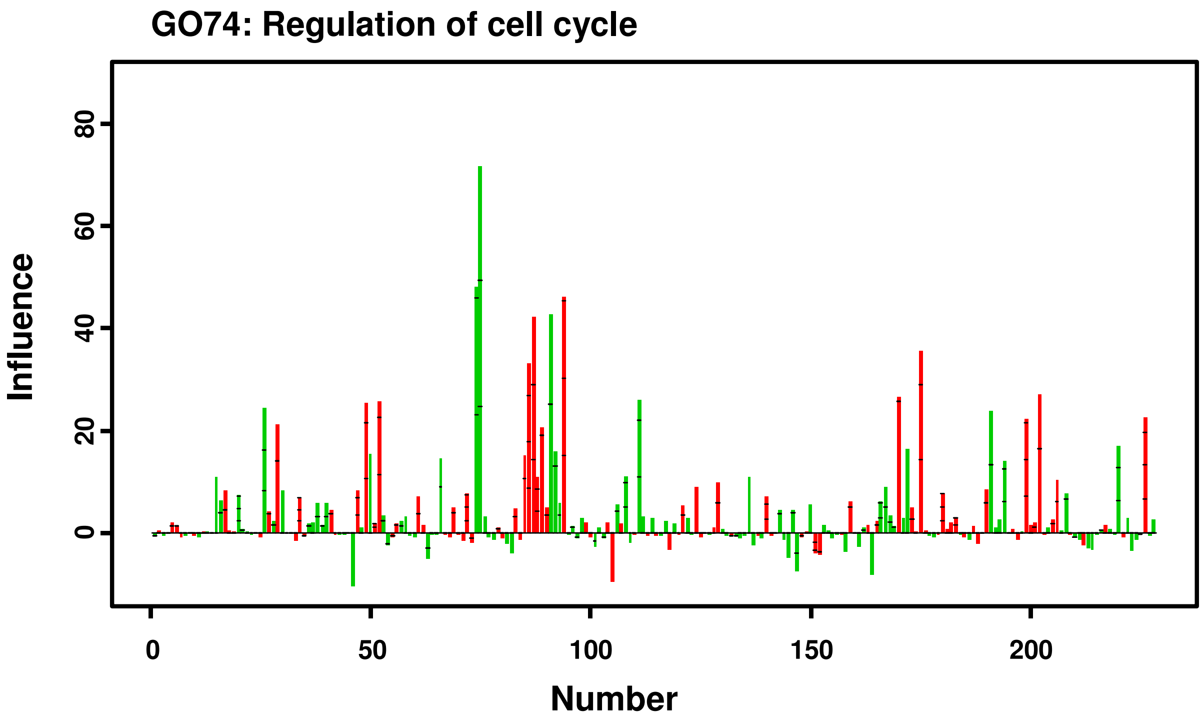
GO74: Regulation of cell cycle
Influence
0
20
40
60
80
0
50
100
150
200
Number

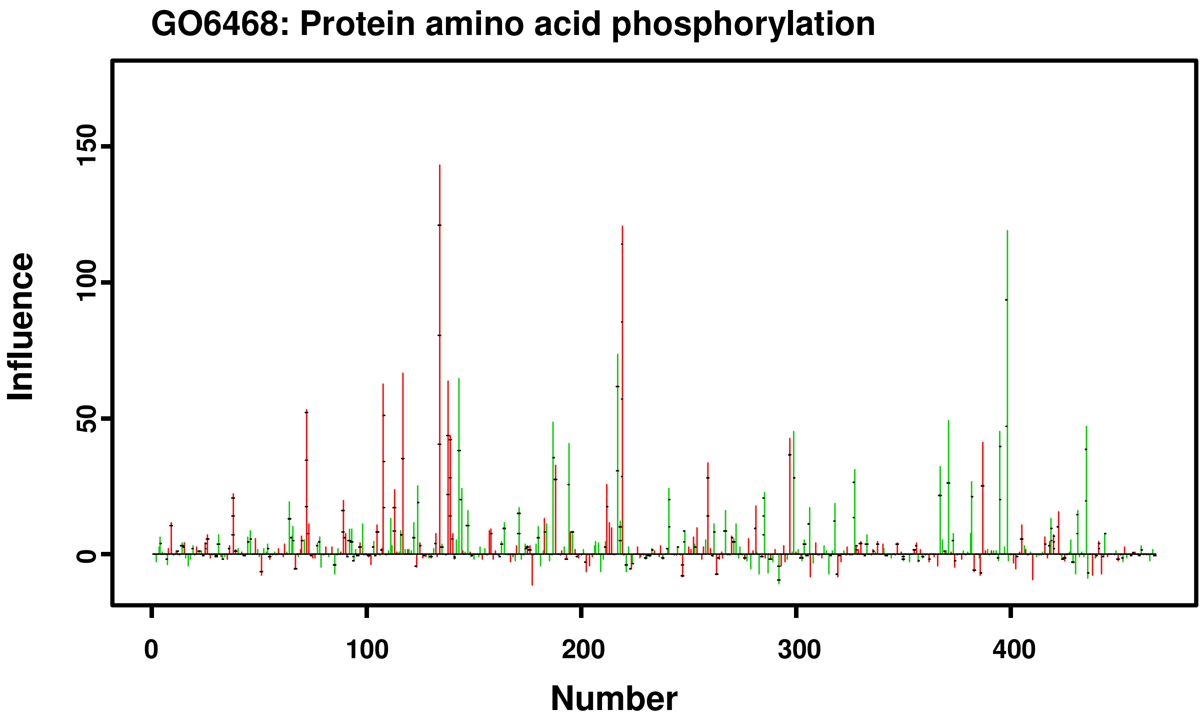
GO6468: Protein amino acid phosphorylation
Influence
0
50
100
150
0
100
200
300
400
Number

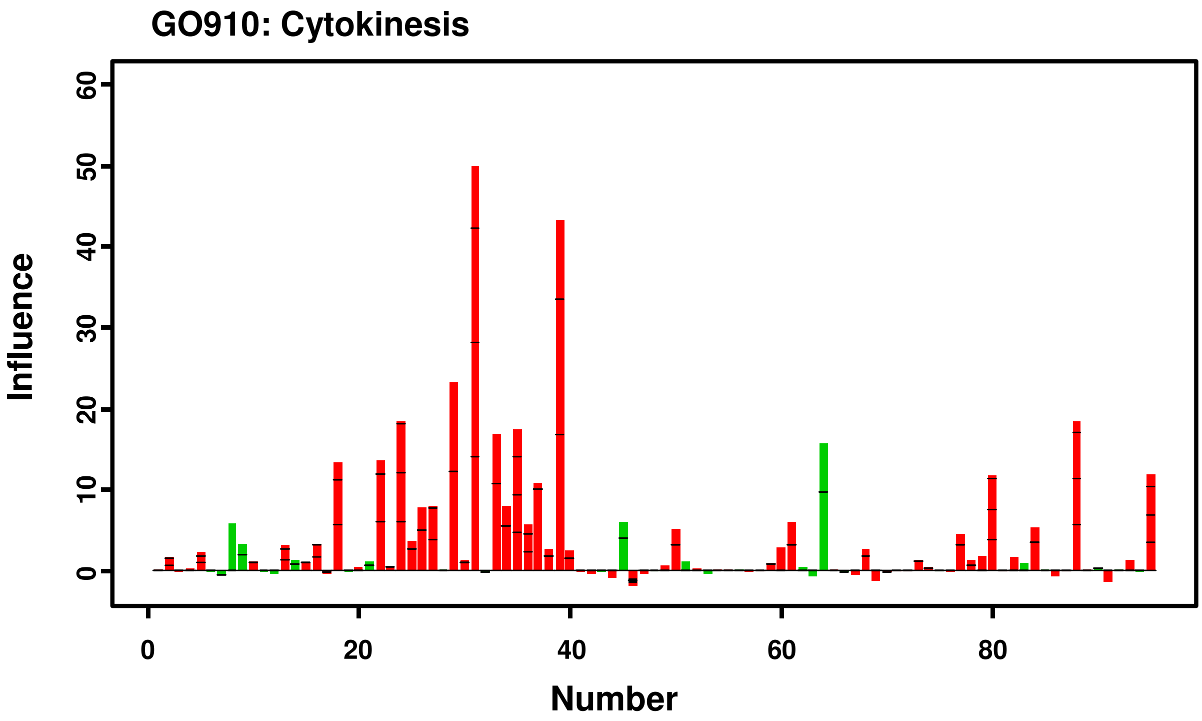

GO910: Cytokinesis
Influence
0
10
20
30
40
50
60
0
20
40
60
80
Number


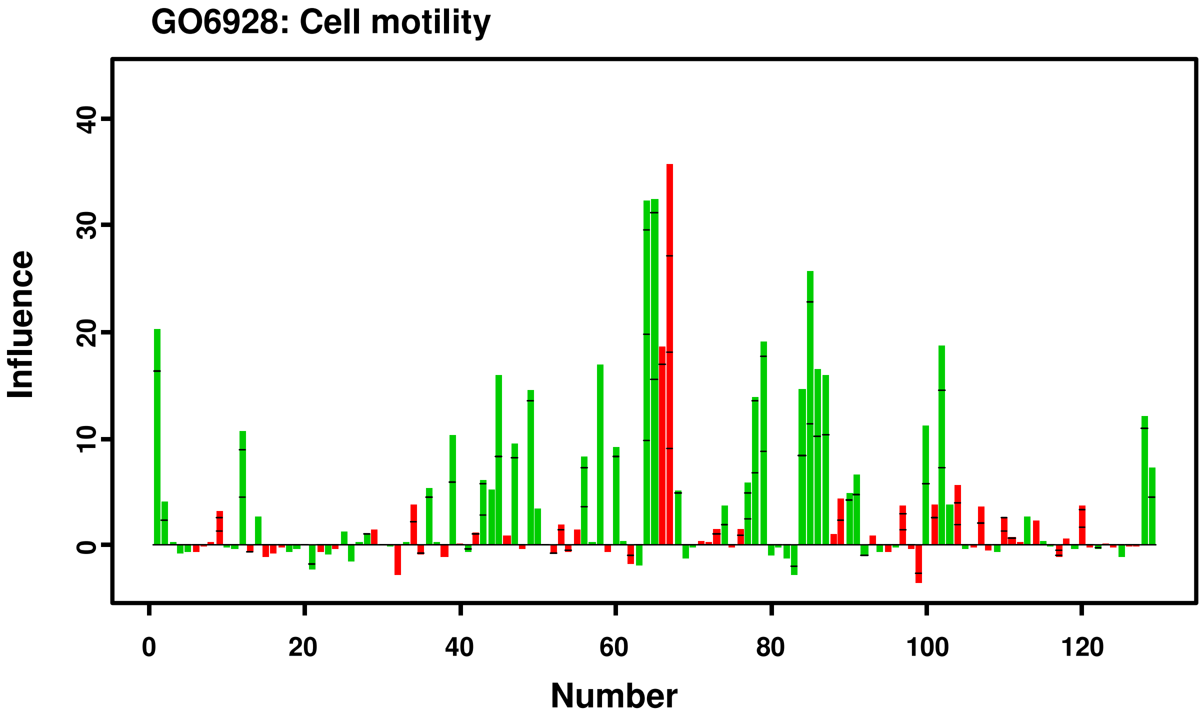

GO6928: Cell motility
Influence
0
10
20
30
40
0
20
40
60
80
100
120
Number

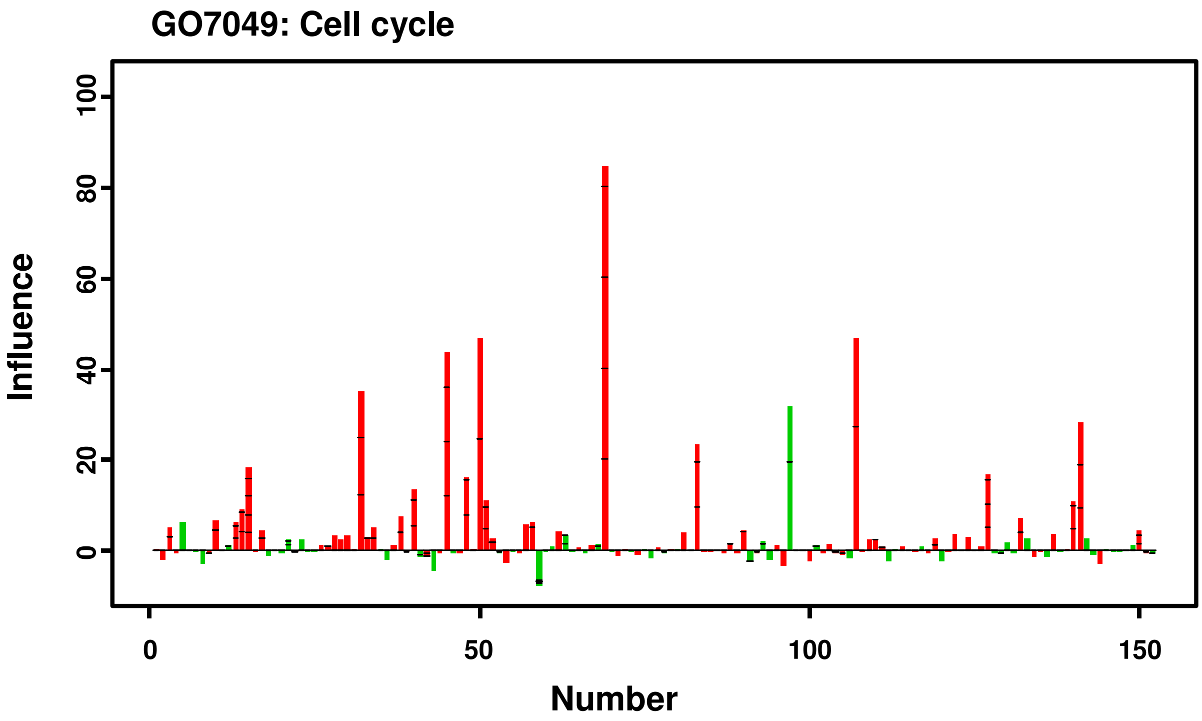

GO7049: Cell cycle
Influence
Number


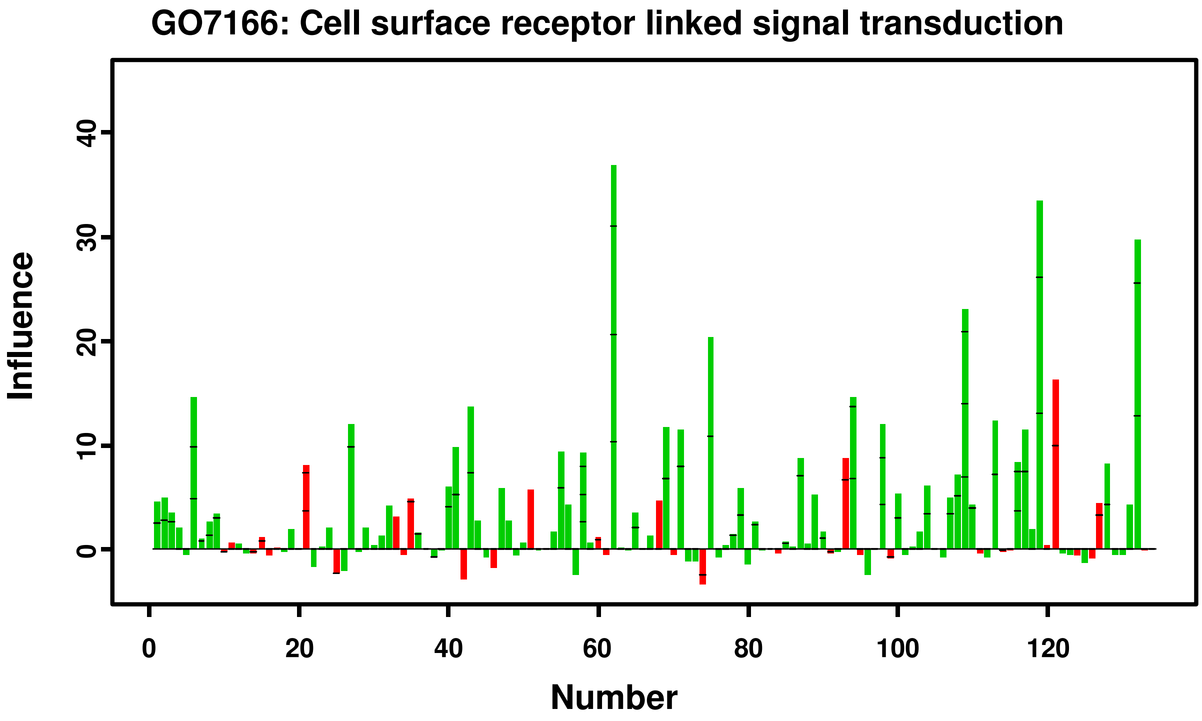

GO7166: Cell surface receptor linked signal transduction
Influence
Number

## GO7067: Mitosis

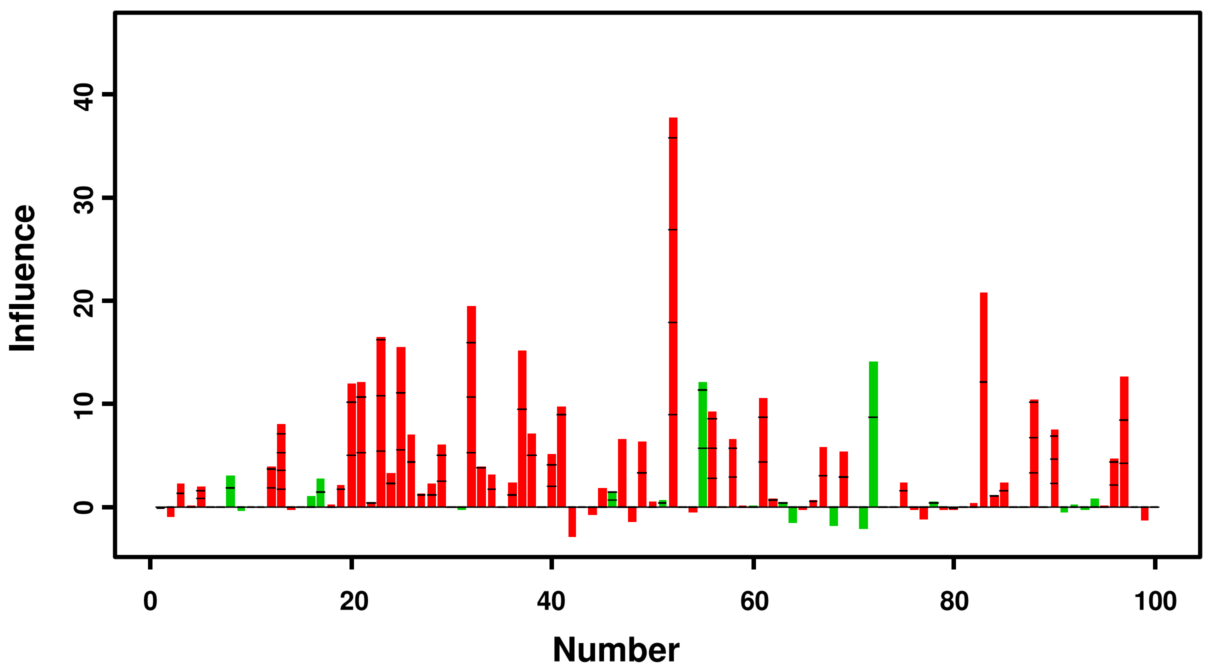


## GO6886: Intracellular protein transport

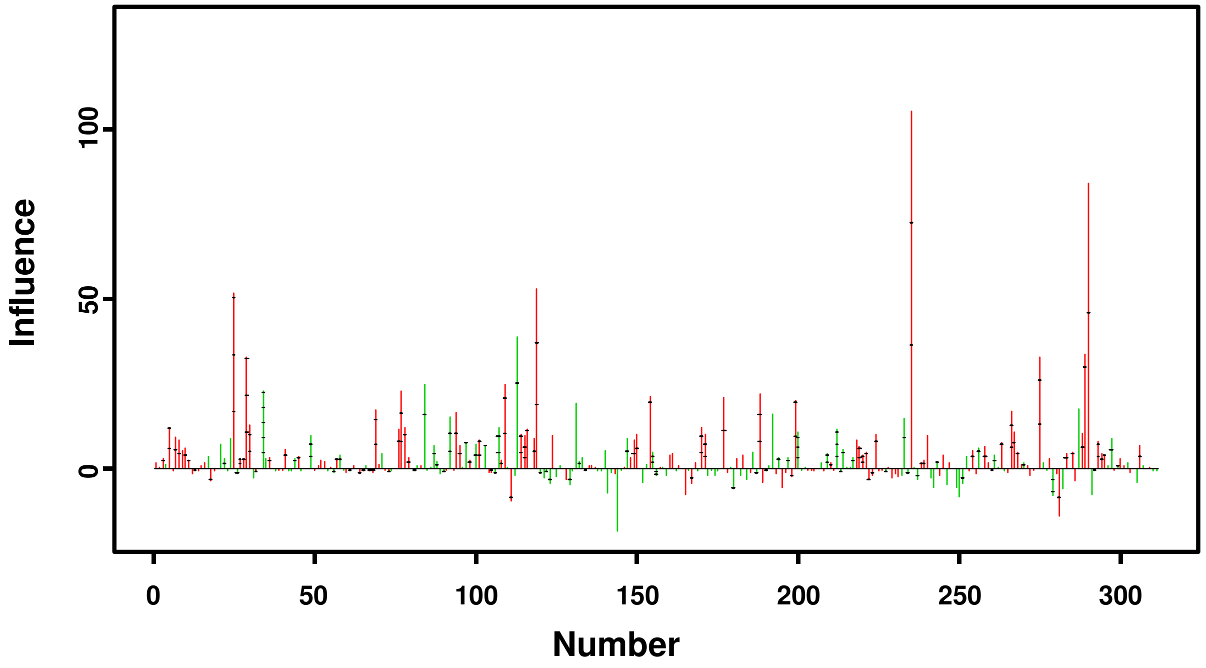

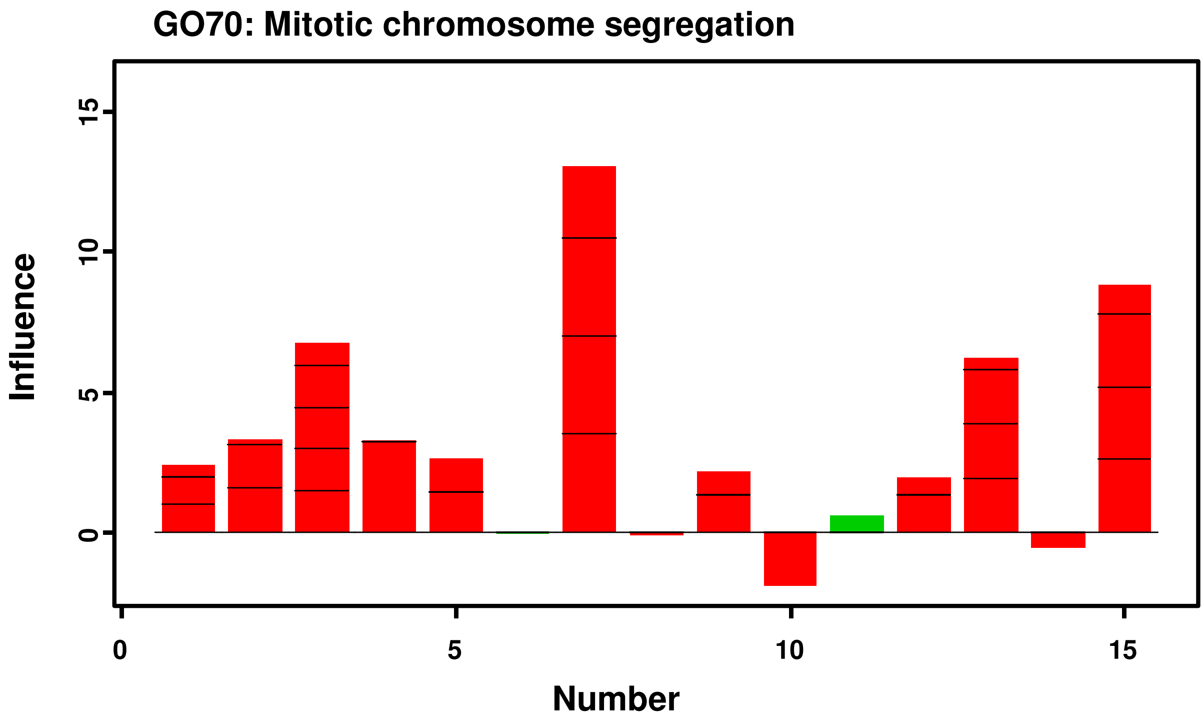

GO70: Mitotic chromosome segregation
Influence
0
5
10
15
Number


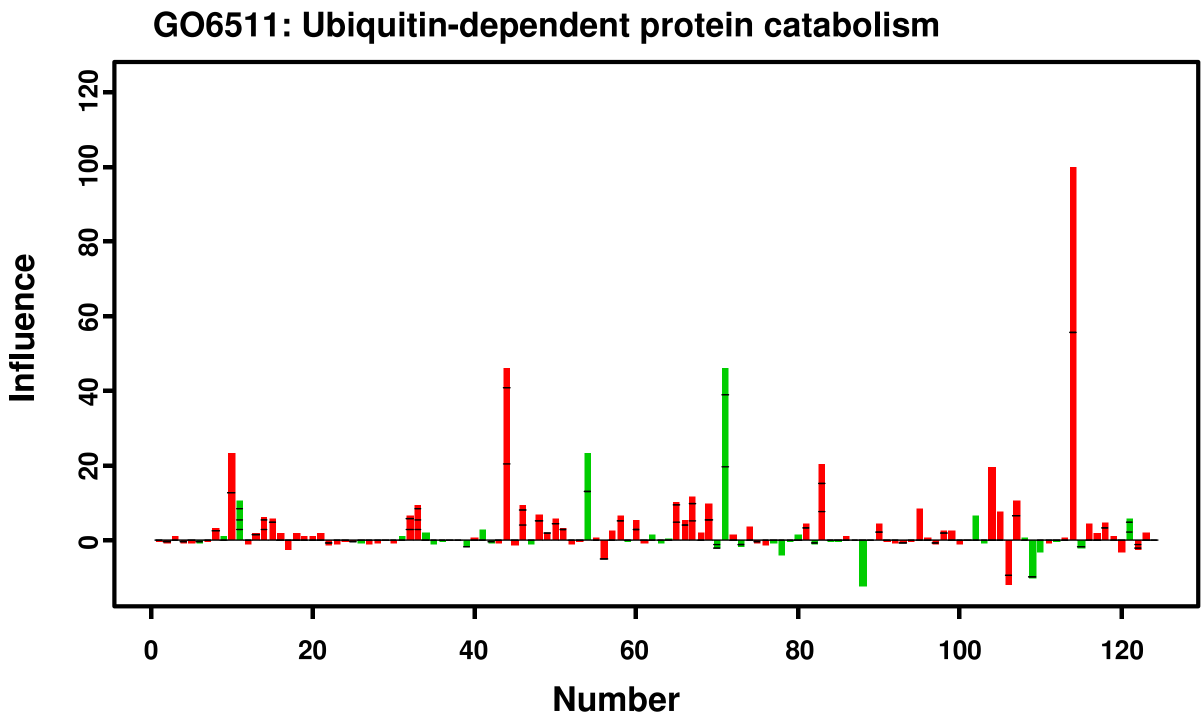

GO6511: Ubiquitin-dependent protein catabolism
Influence
0
20
40
60
80
100
120
Number

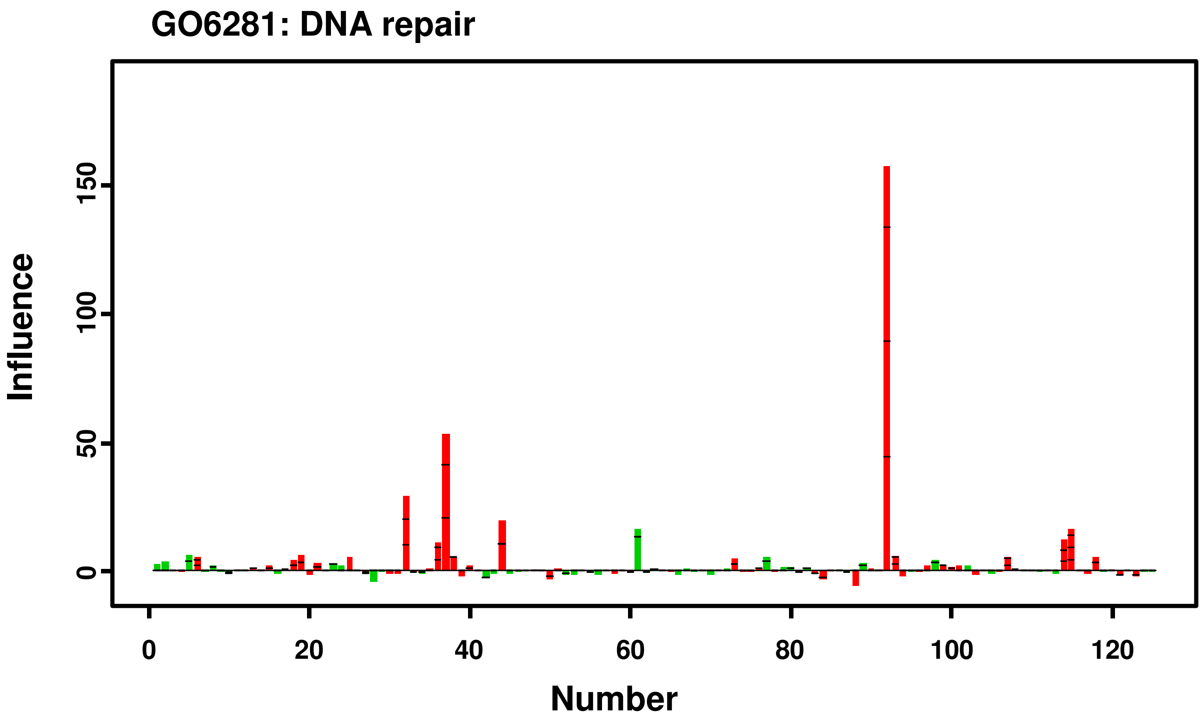
GO6281: DNA repair
Influence
Number
0
50
100
150
0
20
40
60
80
100
120

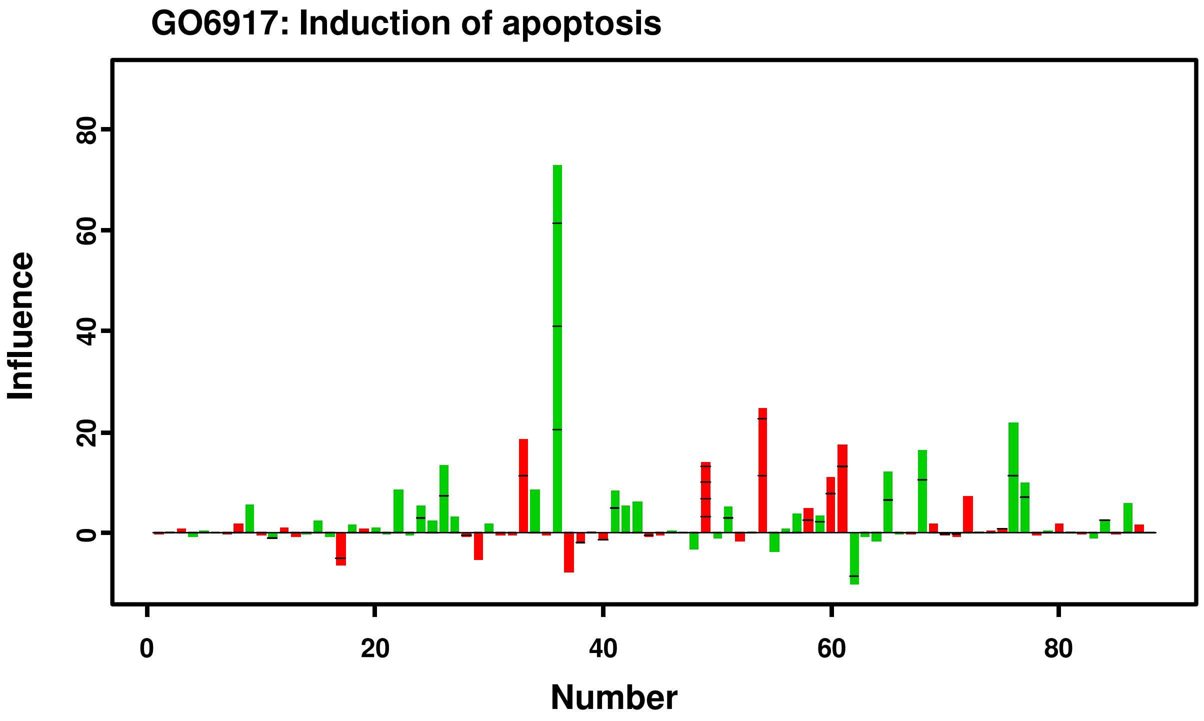
GO6917: Induction of apoptosis
Influence
Number
0
20
40
60
80
0
20
40
60
80

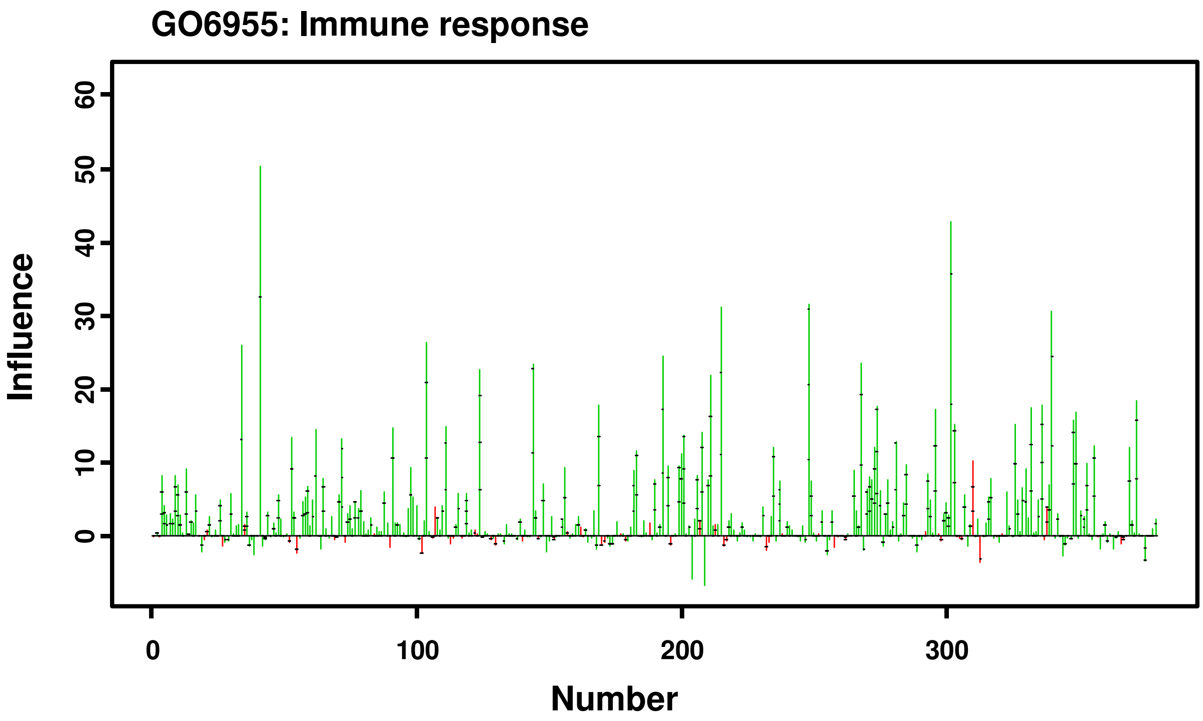
GO6955: Immune response
Influence
60
50
40
30
20
10
0
0
100
200
300
Number

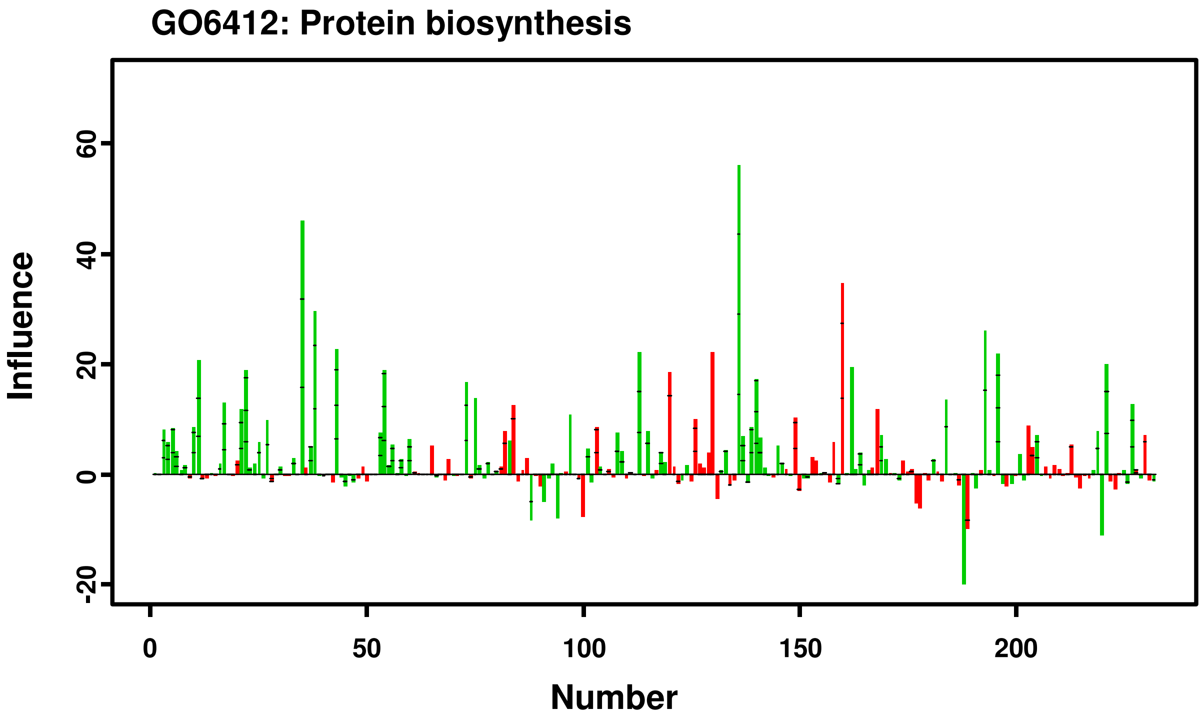
GO6412: Protein biosynthesis
Influence
60
40
20
0
-20
0
50
100
150
200
Number

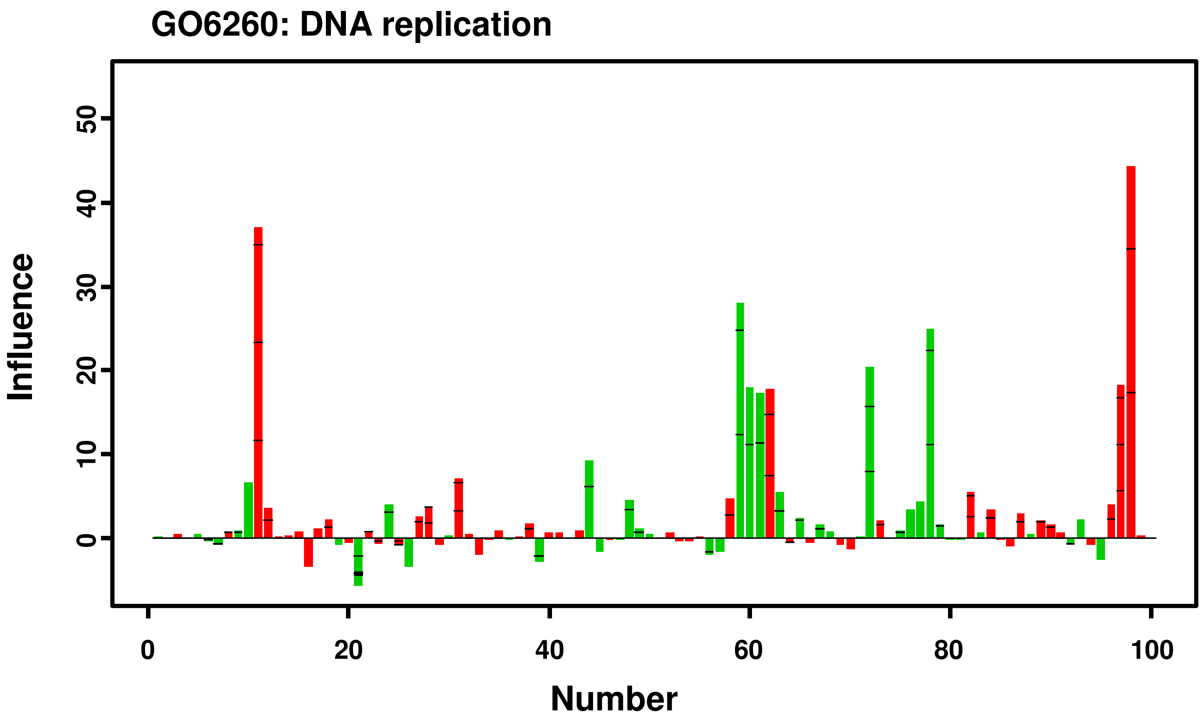
GO6260: DNA replication
Influence
Number
0
10
20
30
40
50
0
20
40
60
80
100

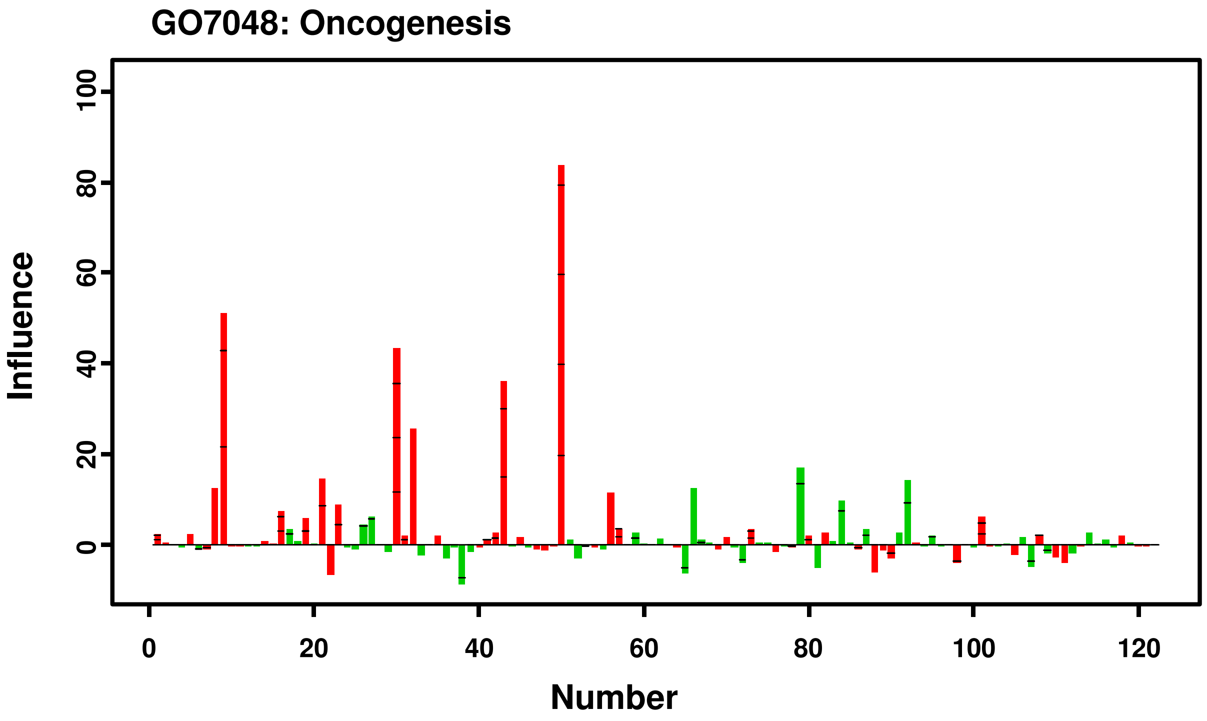
GO7048: Oncogenesis
Influence
Number
0
20
40
60
80
100
0
20
40
60
80
100
120

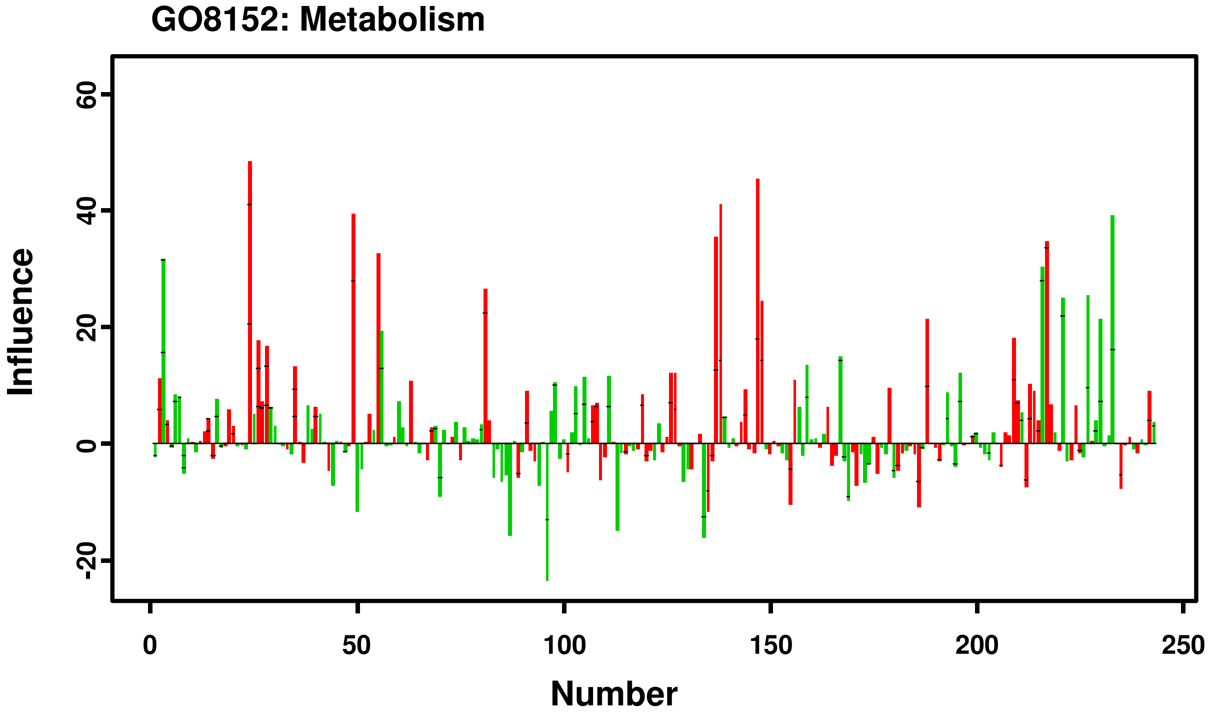

GO8152: Metabolism
Influence
Number
60
40
20
0
-20
0
50
100
150
200
250


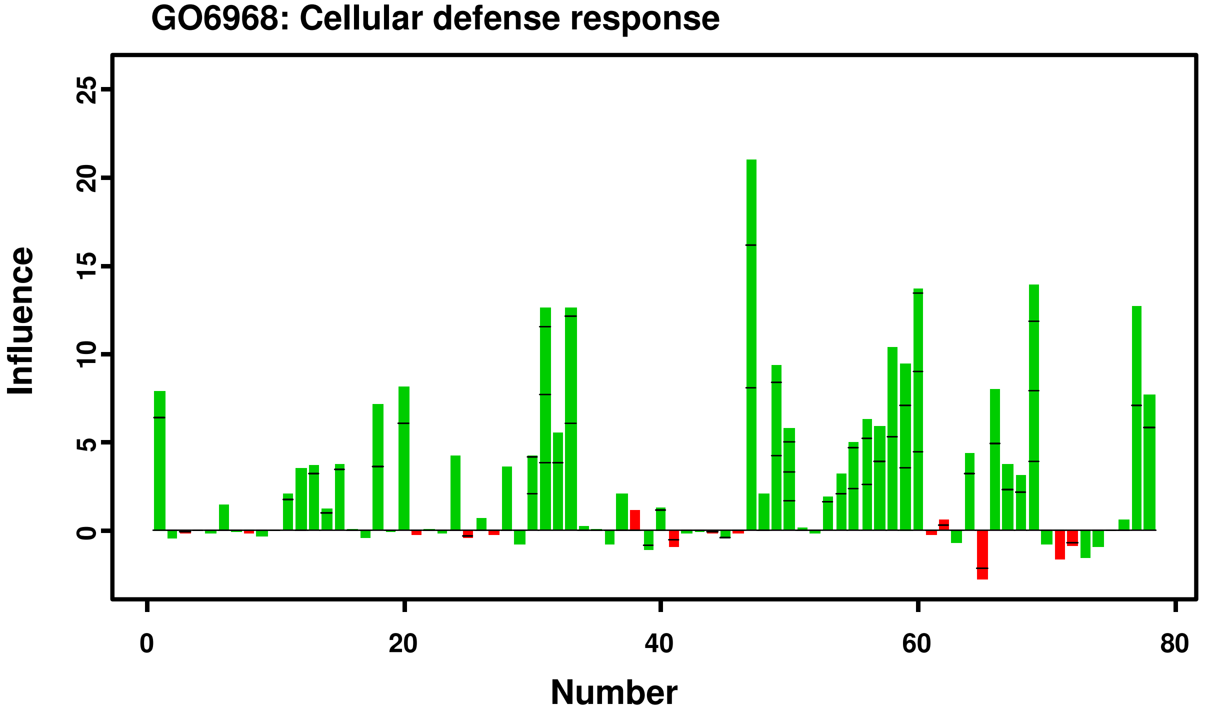

GO6968: Cellular defense response
Influence
Number
25
20
15
10
5
0
0
20
40
60
80

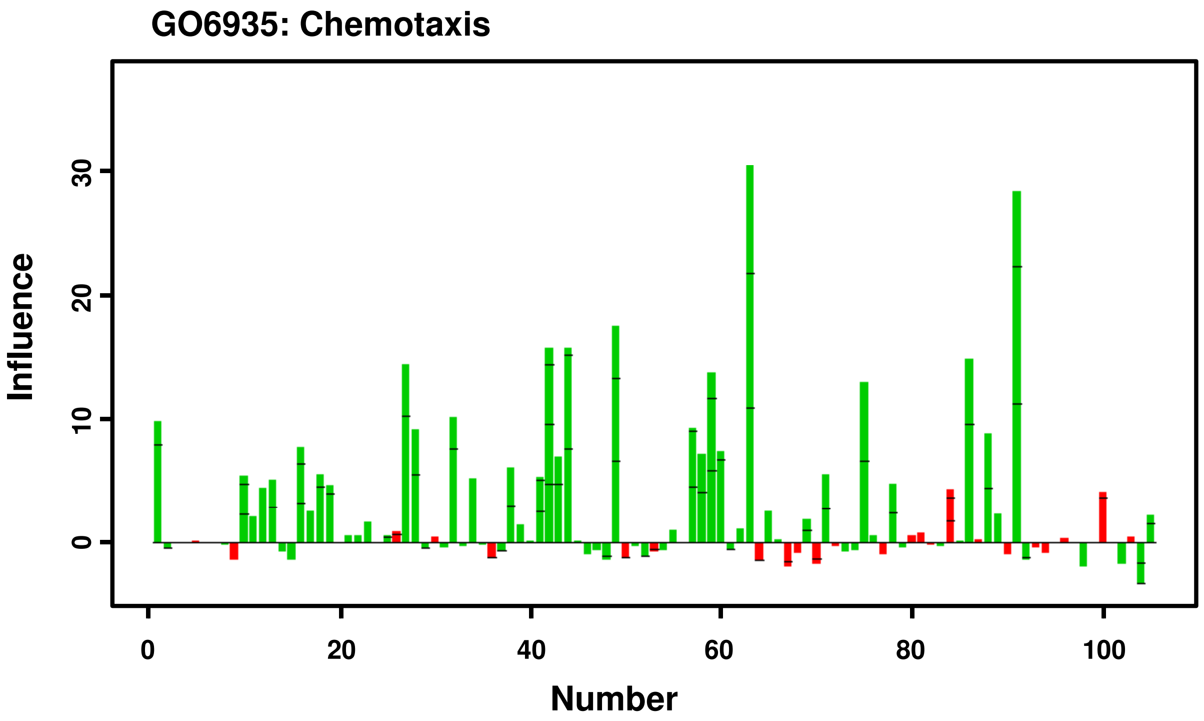
GO6935: Chemotaxis
Influence
30
20
10
0
0
20
40
60
80
100
Number
