# Supplementary material for: Pathway analysis of gene signatures predicting metastasis of node-negative primary breast cancer
Source: BMC Cancer. 2007 Sep 25;7:182. doi: 10.1186/1471-2407-7-182 (PMC2077336; doi:10.1186/1471-2407-7-182)
Supplement: Additional file 2 — Significant genes in the top 20 prognostic pathways for ER-positive tumors. The data provided represent the contribution, standard deviation, and z-scores of each individual gene with distant metastasis-free survival in ER-positive tumors in the top 20 prognostic pathways. [file 1471-2407-7-182-S2.pdf]

Additional file 2: Significant genes in the top 20 prognostic pathways for ER-positive tumors

Association of the expression of individual genes with distant metastasis-free survival (DMFS) time for selected over-represented pathways in ER-positive tumors was studied with the Geneplot function in the Global Test program [1, 2]. The contribution ("Influence") and standard deviation ("SD") of the significant genes (with "z-score" ≥1.96) of those presented in Additional file 1, are listed. The "+" sign in the "DMFS" column reflects a positive association with DMFS time, indicating a higher expression in tumors without metastatic capability. The "-" sign in the "DMFS" column reflects a negative association with DMFS time, indicative of higher expression in tumors with metastatic capability.

References

1. Goeman JJ, van de Geer SA, de Kort F, van Houwelingen HC: **A global test for groups of genes: testing association with a clinical outcome.** *Bioinformatics* 2004, **20:**93-99.
2. Goeman JJ, Oosting J, Cleton-Jansen AM, Anninga JK, van Houwelingen HC: **Testing association of a pathway with survival using gene expression data.** *Bioinformatics* 2005, **21:**1950-1957.

Probe set	Influence	SD	z-score	DMFS	Gene Symbol	Gene Title
**Apoptosis**						
208905_at	13.03	3.04	4.29	-	CYCS	cytochrome c, somatic
202731_at	46.15	11.50	4.01	+	PDCD4	programmed cell death 4
204817_at	36.39	9.77	3.73	-	ESPL1	extra spindle poles like 1
206150_at	67.60	18.92	3.57	+	TNFRSF7	tumor necrosis factor receptor superfamily, member 7
38158_at	24.65	7.23	3.41	-	ESPL1	extra spindle poles like 1
202730_s_at	27.75	8.73	3.18	+	PDCD4	programmed cell death 4
209539_at	31.06	9.89	3.14	+	ARHGEF6	Rac/Cdc42 guanine nucleotide exchange factor (GEF) 6
212593_s_at	39.35	12.82	3.07	+	PDCD4	programmed cell death 4
204947_at	50.65	16.65	3.04	-	E2F1	E2F transcription factor 1
201111_at	18.77	6.18	3.04	-	CSE1L	CSE1 chromosome segregation 1-like
201636_at	6.94	2.34	2.97	-	FXR1	fragile X mental retardation, autosomal homolog 1

204933_s_at	133.57	45.18	2.96	+	TNFRSF11B	tumor necrosis factor receptor superfamily, member 11b
220048_at	3.61	1.28	2.82	-	EDAR	ectodysplasin A receptor
210766_s_at	12.50	4.54	2.75	-	CSE1L	CSE1 chromosome segregation 1-like (yeast)
221567_at	18.12	6.81	2.66	-	NOL3	nucleolar protein 3 (apoptosis repressor with CARD domain)
213829_x_at	6.73	2.54	2.65	-	TNFRSF6B	tumor necrosis factor receptor superfamily, member 6b, decoy
201112_s_at	7.18	2.79	2.57	-	CSE1L	CSE1 chromosome segregation 1-like
212353_at	27.06	10.77	2.51	-	SULF1	sulfatase 1
208822_s_at	4.48	1.81	2.47	-	DAP3	death associated protein 3
209831_x_at	6.29	2.59	2.43	+	DNASE2	deoxyribonuclease II, lysosomal
203187_at	7.63	3.21	2.37	+	DOCK1	dedicator of cytokinesis 1
209462_at	87.55	36.92	2.37	-	APLP1	amyloid beta (A4) precursor-like protein 1
210164_at	54.43	23.24	2.34	+	GZMB	granzyme B
203005_at	4.52	1.98	2.29	-	LTBR	lymphotoxin beta receptor
209239_at	8.01	3.57	2.24	+	NFKB1	nuclear factor of kappa light polypeptide gene enhancer in B-cells 1 (p105)
202535_at	14.80	6.72	2.20	-	FADD	Fas (TNFRSF6)-associated via death domain
209803_s_at	48.69	22.44	2.17	-	PHLDA2	pleckstrin homology-like domain, family A, member 2
204513_s_at	9.17	4.29	2.14	+	ELMO1	engulfment and cell motility 1 (ced-12 homolog, C. elegans)
210538_s_at	26.69	12.54	2.13	+	BIRC3	baculoviral IAP repeat-containing 3
217840_at	3.44	1.62	2.12	-	DDX41	DEAD (Asp-Glu-Ala-Asp) box polypeptide 41
208402_at	34.33	16.37	2.10	+	IL17	interleukin 17 (cytotoxic T-lymphocyte-associated serine esterase 8)
214992_s_at	7.20	3.46	2.08	+	DNASE2	deoxyribonuclease II, lysosomal
209201_x_at	28.29	13.71	2.06	+	CXCR4	chemokine (C-X-C motif) receptor 4
2028_s_at	2.14	1.06	2.01	-	E2F1	E2F transcription factor 1
201588_at	1.13	0.56	2.01	-	TXNL1	thioredoxin-like 1
203836_s_at	6.48	3.29	1.97	+	MAP3K5	mitogen-activated protein kinase kinase kinase 5
215719_x_at	20.18	10.30	1.96	+	FAS	Fas (TNF receptor superfamily, member 6)
**Regulation of cell cycle**						
204817_at	33.18	8.90	3.73	-	ESPL1	extra spindle poles like 1
38158_at	22.48	6.60	3.41	-	ESPL1	extra spindle poles like 1
214710_s_at	22.24	7.19	3.10	-	CCNB1	cyclin B1
201076_at	7.52	2.43	3.09	+	NHP2L1	NHP2 non-histone chromosome protein 2-like 1

212426_s_at	7.86	2.55	3.08	-	YWHAQ	tyrosine 3-monooxygenase/tryptophan 5-monooxygenase activation protein
204009_s_at	7.79	2.53	3.08	-	KRAS	v-Ki-ras2 Kirsten rat sarcoma viral oncogene homolog
204947_at	46.18	15.18	3.04	-	E2F1	E2F transcription factor 1
201947_s_at	7.00	2.30	3.04	-	CCT2	chaperonin containing TCP1, subunit 2 (beta)
201601_x_at	24.46	8.16	3.00	+	IFITM1	interferon induced transmembrane protein 1 (9-27)
204822_at	42.21	14.49	2.91	-	TTK	TTK protein kinase
204015_s_at	71.73	24.75	2.90	+	DUSP4	dual specificity phosphatase 4
220407_s_at	17.06	6.36	2.68	+	TGFB2	transforming growth factor, beta 2
209096_at	7.11	2.77	2.57	-	UBE2V2	ubiquitin-conjugating enzyme E2 variant 2
204826_at	10.95	4.33	2.53	-	CCNF	cyclin F
212022_s_at	35.48	14.44	2.46	-	MKI67	antigen identified by monoclonal antibody Ki-67
202647_s_at	8.26	3.41	2.42	-	NRAS	neuroblastoma RAS viral (v-ras) oncogene homolog
206404_at	26.09	10.98	2.38	+	FGF9	fibroblast growth factor 9 (glia-activating factor)
202705_at	25.47	10.74	2.37	-	CCNB2	cyclin B2
202870_s_at	25.76	11.32	2.28	-	CDC20	CDC20 cell division cycle 20 homolog (S. cerevisiae)
205842_s_at	11.21	4.96	2.26	+	JAK2	Janus kinase 2 (a protein tyrosine kinase)
214022_s_at	13.99	6.25	2.24	+	IFITM1	interferon induced transmembrane protein 1 (9-27)
211251_x_at	6.21	2.96	2.10	+	NFYC	nuclear transcription factor Y, gamma
204014_at	48.13	23.03	2.09	+	DUSP4	dual specificity phosphatase 4
212781_at	3.04	1.50	2.02	-	RBBP6	retinoblastoma binding protein 6
2028_s_at	1.95	0.97	2.01	-	E2F1	E2F transcription factor 1
**Protein amino acid phosphorylation**						
208079_s_at	120.73	28.59	4.22	-	STK6	serine/threonine kinase 6
204092_s_at	62.39	17.05	3.66	-	STK6	serine/threonine kinase 6
204641_at	143.19	40.31	3.55	-	NEK2	NIMA (never in mitosis gene a)-related kinase 2
210754_s_at	22.18	6.89	3.22	+	LYN	v-yes-1 Yamaguchi sarcoma viral related oncogene homolog
218909_at	6.75	2.10	3.21	-	RPS6KC1	ribosomal protein S6 kinase, 52kDa, polypeptide 1
202543_s_at	21.69	6.87	3.16	-	GMFB	glia maturation factor, beta
204825_at	43.55	13.94	3.12	-	MELK	maternal embryonic leucine zipper kinase
203213_at	52.80	17.25	3.06	-	CDC2	Cell division cycle 2, G1 to S and G2 to M
204822_at	63.55	21.81	2.91	-	TTK	TTK protein kinase

204171_at	23.52	8.48	2.77	-	RPS6KB1	ribosomal protein S6 kinase, 70kDa, polypeptide 1
218764_at	12.75	4.71	2.71	+	PRKCH	protein kinase C, eta
216598_s_at	118.88	46.84	2.54	+	CCL2	chemokine (C-C motif) ligand 2
203755_at	19.43	7.95	2.44	-	BUB1B	BUB1 budding uninhibited by benzimidazoles 1 homolog beta (yeast)
208944_at	24.04	9.85	2.44	+	TGFBR2	transforming growth factor, beta receptor II (70/80kDa)
220038_at	46.82	19.30	2.43	+	SGK3	serum/glucocorticoid regulated kinase family, member 3
209642_at	33.53	13.87	2.42	-	BUB1	BUB1 budding uninhibited by benzimidazoles 1 homolog (yeast)
207957_s_at	73.49	30.64	2.40	+	ATP6AP1	ATPase, H+ transporting, lysosomal accessory protein 1
208018_s_at	11.78	5.00	2.36	+	HCK	hemopoietic cell kinase
212486_s_at	30.72	13.32	2.31	+	FYN	FYN oncogene related to SRC, FGR, YES
216033_s_at	44.93	19.72	2.28	+	FYN	FYN oncogene related to SRC, FGR, YES
205842_s_at	16.88	7.47	2.26	+	JAK2	Janus kinase 2 (a protein tyrosine kinase)
219813_at	16.04	7.16	2.24	+	LATS1	LATS, large tumor suppressor, homolog 1 (Drosophila)
220987_s_at	4.46	2.03	2.19	-	NUAK2	NUAK family, SNF1-like kinase, 2
212530_at	3.13	1.44	2.17	-	NEK7	NIMA (never in mitosis gene a)-related kinase 7
209282_at	8.49	4.15	2.04	+	PRKD2	protein kinase D2
202200_s_at	3.80	1.88	2.02	-	SRPK1	SFRS protein kinase 1
203836_s_at	8.90	4.51	1.97	+	MAP3K5	mitogen-activated protein kinase kinase kinase 5
**Cytokinesis**						
204817_at	17.44	4.68	3.73	-	ESPL1	extra spindle poles like 1
204641_at	49.99	14.07	3.55	-	NEK2	NIMA (never in mitosis gene a)-related kinase 2
38158_at	11.82	3.47	3.41	-	ESPL1	extra spindle poles like 1
218009_s_at	18.49	5.67	3.26	-	PRC1	protein regulator of cytokinesis 1
214710_s_at	11.69	3.78	3.10	-	CCNB1	cyclin B1
203213_at	18.43	6.02	3.06	-	CDC2	Cell division cycle 2, G1 to S and G2 to M
205046_at	43.34	16.80	2.58	-	CENPE	centromere protein E, 312kDa
204826_at	5.76	2.27	2.53	-	CCNF	cyclin F
201589_at	3.22	1.32	2.44	-	SMC1L1	SMC1 structural maintenance of chromosomes 1-like 1
200815_s_at	2.27	0.94	2.41	-	PAFAH1B1	platelet-activating factor acetylhydrolase, isoform Ib, alpha subunit 45kDa
202705_at	13.39	5.64	2.37	-	CCNB2	cyclin B2
200726_at	1.62	0.70	2.32	-	PPP1CC	protein phosphatase 1, catalytic subunit, gamma isoform

202870_s_at	13.54	5.95	2.28	-	CDC20	CDC20 cell division cycle 20 homolog (S. cerevisiae)
201897_s_at	3.37	1.58	2.14	-	CKS1B	CDC28 protein kinase regulatory subunit 1B
204170_s_at	8.07	3.89	2.07	-	CKS2	CDC28 protein kinase regulatory subunit 2
213743_at	1.39	0.70	1.99	-	CCNT2	cyclin T2
**Cell motility**						
207165_at	35.78	9.04	3.96	-	HMMR	hyaluronan-mediated motility receptor (RHAMM)
206983_at	32.30	9.85	3.28	+	CCR6	chemokine (C-C motif) receptor 6
211719_x_at	5.66	1.97	2.87	-	FN1	fibronectin 1
211577_s_at	18.73	7.25	2.58	+	IGF1	insulin-like growth factor 1
210495_x_at	3.69	1.49	2.47	-	FN1	fibronectin 1
208991_at	5.91	2.43	2.43	+	STAT3	signal transducer and activator of transcription 3 (acute-phase response factor)
200815_s_at	3.18	1.32	2.41	-	PAFAH1B1	platelet-activating factor acetylhydrolase, isoform Ib, alpha subunit 45kDa
200973_s_at	10.68	4.50	2.37	+	TSPAN3	tetraspanin 3
216442_x_at	3.76	1.65	2.27	-	FN1	fibronectin 1
209540_at	25.74	11.37	2.26	+	IGF1	insulin-like growth factor 1 (somatomedin C)
205842_s_at	8.27	3.66	2.26	+	JAK2	Janus kinase 2 (a protein tyrosine kinase)
209083_at	19.05	8.86	2.15	+	CORO1A	coronin, actin binding protein, 1A
204513_s_at	6.17	2.89	2.14	+	ELMO1	engulfment and cell motility 1 (ced-12 homolog, C. elegans)
207008_at	32.40	15.61	2.08	+	IL8RB	interleukin 8 receptor, beta
208992_s_at	13.84	6.76	2.05	+	STAT3	signal transducer and activator of transcription 3 (acute-phase response factor)
213101_s_at	2.59	1.28	2.03	-	ACTR3	ARP3 actin-related protein 3 homolog (yeast)
208679_s_at	3.77	1.93	1.96	+	ARPC2	actin related protein 2/3 complex, subunit 2, 34kDa
**Cell cycle**						
201664_at	18.20	4.00	4.55	-	SMC4L1	SMC4 structural maintenance of chromosomes 4-like 1
208079_s_at	84.89	20.10	4.22	-	STK6	serine/threonine kinase 6
204092_s_at	43.87	11.99	3.66	-	STK6	serine/threonine kinase 6
215623_x_at	16.82	5.18	3.25	-	SMC4L1	SMC4 structural maintenance of chromosomes 4-like 1
218663_at	28.34	9.46	2.99	-	HCAP-G	chromosome condensation protein G
203362_s_at	35.05	12.46	2.81	-	MAD2L1	MAD2 mitotic arrest deficient-like 1
32137_at	4.45	1.67	2.67	-	JAG2	jagged 2

203755_at	13.66	5.59	2.44	-	BUB1B	BUB1 budding uninhibited by benzimidazoles 1 homolog beta
201589_at	6.49	2.66	2.44	-	SMC1L1	SMC1 structural maintenance of chromosomes 1-like 1
209642_at	23.58	9.75	2.42	-	BUB1	BUB1 budding uninhibited by benzimidazoles 1 homolog
204496_at	11.23	4.77	2.35	-	STRN3	striatin, calmodulin binding protein 3
218662_s_at	10.87	4.96	2.19	-	HCAP-G	chromosome condensation protein G
201663_s_at	8.91	4.21	2.12	-	SMC4L1	SMC4 structural maintenance of chromosomes 4-like 1
204170_s_at	16.25	7.83	2.07	-	CKS2	CDC28 protein kinase regulatory subunit 2
206499_s_at	3.35	1.62	2.07	+	RCC1	regulator of chromosome condensation 1
202214_s_at	2.35	1.16	2.03	+	CUL4B	cullin 4B
213743_at	2.80	1.41	1.99	-	CCNT2	cyclin T2
**Cell surface receptor linked signal transduction**						
206150_at	36.90	10.33	3.57	+	TNFRSF7	tumor necrosis factor receptor superfamily, member 7
205926_at	9.28	2.66	3.49	+	IL27RA	interleukin 27 receptor, alpha
212587_s_at	23.07	6.96	3.32	+	PTPRC	protein tyrosine phosphatase, receptor type, C
201601_x_at	14.65	4.89	3.00	+	IFITM1	interferon induced transmembrane protein 1 (9-27)
211000_s_at	12.04	4.40	2.73	+	IL6ST	interleukin 6 signal transducer (gp130, oncostatin M receptor)
214470_at	33.53	13.03	2.57	+	KLRB1	killer cell lectin-like receptor subfamily B, member 1
222062_at	29.79	12.76	2.33	+	IL27RA	interleukin 27 receptor, alpha
214022_s_at	8.38	3.74	2.24	+	IFITM1	interferon induced transmembrane protein 1 (9-27)
202535_at	8.08	3.67	2.20	-	FADD	Fas (TNFRSF6)-associated via death domain
210538_s_at	14.57	6.84	2.13	+	BIRC3	baculoviral IAP repeat-containing 3
**Mitosis**						
201664_at	8.10	1.78	4.55	-	SMC4L1	SMC4 structural maintenance of chromosomes 4-like 1
208079_s_at	37.77	8.94	4.22	-	STK6	serine/threonine kinase 6
204092_s_at	19.52	5.33	3.66	-	STK6	serine/threonine kinase 6
215623_x_at	7.48	2.31	3.25	-	SMC4L1	SMC4 structural maintenance of chromosomes 4-like 1
209172_s_at	9.26	2.86	3.24	-	CENPF	centromere protein F, 350/400ka (mitosin)
214710_s_at	10.47	3.38	3.10	-	CCNB1	cyclin B1
203213_at	16.52	5.40	3.06	-	CDC2	Cell division cycle 2, G1 to S and G2 to M
218663_at	12.61	4.21	2.99	-	HCAP-G	chromosome condensation protein G

203362_s_at	15.59	5.55	2.81	-	MAD2L1	MAD2 mitotic arrest deficient-like 1
204826_at	5.16	2.04	2.53	-	CCNF	cyclin F
203755_at	6.08	2.49	2.44	-	BUB1B	BUB1 budding uninhibited by benzimidazoles 1 homolog beta
209642_at	10.49	4.34	2.42	-	BUB1	BUB1 budding uninhibited by benzimidazoles 1 homolog
200815_s_at	2.03	0.84	2.41	-	PAFAH1B1	platelet-activating factor acetylhydrolase, isoform Ib, alpha subunit 45kDa
202705_at	12.00	5.06	2.37	-	CCNB2	cyclin B2
209408_at	6.66	2.87	2.32	-	KIF2C	kinesin family member 2C
202870_s_at	12.13	5.33	2.28	-	CDC20	CDC20 cell division cycle 20 homolog (S. cerevisiae)
218662_s_at	4.83	2.21	2.19	-	HCAP-G	chromosome condensation protein G
209083_at	12.16	5.65	2.15	+	CORO1A	coronin, actin binding protein, 1A
201663_s_at	3.97	1.87	2.12	-	SMC4L1	SMC4 structural maintenance of chromosomes 4-like 1
206499_s_at	1.49	0.72	2.07	+	RCC1	regulator of chromosome condensation 1
**Intracellular protein transport**						
201216_at	22.62	4.46	5.07	+	ERP29	endoplasmic reticulum protein 29
211779_x_at	10.48	3.08	3.40	+	AP2A2	adaptor-related protein complex 2, alpha 2 subunit
212159_x_at	11.53	3.60	3.21	+	AP2A2	adaptor-related protein complex 2, alpha 2 subunit
201088_at	51.35	16.82	3.05	-	KPNA2	karyopherin alpha 2
201111_at	32.61	10.74	3.04	-	CSE1L	CSE1 chromosome segregation 1-like
204478_s_at	9.39	3.13	3.00	-	RABIF	RAB interacting factor
203311_s_at	15.15	5.20	2.91	+	ARF6	ADP-ribosylation factor 6
214337_at	105.30	36.24	2.91	-	COPA	coatomer protein complex, subunit alpha
204974_at	52.86	18.62	2.84	-	RAB3A	RAB3A, member RAS oncogene family
202630_at	22.63	8.05	2.81	-	APPBP2	amyloid beta precursor protein (cytoplasmic tail) binding protein 2
208819_at	4.68	1.68	2.78	+	RAB8A	RAB8A, member RAS oncogene family
210766_s_at	21.71	7.89	2.75	-	CSE1L	CSE1 chromosome segregation 1-like
209268_at	9.70	3.53	2.74	-	VPS45A	vacuolar protein sorting 45A
201831_s_at	9.56	3.50	2.73	+	VDP	vesicle docking protein p115
218360_at	16.60	6.43	2.58	-	RAB22A	RAB22A, member RAS oncogene family
201112_s_at	12.48	4.85	2.57	-	CSE1L	CSE1 chromosome segregation 1-like
203679_at	11.96	4.69	2.55	+	TMED1	transmembrane emp24 protein transport domain containing 1
218755_at	32.63	12.95	2.52	-	KIF20A	kinesin family member 20A

209238_at	12.00	4.78	2.51	-	STX3A	syntaxin 3A
204017_at	24.75	10.31	2.40	-	KDELR3	KDEL (Lys-Asp-Glu-Leu) endoplasmic reticulum protein retention receptor 3
202395_at	16.99	7.11	2.39	-	NSF	N-ethylmaleimide-sensitive factor
221014_s_at	7.83	3.53	2.22	-	RAB33B	RAB33B, member RAS oncogene family
212652_s_at	3.70	1.73	2.14	-	SNX4	sorting nexin 4
212103_at	4.16	1.95	2.13	+	KPNA6	Karyopherin alpha 6 (importin alpha 7)
204477_at	9.92	4.67	2.13	-	RABIF	RAB interacting factor
201097_s_at	2.72	1.28	2.12	-	ARF4	ADP-ribosylation factor 4
212635_at	6.06	2.88	2.10	-	TNPO1	Transportin 1
203544_s_at	8.14	3.93	2.07	-	STAM	signal transducing adaptor molecule (SH3 domain and ITAM motif) 1
211762_s_at	19.76	9.65	2.05	-	KPNA2	karyopherin alpha 2 (RAG cohort 1, importin alpha 1)
200614_at	11.87	5.87	2.02	-	CLTC	clathrin, heavy polypeptide (Hc)
208732_at	8.12	4.07	2.00	-	RAB2	RAB2, member RAS oncogene family
200699_at	8.38	4.29	1.95	-	KDELR2	KDEL (Lys-Asp-Glu-Leu) endoplasmic reticulum protein retention receptor 2
**Mitotic chromosome segregation**						
201664_at	6.77	1.49	4.55	-	SMC4L1	SMC4 structural maintenance of chromosomes 4-like 1
204817_at	13.07	3.51	3.73	-	ESPL1	extra spindle poles like 1
38158_at	8.85	2.60	3.41	-	ESPL1	extra spindle poles like 1
215623_x_at	6.26	1.93	3.25	-	SMC4L1	SMC4 structural maintenance of chromosomes 4-like 1
201589_at	2.41	0.99	2.44	-	SMC1L1	SMC1 structural maintenance of chromosomes 1-like 1
201663_s_at	3.32	1.57	2.12	-	SMC4L1	SMC4 structural maintenance of chromosomes 4-like 1
**Ubiquitin-dependent protein catabolism**						
201178_at	10.32	2.73	3.79	+	FBXO7	F-box protein 7
202244_at	9.40	2.71	3.48	-	PSMB4	proteasome (prosome, macropain) subunit, beta type, 4
211702_s_at	20.08	7.60	2.64	-	USP32	ubiquitin specific peptidase 32
221519_at	5.75	2.22	2.58	+	FBXW4	F-box and WD-40 domain protein 4
202981_x_at	9.35	3.90	2.40	-	SIAH1	seven in absentia homolog 1 (Drosophila)
209040_s_at	46.23	19.42	2.38	+	PSMB8	proteasome (prosome, macropain) subunit, beta type, 8
208805_at	11.48	4.83	2.38	-	PSMA6	proteasome (prosome, macropain) subunit, alpha type, 6
202243_s_at	6.60	2.87	2.30	-	PSMB4	proteasome (prosome, macropain) subunit, beta type, 4

202870_s_at	46.10	20.26	2.28	-	CDC20	CDC20 cell division cycle 20 homolog (S. cerevisiae)
208760_at	10.11	4.70	2.15	-	UBE2I	Ubiquitin-conjugating enzyme E2I
201317_s_at	5.90	2.77	2.13	-	PSMA2	proteasome (prosome, macropain) subunit, alpha type, 2
**DNA repair**						
219510_at	16.77	4.57	3.67	-	POLQ	polymerase (DNA directed), theta
213520_at	157.23	44.55	3.53	-	RECQL4	RecQ protein-like 4
219502_at	12.24	4.08	3.00	-	NEIL3	nei endonuclease VIII-like 3
204146_at	29.05	10.24	2.84	-	RAD51AP1	RAD51 associated protein 1
204558_at	53.36	20.63	2.59	-	RAD54L	RAD54-like
204531_s_at	11.12	4.52	2.46	-	BRCA1	breast cancer 1, early onset
201589_at	5.45	2.23	2.44	-	SMC1L1	SMC1 structural maintenance of chromosomes 1-like 1
218397_at	5.64	2.56	2.21	-	FANCL	Fanconi anemia, complementation group L
213734_at	6.10	2.79	2.18	-	WSB2	WD repeat and SOCS box-containing 2
**Induction of apoptosis**						
208905_at	14.07	3.28	4.29	-	CYCS	cytochrome c, somatic
206150_at	72.98	20.43	3.57	+	TNFRSF7	tumor necrosis factor receptor superfamily, member 7
209448_at	24.65	11.28	2.19	-	HTATIP2	HIV-1 Tat interactive protein 2, 30kDa
209929_s_at	4.91	2.49	1.97	-	IKBKG	inhibitor of kappa light polypeptide gene enhancer in B-cells, kinase gamma
215719_x_at	21.79	11.12	1.96	+	FAS	Fas (TNF receptor superfamily, member 6)
**Immune response**						
206150_at	22.64	6.34	3.57	+	TNFRSF7	tumor necrosis factor receptor superfamily, member 7
215633_x_at	17.75	5.04	3.52	+	LST1	leukocyte specific transcript 1
205926_at	5.69	1.63	3.49	+	IL27RA	interleukin 27 receptor, alpha
210629_x_at	7.36	2.12	3.47	+	LST1	leukocyte specific transcript 1
204670_x_at	13.15	3.95	3.33	+	HLA-DRB1	major histocompatibility complex, class II, DR beta 1
211582_x_at	17.49	5.72	3.06	+	LST1	leukocyte specific transcript 1
210982_s_at	31.37	10.27	3.05	+	HLA-DRA	major histocompatibility complex, class II, DR alpha
209312_x_at	13.65	4.51	3.02	+	HLA-DRB1	major histocompatibility complex, class II, DR beta 1
213226_at	10.10	3.37	3.00	-	CCNA2	Cyclin A2

201601_x_at	8.98	3.00	3.00	+	IFITM1	interferon induced transmembrane protein 1 (9-27)
208894_at	24.35	8.56	2.84	+	HLA-DRA	major histocompatibility complex, class II, DR alpha
211991_s_at	17.17	6.07	2.83	+	HLA-DPA1	major histocompatibility complex, class II, DP alpha 1
215193_x_at	17.46	6.18	2.82	+	HLA-DRB1	major histocompatibility complex, class II, DR beta 1
217478_s_at	9.71	3.45	2.82	+	HLA-DMA	major histocompatibility complex, class II, DM alpha
210072_at	31.12	11.12	2.80	+	CCL19	chemokine (C-C motif) ligand 19
200904_at	8.21	2.98	2.76	+	HLA-E	major histocompatibility complex, class I, E
211000_s_at	7.38	2.70	2.73	+	IL6ST	interleukin 6 signal transducer (gp130, oncostatin M receptor)
211581_x_at	12.05	4.50	2.68	+	LST1	leukocyte specific transcript 1
209823_x_at	21.88	8.17	2.68	+	HLA-DQB1	major histocompatibility complex, class II, DQ beta 1
207850_at	17.82	6.79	2.63	+	CXCL3	chemokine (C-X-C motif) ligand 3
208306_x_at	8.90	3.40	2.62	+	HLA-DRB1	Major histocompatibility complex, class II, DR beta 3
203010_at	3.23	1.27	2.54	+	STAT5A	signal transducer and activator of transcription 5A
200905_x_at	3.98	1.58	2.52	+	HLA-E	major histocompatibility complex, class I, E
201288_at	6.88	2.73	2.52	+	ARHGDIB	Rho GDP dissociation inhibitor (GDI) beta
215784_at	30.48	12.17	2.50	+	CD1E	CD1E antigen, e polypeptide
205544_s_at	26.20	10.46	2.50	+	CR2	complement component (3d/Epstein Barr virus) receptor 2
211430_s_at	23.54	9.63	2.44	+	IGH	immunoglobulin heavy constant gamma 1 (G1m marker)
217456_x_at	2.67	1.09	2.44	+	HLA-E	major histocompatibility complex, class I, E
201137_s_at	8.17	3.36	2.43	+	HLA-DPB1	major histocompatibility complex, class II, DP beta 1
211529_x_at	7.99	3.32	2.41	+	HLA-G	HLA-G histocompatibility antigen, class I, G
212592_at	42.76	17.85	2.40	+	IGJ	Immunoglobulin J polypeptide
204470_at	7.85	3.30	2.38	+	CXCL1	chemokine (C-X-C motif) ligand 1
209040_s_at	9.49	3.99	2.38	+	PSMB8	proteasome (prosome, macropain) subunit, beta type, 8
209687_at	14.05	5.97	2.35	+	CXCL12	chemokine (C-X-C motif) ligand 12
222062_at	18.27	7.83	2.33	+	IL27RA	interleukin 27 receptor, alpha
205671_s_at	14.74	6.33	2.33	+	HLA-DOB	major histocompatibility complex, class II, DO beta
202748_at	4.75	2.04	2.33	+	GBP2	guanylate binding protein 2, interferon-inducible
217767_at	12.27	5.31	2.31	+	C3	complement component 3
211799_x_at	9.65	4.19	2.30	+	HLA-C	major histocompatibility complex, class I, C
203005_at	1.51	0.66	2.29	-	LTBR	lymphotoxin beta receptor (TNFR superfamily, member 3)
212203_x_at	2.79	1.22	2.28	+	IFITM3	interferon induced transmembrane protein 3 (1-8U)

203666_at	5.48	2.43	2.26	+	CXCL12	chemokine (C-X-C motif) ligand 12
214022_s_at	5.14	2.30	2.24	+	IFITM1	interferon induced transmembrane protein 1 (9-27)
217014_s_at	15.72	7.03	2.24	+	AZGP1	alpha-2-glycoprotein 1, zinc
211911_x_at	8.34	3.73	2.23	+	HLA-B	major histocompatibility complex, class I, B
210514_x_at	11.98	5.36	2.23	+	HLA-G	HLA-G histocompatibility antigen, class I, G
204116_at	6.74	3.09	2.18	+	IL2RG	interleukin 2 receptor, gamma
209619_at	8.17	3.75	2.18	+	CD74	CD74 antigen
208729_x_at	7.58	3.54	2.14	+	HLA-B	major histocompatibility complex, class I, B
207323_s_at	2.28	1.08	2.12	+	MBP	myelin basic protein
212671_s_at	15.09	7.13	2.12	+	HLA-DQA1 /// HLA-DQA2	major histocompatibility complex, class II, DQ alpha 1
211528_x_at	6.34	3.00	2.11	+	HLA-G	HLA-G histocompatibility antigen, class I, G
208402_at	11.50	5.48	2.10	+	IL17	interleukin 17
209666_s_at	2.11	1.01	2.08	-	CHUK	conserved helix-loop-helix ubiquitous kinase
209201_x_at	9.47	4.59	2.06	+	CXCR4	chemokine (C-X-C motif) receptor 4
206641_at	23.27	11.37	2.05	+	TNFRSF17	tumor necrosis factor receptor superfamily, member 17
211734_s_at	12.74	6.25	2.04	+	FCER1A	Fc fragment of IgE, high affinity I, receptor for; alpha polypeptide
204806_x_at	4.70	2.33	2.02	+	HLA-F	major histocompatibility complex, class I, F
215669_at	3.81	1.90	2.01	-	HLA-DRB4	major histocompatibility complex, class II, DR beta 4
206086_x_at	0.71	0.36	1.98	-	HFE	hemochromatosis
209929_s_at	1.52	0.77	1.97	-	IKBKG	inhibitor of kappa light polypeptide gene enhancer in B-cells, kinase gamma
202992_at	25.86	13.15	1.97	+	C7	complement component 7
214974_x_at	8.97	4.58	1.96	+	CXCL5	chemokine (C-X-C motif) ligand 5
215719_x_at	6.76	3.45	1.96	+	FAS	Fas (TNF receptor superfamily, member 6)
**Protein biosynthesis**						
211666_x_at	56.18	14.56	3.86	+	RPL3	ribosomal protein L3
217747_s_at	21.97	6.01	3.66	+	RPS9	ribosomal protein S9
200937_s_at	22.70	6.32	3.59	+	RPL5	ribosomal protein L5
200081_s_at	18.99	5.85	3.25	+	RPS6	ribosomal protein S6
201076_at	18.95	6.12	3.09	+	NHP2L1	NHP2 non-histone chromosome protein 2-like 1
211938_at	17.38	5.67	3.07	+	EIF4B	eukaryotic translation initiation factor 4B
200024_at	20.65	6.95	2.97	+	RPS5	ribosomal protein S5

208887_at	22.22	7.58	2.93	+	EIF3S4	eukaryotic translation initiation factor 3, subunit 4 delta, 44kDa
213687_s_at	7.25	2.48	2.92	+	RPL35A	ribosomal protein L35a
200036_s_at	13.18	4.52	2.91	+	RPL10A	ribosomal protein L10a
200823_x_at	46.07	15.87	2.90	+	RPL29	ribosomal protein L29
220960_x_at	20.05	7.47	2.68	+	RPL22	ribosomal protein L22
211710_x_at	6.88	2.58	2.66	+	RPL4	ribosomal protein L4
202247_s_at	16.72	6.28	2.66	+	MTA1	metastasis associated 1
200005_at	8.27	3.11	2.66	+	EIF3S7	eukaryotic translation initiation factor 3, subunit 7 zeta, 66/67kDa
200013_at	4.18	1.59	2.63	+	RPL24	ribosomal protein L24
221726_at	12.88	4.90	2.63	+	RPL22	ribosomal protein L22
201258_at	6.53	2.49	2.62	+	RPS16	ribosomal protein S16
213310_at	34.83	13.70	2.54	-	EIF2C2	Eukaryotic translation initiation factor 2C, 2
200074_s_at	11.82	4.67	2.53	+	RPL14	ribosomal protein L14
200869_at	29.52	11.75	2.51	+	RPL18A	ribosomal protein L18a
218270_at	7.18	2.92	2.46	+	MRPL24	mitochondrial ribosomal protein L24
209609_s_at	10.14	4.22	2.40	-	MRPL9	mitochondrial ribosomal protein L9
201254_x_at	2.75	1.19	2.31	+	RPS6	ribosomal protein S6
201154_x_at	5.49	2.40	2.29	+	RPL4	ribosomal protein L4
200010_at	5.97	2.63	2.27	+	RPL11	Ribosomal protein L11
201064_s_at	7.61	3.38	2.25	+	PABPC4	poly(A) binding protein, cytoplasmic 4 (inducible form)
200022_at	8.61	3.89	2.21	+	RPL18	ribosomal protein L18
212450_at	10.26	4.66	2.20	-	KIAA0256	KIAA0256 gene product
213414_s_at	3.95	1.83	2.16	+	RPS19	ribosomal protein S19
221798_x_at	0.88	0.41	2.16	-	RPS2	Ribosomal protein S2
211937_at	8.65	4.05	2.14	+	EIF4B	eukaryotic translation initiation factor 4B
208264_s_at	8.58	4.08	2.10	-	EIF3S1	eukaryotic translation initiation factor 3, subunit 1 alpha, 35kDa
200012_x_at	8.42	4.04	2.08	+	RPL21	ribosomal protein L21
200858_s_at	5.06	2.44	2.07	+	RPS8	ribosomal protein S8
209134_s_at	3.91	1.95	2.01	+	RPS6	ribosomal protein S6
208695_s_at	0.96	0.49	1.97	-	RPL39	ribosomal protein L39

DNA replication

219105_x_at	18.23	5.57	3.27	-	ORC6L	origin recognition complex, subunit 6 homolog-like
201890_at	37.16	11.68	3.18	-	RRM2	ribonucleotide reductase M2 polypeptide
211577_s_at	20.37	7.88	2.58	+	IGF1	insulin-like growth factor 1 (somatomedin C)
221521_s_at	44.39	17.27	2.57	-	Pfs2	DNA replication complex GINS protein PSF2
209773_s_at	17.73	7.37	2.40	-	RRM2	ribonucleotide reductase M2 polypeptide
209540_at	27.99	12.37	2.26	+	IGF1	insulin-like growth factor 1 (somatomedin C)
213033_s_at	24.87	11.15	2.23	+	NFIB	Nuclear factor I/B
213734_at	5.51	2.52	2.18	-	WSB2	WD repeat and SOCS box-containing 2
204767_s_at	7.16	3.28	2.18	-	FEN1	flap structure-specific endonuclease 1
204127_at	3.68	1.82	2.02	-	RFC3	replication factor C (activator 1) 3, 38kDa
208752_x_at	1.16	0.59	1.97	+	NAP1L1	nucleosome assembly protein 1-like 1
**Oncogenesis**						
208079_s_at	83.78	19.84	4.22	-	STK6	serine/threonine kinase 6
204092_s_at	43.30	11.83	3.66	-	STK6	serine/threonine kinase 6
213829_x_at	6.41	2.42	2.65	-	TNFRSF6B	tumor necrosis factor receptor superfamily, member 6b, decoy
206413_s_at	36.36	14.96	2.43	-	TCL1B	T-cell leukemia/lymphoma 1B
203035_s_at	7.62	3.14	2.42	-	PIAS3	protein inhibitor of activated STAT, 3
202095_s_at	51.32	21.44	2.39	-	BIRC5	baculoviral IAP repeat-containing 5 (survivin)
210434_x_at	3.61	1.54	2.34	-	JTB	jumping translocation breakpoint
209054_s_at	3.75	1.81	2.08	-	WHSC1	Wolf-Hirschhorn syndrome candidate 1
200048_s_at	2.32	1.14	2.04	-	JTB	jumping translocation breakpoint
203554_x_at	9.16	4.61	1.98	-	PTTG1	pituitary tumor-transforming 1
203192_at	5.92	3.01	1.97	-	ABCB6	ATP-binding cassette, sub-family B (MDR/TAP), member 6
**Metabolism**						
212070_at	41.12	14.17	2.90	-	GPR56	G protein-coupled receptor 56
221256_s_at	21.39	7.39	2.89	+	HDHD3	haloacid dehalogenase-like hydrolase domain containing 3
203067_at	13.34	4.66	2.86	-	PDHX	pyruvate dehydrogenase complex, component X
212062_at	35.52	12.70	2.80	-	ATP9A	ATPase, Class II, type 9A
202651_at	17.67	6.42	2.75	-	LPGAT1	lysophosphatidylglycerol acyltransferase 1
220892_s_at	25.32	9.50	2.67	+	PSAT1	phosphoserine aminotransferase 1

206335_at	9.17	3.62	2.53	-	GALNS	galactosamine (N-acetyl)-6-sulfate sulfatase
202722_s_at	16.76	6.66	2.51	-	GFPT1	glutamine-fructose-6-phosphate transaminase 1
212353_at	45.42	18.09	2.51	-	SULF1	sulfatase 1
221928_at	39.21	16.23	2.42	+	ACACB	acetyl-Coenzyme A carboxylase beta
219616_at	10.26	4.30	2.39	-	FLJ21963	FLJ21963 protein
202464_s_at	48.50	20.47	2.37	-	PFKFB3	6-phosphofructo-2-kinase/fructose-2,6-biphosphatase 3
59705_at	9.15	3.93	2.33	-	SCLY	selenocysteine lyase
217776_at	21.38	9.75	2.19	-	RDH11	retinol dehydrogenase 11
218025_s_at	9.02	4.32	2.09	+	PECI	peroxisomal D3,D2-enoyl-CoA isomerase
209935_at	12.20	5.92	2.06	-	ATP2C1	ATPase, Ca++ transporting, type 2C, member 1
200824_at	31.66	15.69	2.02	+	GSTP1	glutathione S-transferase pi
201626_at	4.32	2.15	2.01	-	INSIG1	insulin induced gene 1
**Cellular defense response**						
215633_x_at	13.89	3.94	3.52	+	LST1	leukocyte specific transcript 1
210629_x_at	5.76	1.66	3.47	+	LST1	leukocyte specific transcript 1
206983_at	12.57	3.83	3.28	+	CCR6	chemokine (C-C motif) receptor 6
211582_x_at	13.68	4.48	3.06	+	LST1	leukocyte specific transcript 1
211581_x_at	9.43	3.52	2.68	+	LST1	leukocyte specific transcript 1
210116_at	21.00	8.06	2.61	+	SH2D1A	SH2 domain protein 1A, Duncan's disease
211529_x_at	6.25	2.59	2.41	+	HLA-G	HLA-G histocompatibility antigen, class I, G
210514_x_at	9.37	4.20	2.23	+	HLA-G	HLA-G histocompatibility antigen, class I, G
211528_x_at	4.96	2.35	2.11	+	HLA-G	HLA-G histocompatibility antigen, class I, G
207008_at	12.62	6.08	2.08	+	IL8RB	interleukin 8 receptor, beta
206978_at	4.21	2.05	2.05	+	CCR2	chemokine (C-C motif) receptor 2
211567_at	10.37	5.27	1.97	+	---	---
205495_s_at	7.10	3.63	1.96	+	GNLY	granulysin
**Chemotaxis**						
206983_at	15.76	4.80	3.28	+	CCR6	chemokine (C-C motif) receptor 6
210072_at	30.51	10.90	2.80	+	CCL19	chemokine (C-C motif) ligand 19
207850_at	17.47	6.65	2.63	+	CXCL3	chemokine (C-X-C motif) ligand 3

216598_s_at	28.42	11.20	2.54	+	CCL2	chemokine (C-C motif) ligand 2
214435_x_at	4.34	1.82	2.39	-	RALA	v-ral simian leukemia viral oncogene homolog A (ras related)
204470_at	7.69	3.23	2.38	+	CXCL1	chemokine (C-X-C motif) ligand 1
209687_at	13.77	5.85	2.35	+	CXCL12	chemokine (C-X-C motif) ligand 12 (stromal cell-derived factor 1)
203666_at	5.37	2.38	2.26	+	CXCL12	chemokine (C-X-C motif) ligand 12 (stromal cell-derived factor 1)
207008_at	15.81	7.61	2.08	+	IL8RB	interleukin 8 receptor, beta
209201_x_at	9.29	4.50	2.06	+	CXCR4	chemokine (C-X-C motif) receptor 4
206978_at	5.28	2.57	2.05	+	CCR2	chemokine (C-C motif) receptor 2
206337_at	6.09	3.06	1.99	+	CCR7	chemokine (C-C motif) receptor 7
211567_at	13.00	6.60	1.97	+	---	---
214974_x_at	8.80	4.49	1.96	+	CXCL5	chemokine (C-X-C motif) ligand 5
