# Supplementary material for: Pathway analysis of gene signatures predicting metastasis of node-negative primary breast cancer
Source: BMC Cancer. 2007 Sep 25;7:182. doi: 10.1186/1471-2407-7-182 (PMC2077336; doi:10.1186/1471-2407-7-182)
Supplement: Additional file 3 — Top 20 prognostic pathways in ER-negative tumors. The data provided represent the results of the Geneplot function in the Global test program. The contribution of each individual gene in the top 20 prognostic pathways with distant metastasis-free survival in ER-negative tumors is plotted. [file 1471-2407-7-182-S3.pdf]

Additional file 3: Top 20 prognostic pathways in ER-negative tumors

Association of the expression of individual genes with DMFS time for selected over-represented pathways. Geneplot function in the Global Test program [1, 2] was applied and the contribution of the individual genes in each selected pathway is plotted. The numbers at the X-axis represent the number of genes in the respective pathway in ER-negative tumors. The values at the Y-axis, represent the contribution (influence) of each individual gene in the selected pathway with DMFS. Negative values indicate there is no association between the gene expression and DMFS. Each thin horizontal line in a bar (influence) indicates one standard deviation away from the reference point, two or more horizontal lines in a bar indicates that the association of the corresponding gene with DMFS is statistically significant. The green bars reflect genes that are positively associated with DMFS, indicating a higher expression in tumors without metastatic capability. The red bars reflect genes that are negatively associated with DMFS, indicative of higher expression in tumors with metastatic capability. The individual positive and or negative contribution of the significant genes are presented in Additional file 4.

References

1. Goeman JJ, van de Geer SA, de Kort F, van Houwelingen HC: **A global test for groups of genes: testing association with a clinical outcome.** *Bioinformatics* 2004, **20:**93-99.
2. Goeman JJ, Oosting J, Cleton-Jansen AM, Anninga JK, van Houwelingen HC: **Testing association of a pathway with survival using gene expression data.** *Bioinformatics* 2005, **21:**1950-1957.

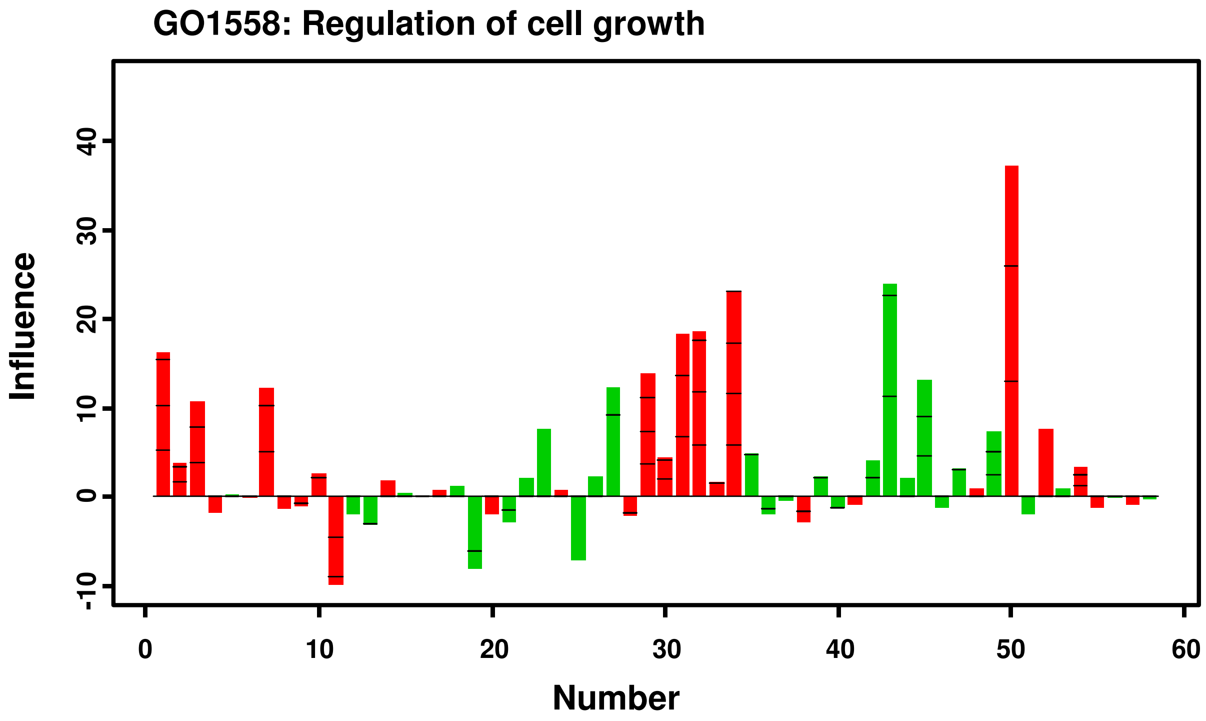

GO8277: Regulation of G-coupled receptor signaling

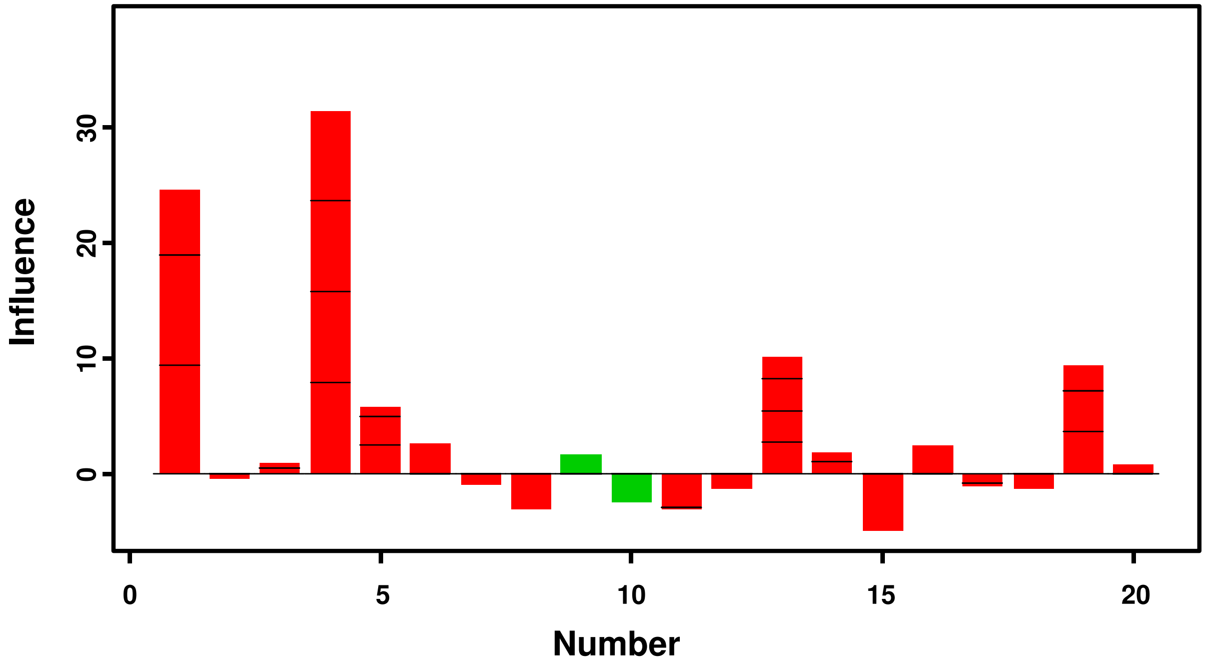

GO1501: Skeletal development

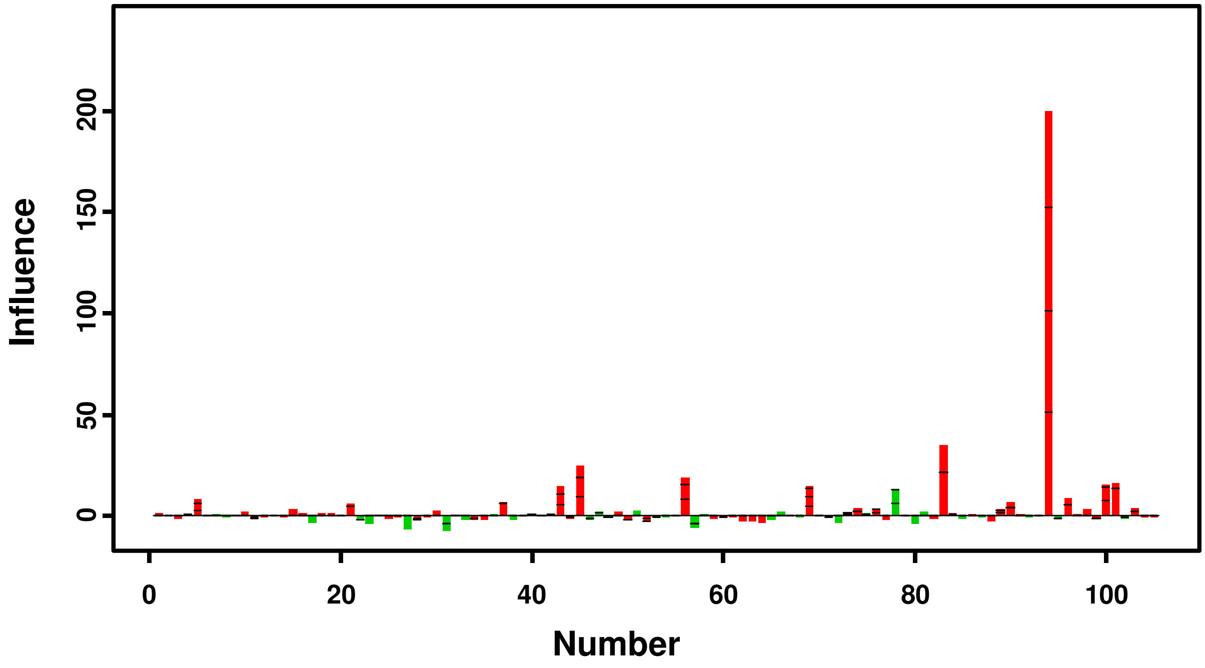

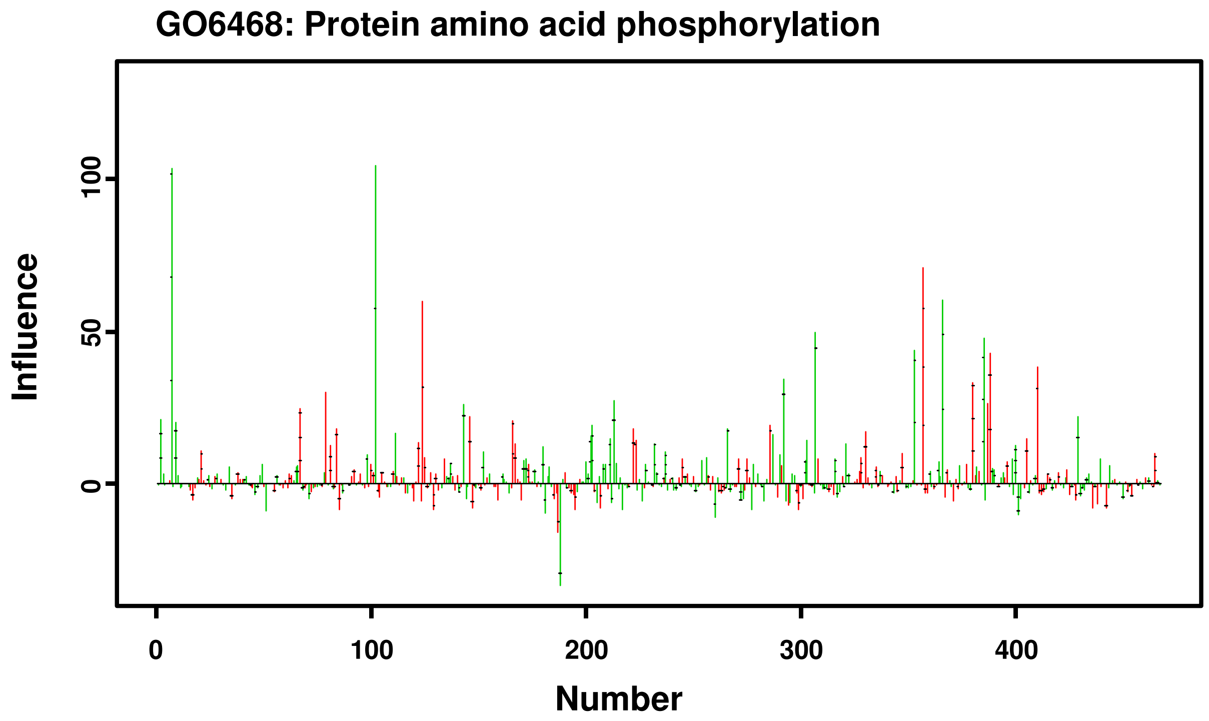

GO6468: Protein amino acid phosphorylation
Influence
100
50
0
0
100
200
300
400
Number

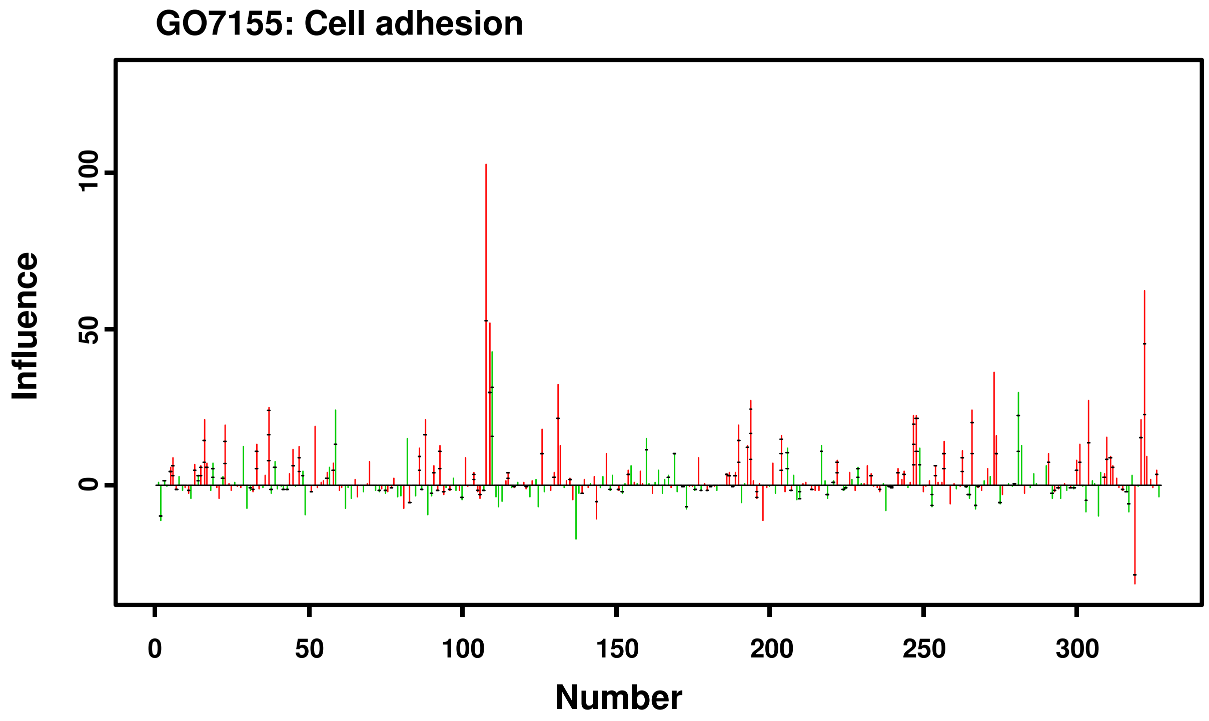

GO7155: Cell adhesion
Influence
100
50
0
0
50
100
150
200
250
300
Number

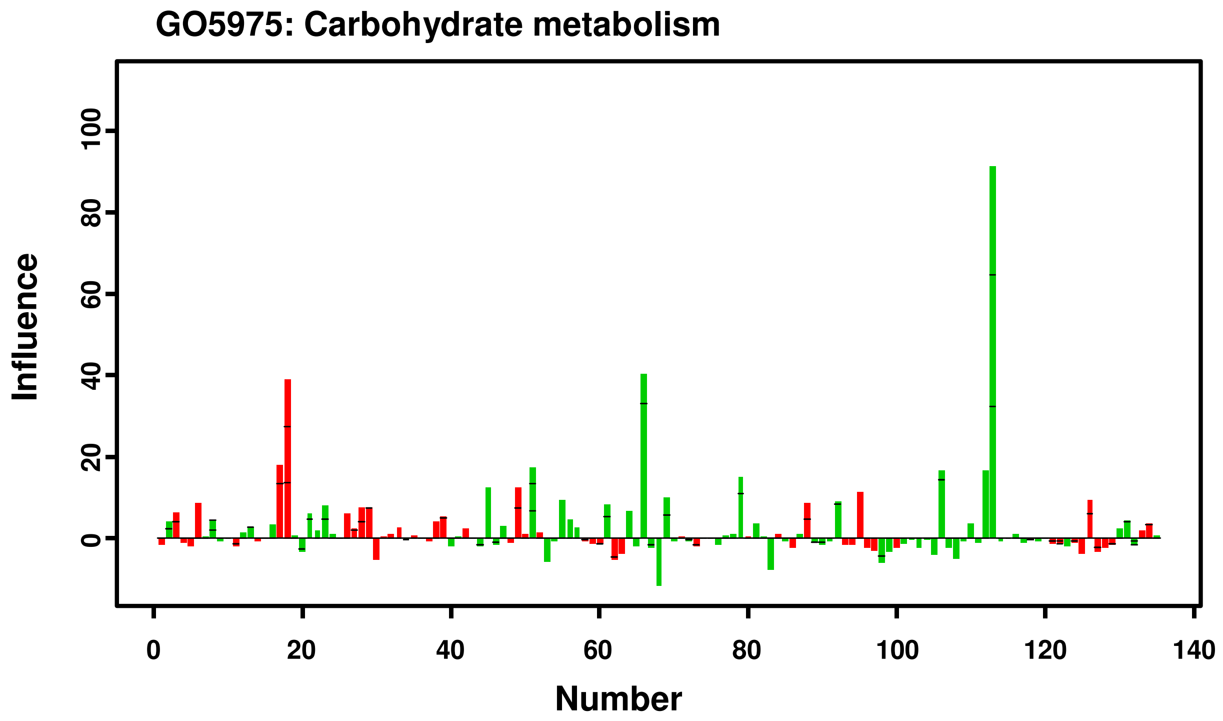
GO5975: Carbohydrate metabolism
Influence
0
20
40
60
80
100
0
20
40
60
80
100
120
140
Number

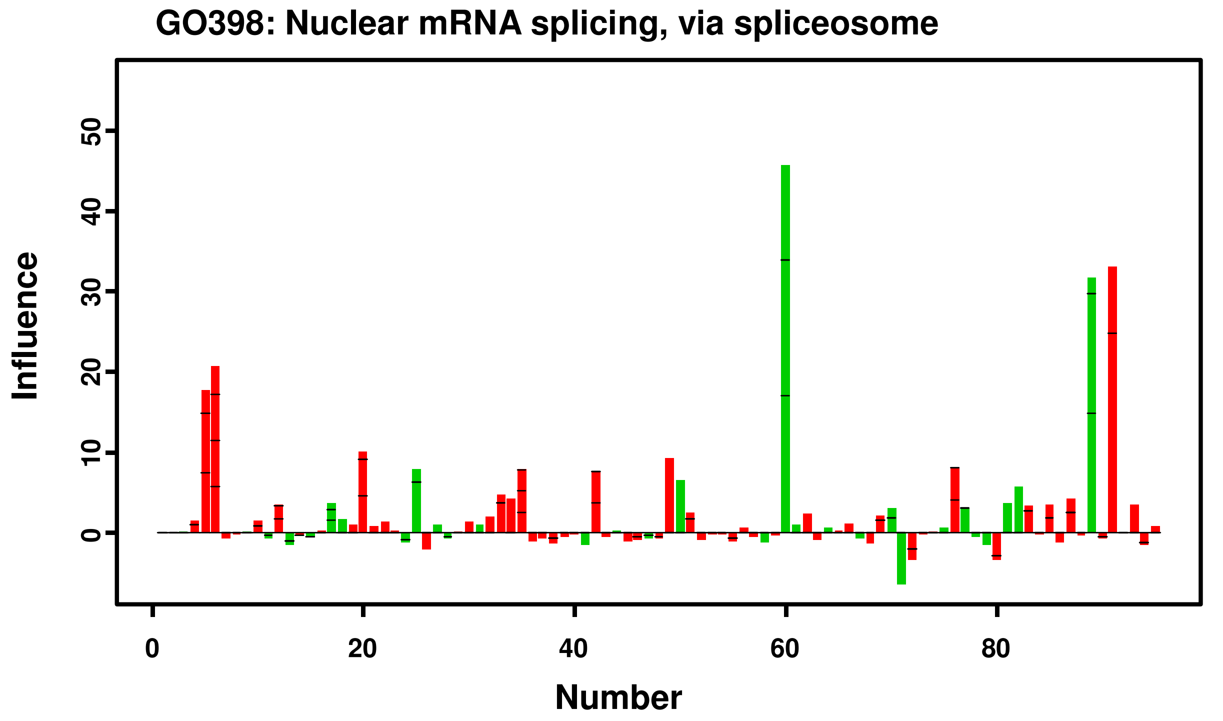
GO398: Nuclear mRNA splicing, via spliceosome
Influence
0
10
20
30
40
50
0
20
40
60
80
Number

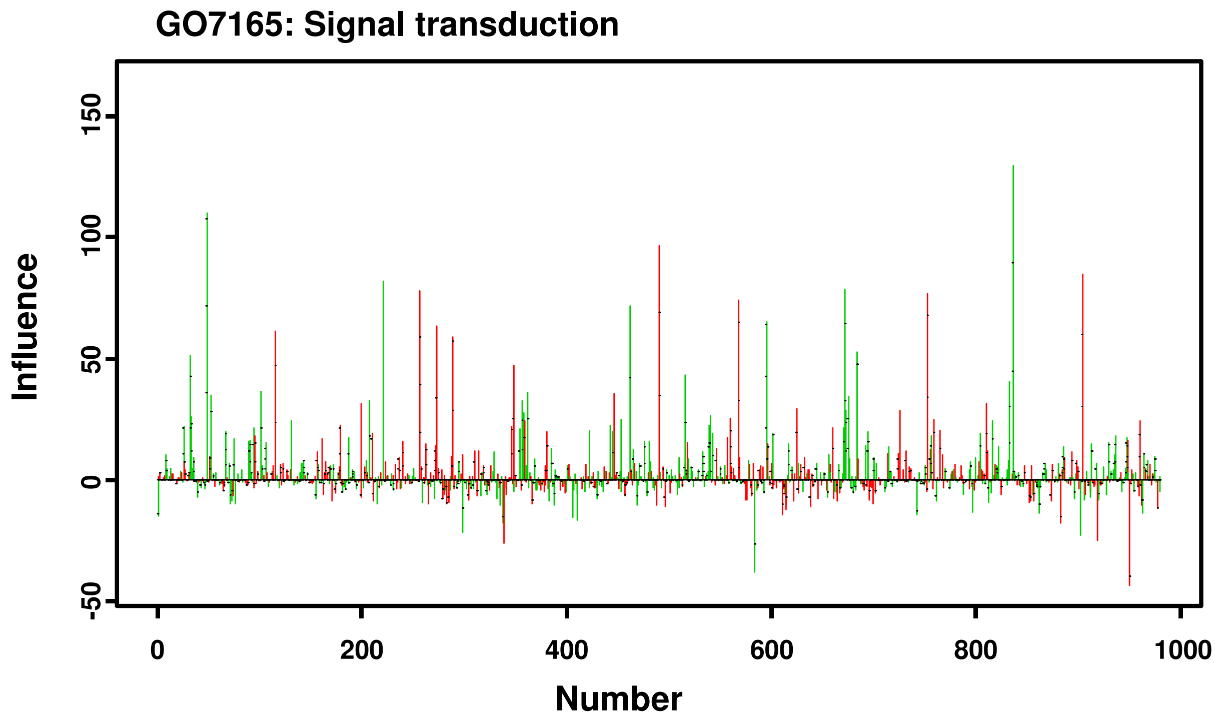
GO7165: Signal transduction
Influence
150
100
50
0
-50
0
200
400
600
800
1000
Number

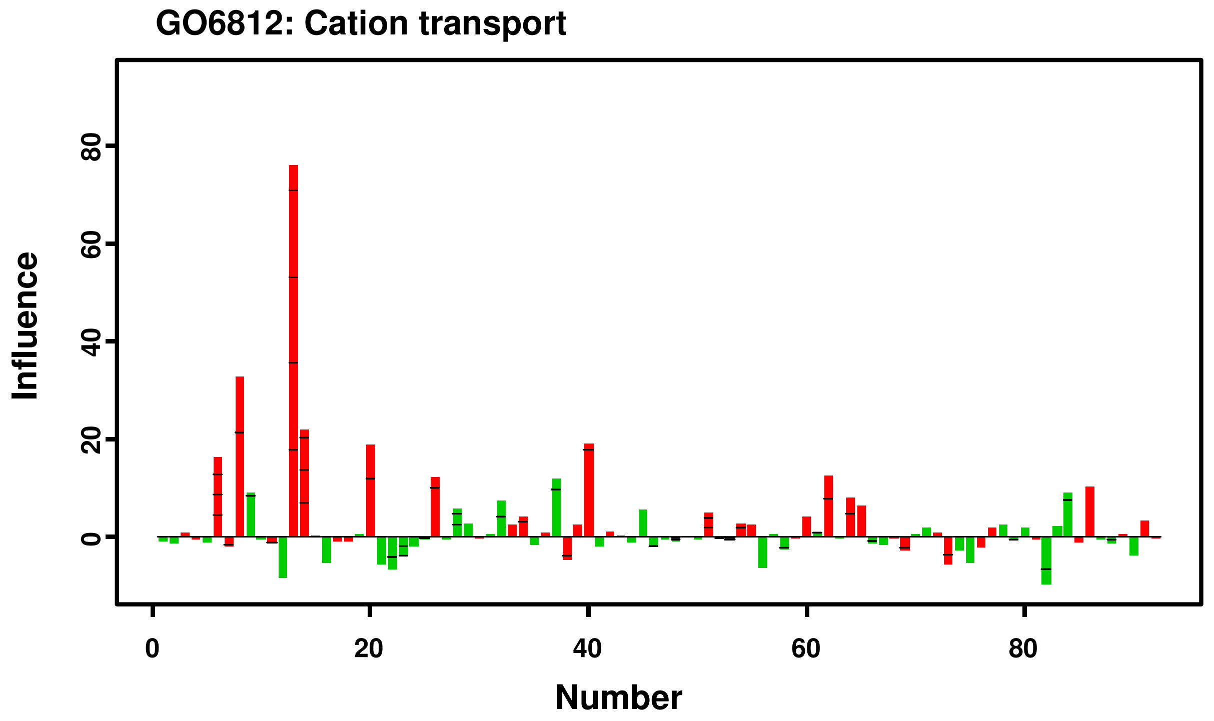
GO6812: Cation transport
Influence
80
60
40
20
0
0
20
40
60
80
Number

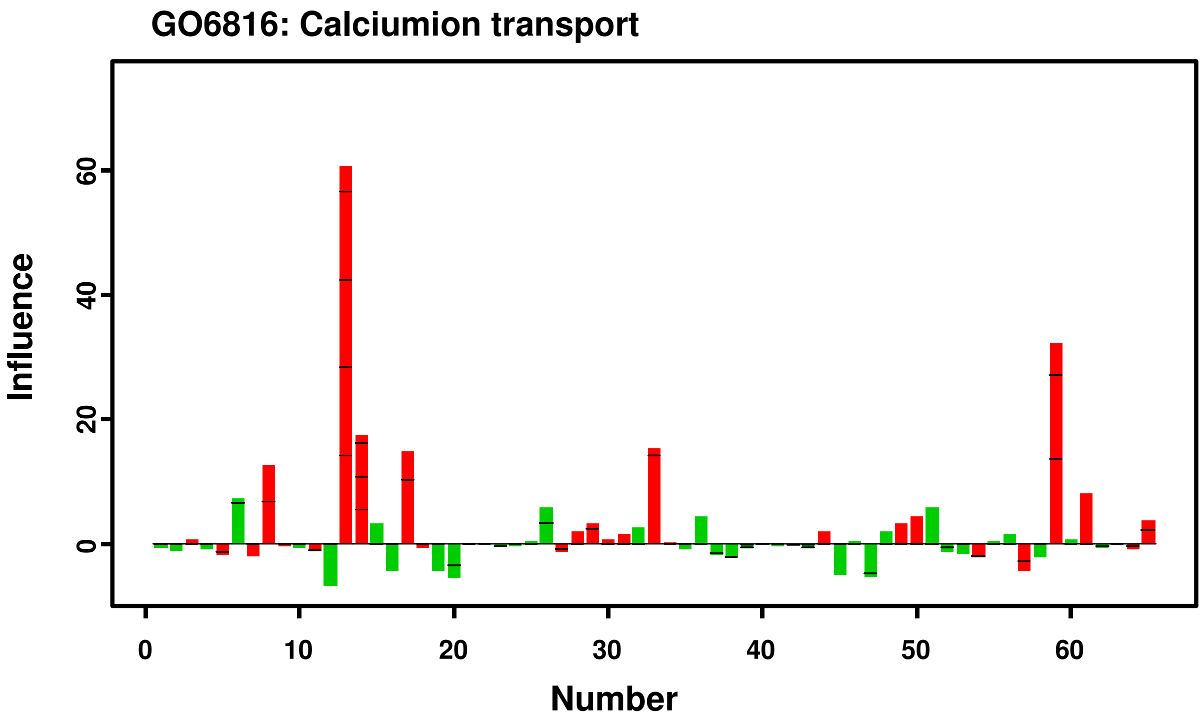
GO6816: Calciumion transport
Influence
Number
0
20
40
60
10
30
50

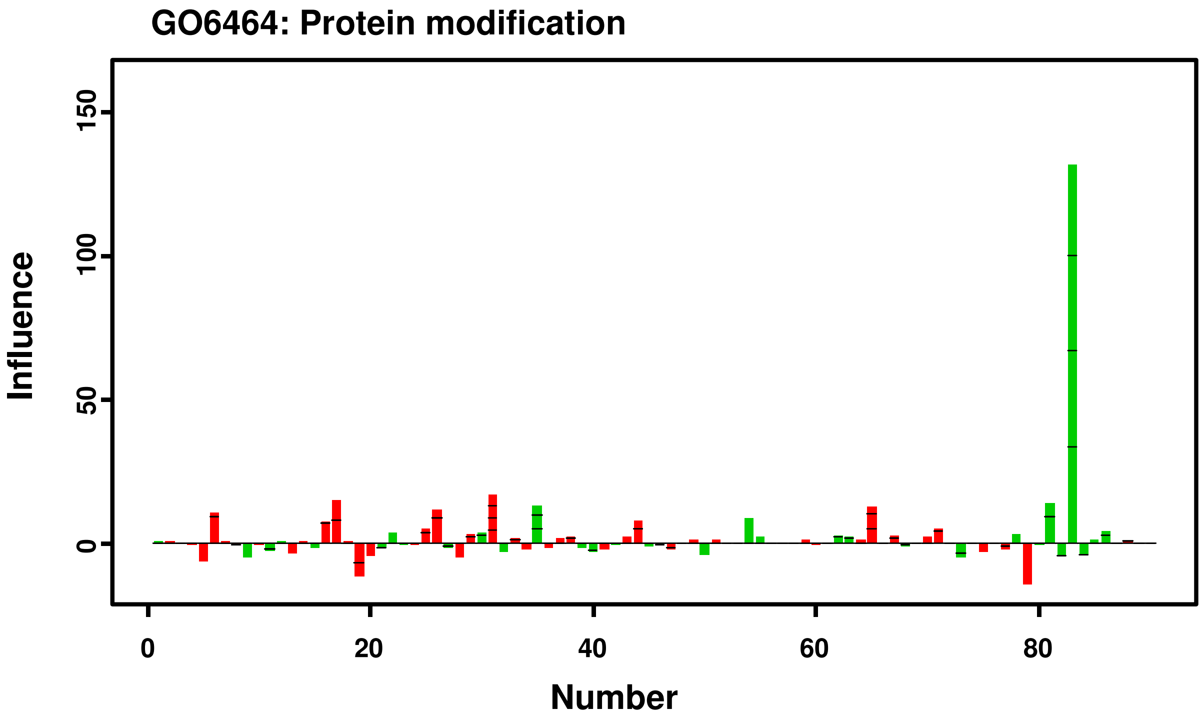
GO6464: Protein modification
Influence
Number
0
50
100
150
20
40
60
80

GO7242: Intracellular signaling cascade

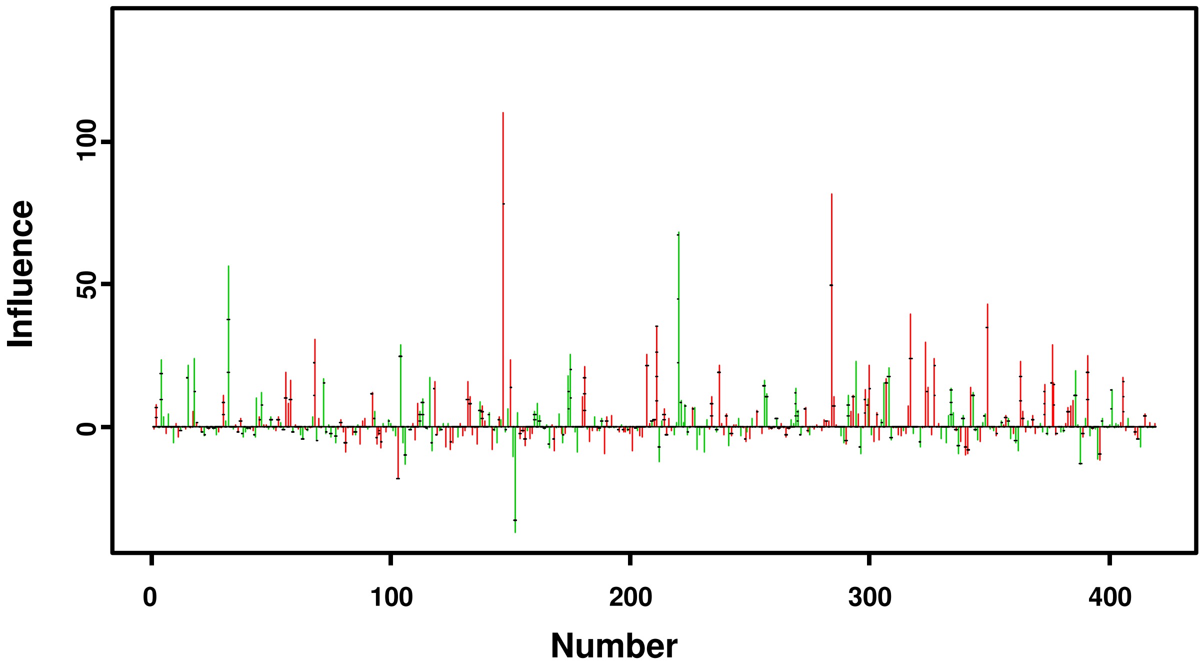

GO6397: mRNA processing

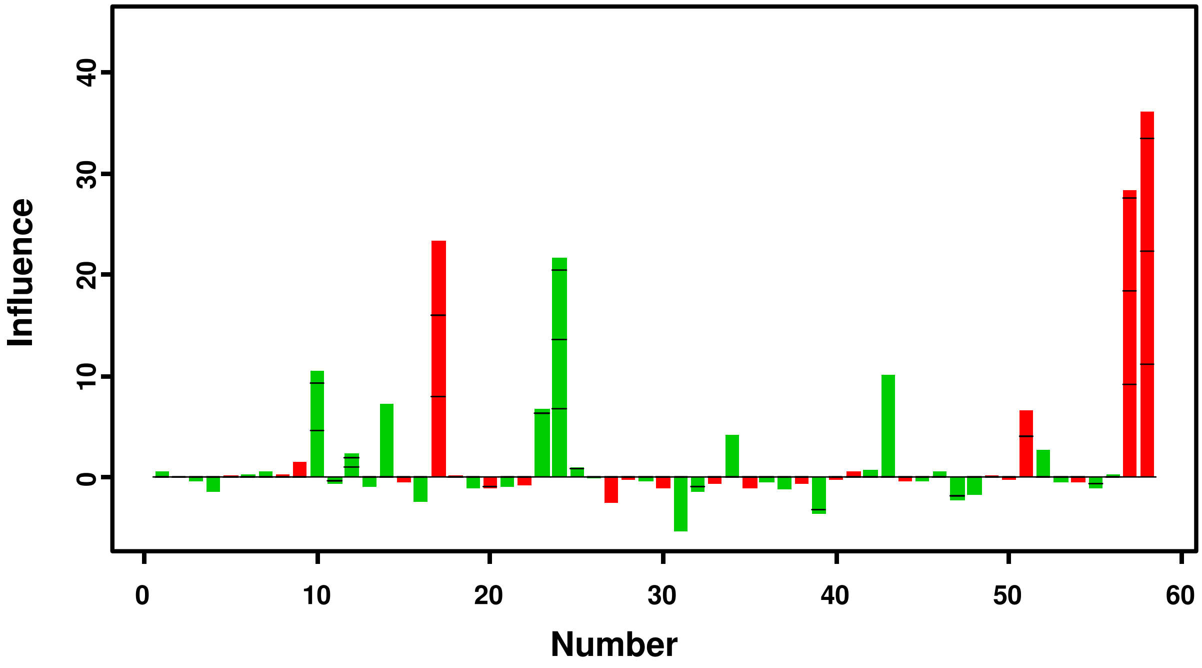

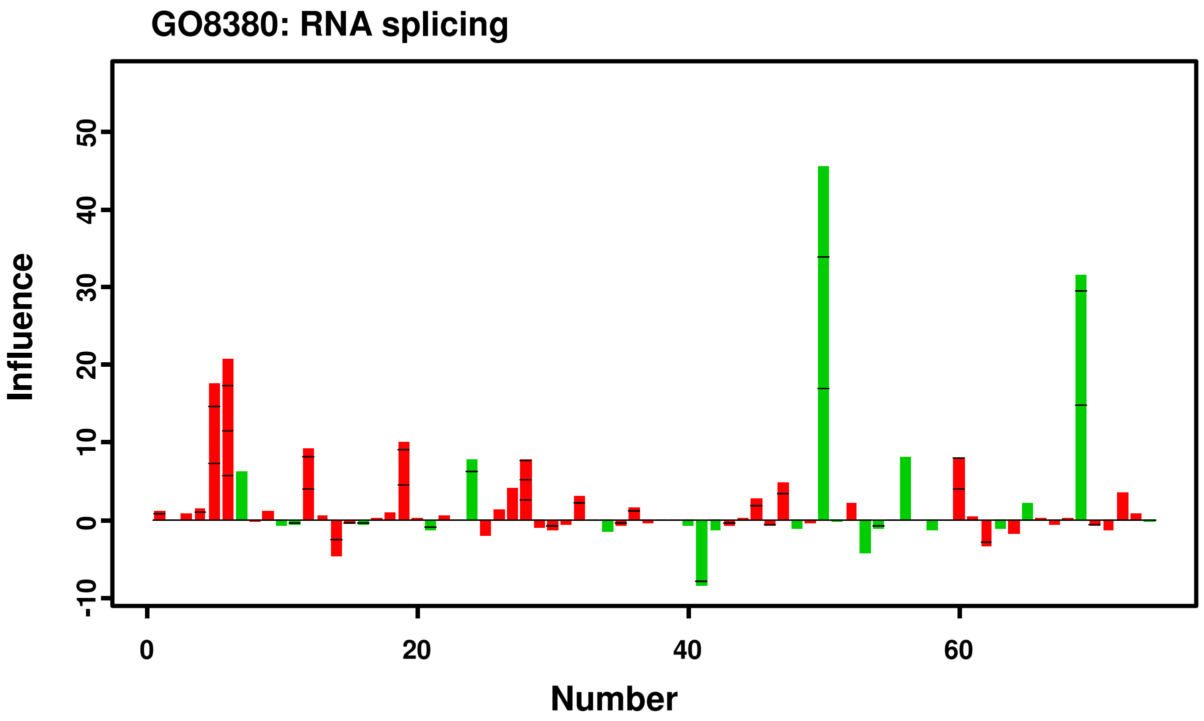
GO8380: RNA splicing
Influence
Number
-10
0
10
20
30
40
50
0
20
40
60

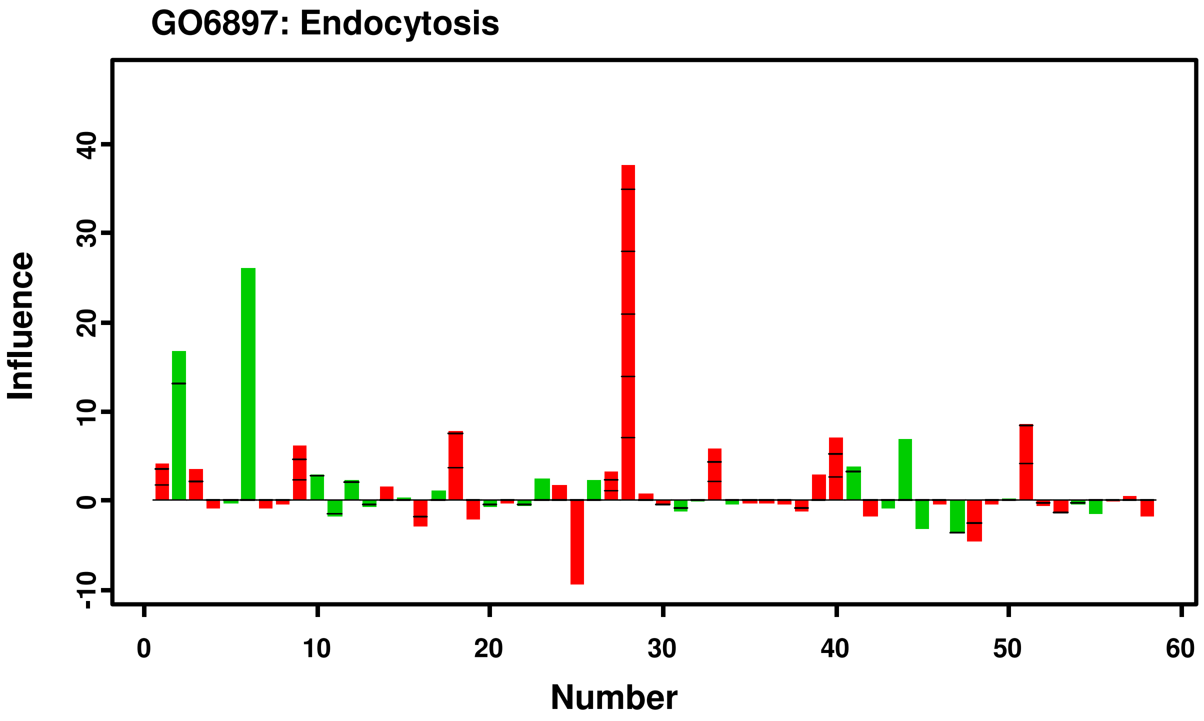
GO6897: Endocytosis
Influence
Number
-10
0
10
20
30
40
0
10
20
30
40
50
60

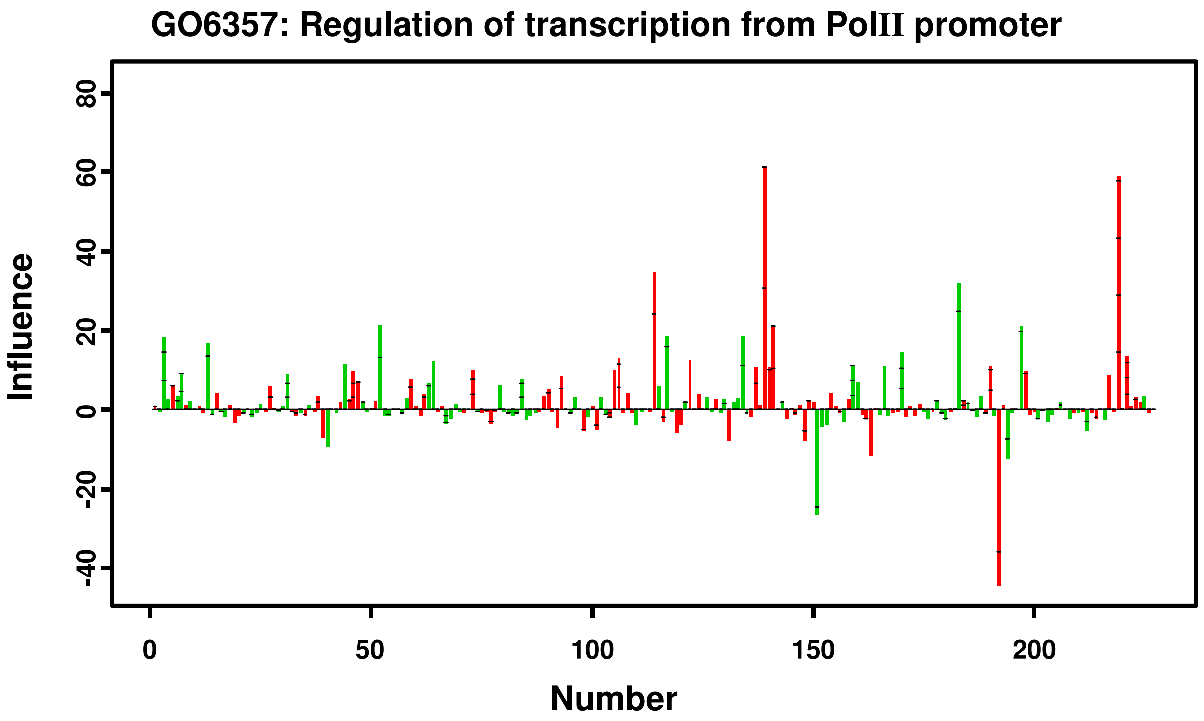

GO6357: Regulation of transcription from PolII promoter
Influence
Number
80
60
40
20
0
-20
-40
0
50
100
150
200

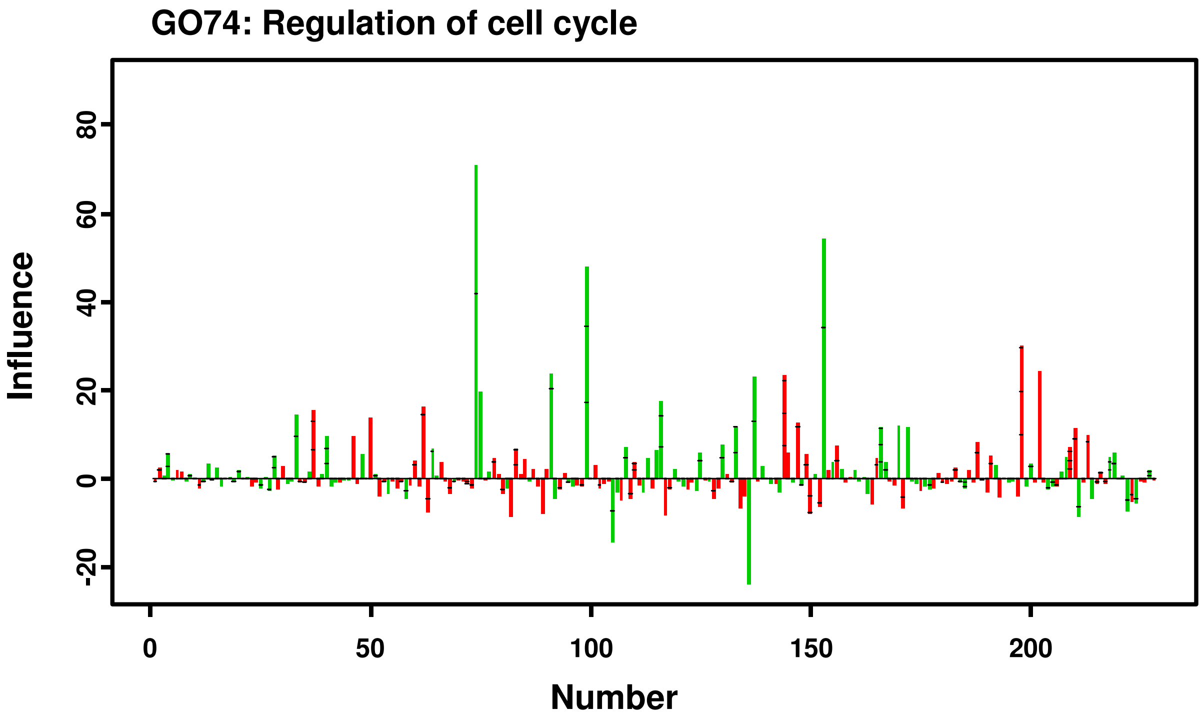

GO74: Regulation of cell cycle
Influence
Number
80
60
40
20
0
-20
0
50
100
150
200

GO6461: Protein complex assembly

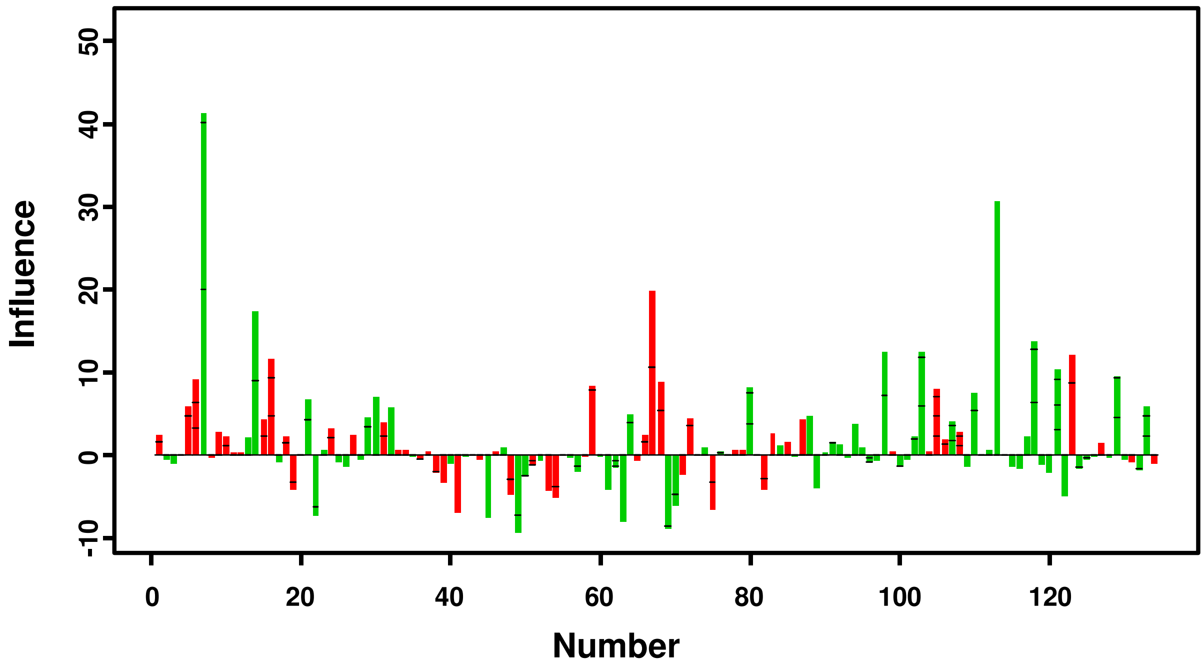

GO6412: Protein biosynthesis

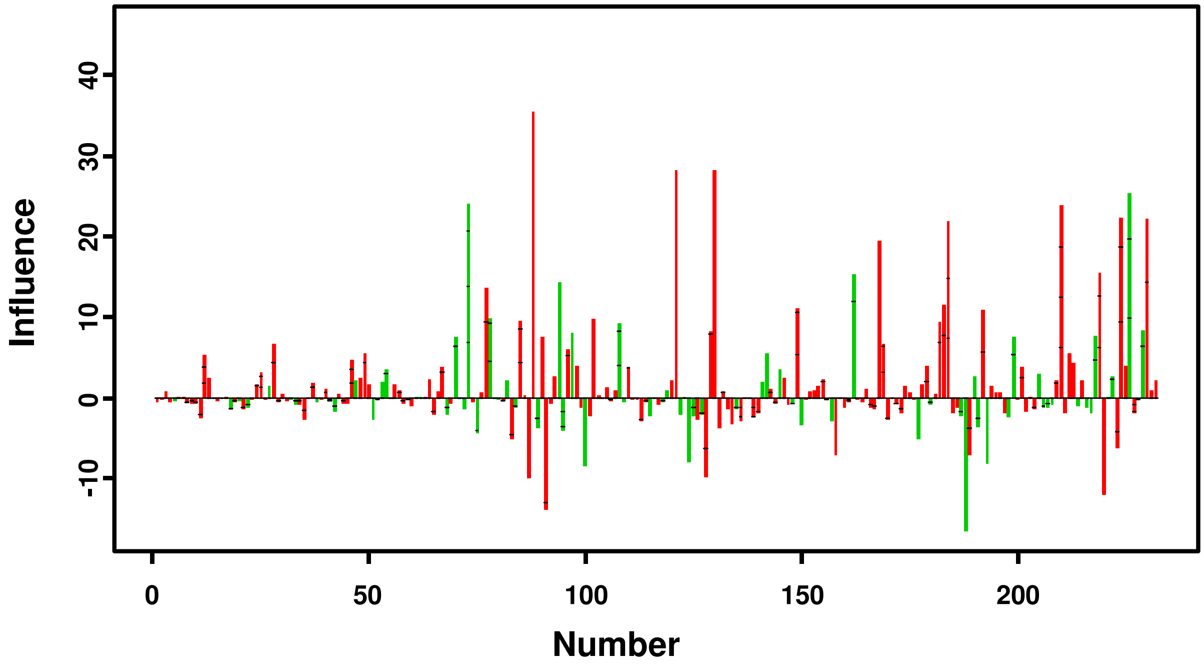

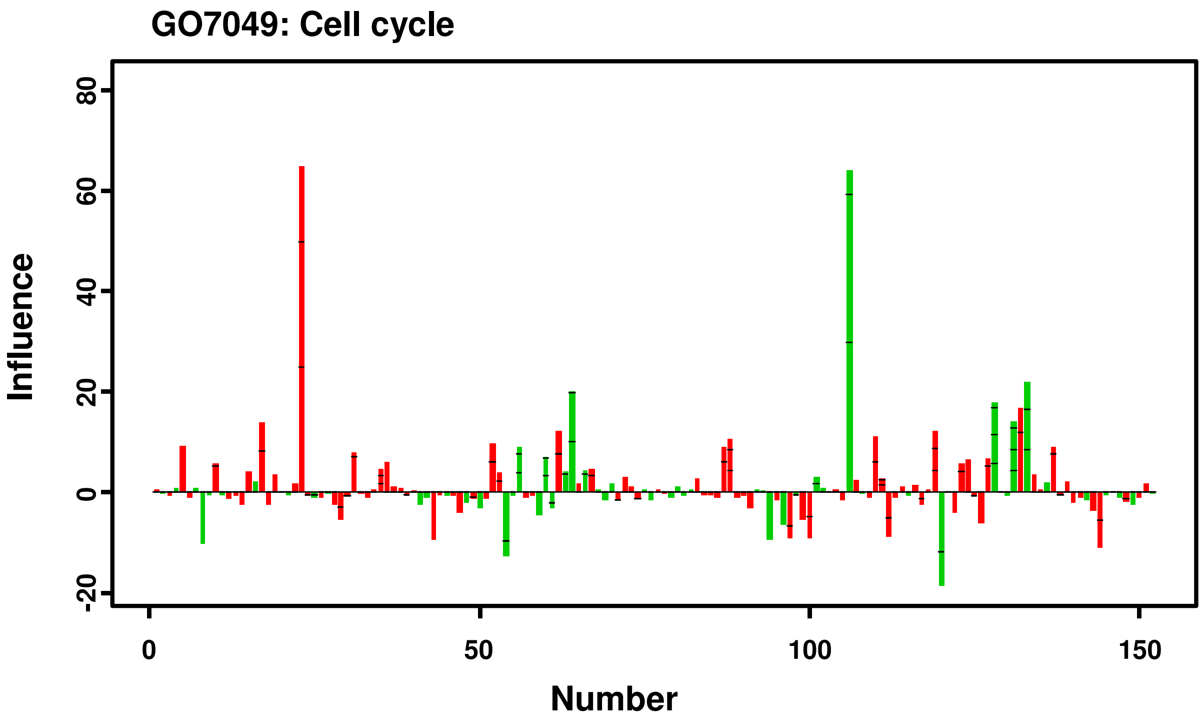
GO7049: Cell cycle
Influence
Number
80
60
40
20
0
-20
0
50
100
150
