# Supplementary material for: Pathway analysis of gene signatures predicting metastasis of node-negative primary breast cancer
Source: BMC Cancer. 2007 Sep 25;7:182. doi: 10.1186/1471-2407-7-182 (PMC2077336; doi:10.1186/1471-2407-7-182)
Supplement: Additional file 4 — Significant genes in the top 20 prognostic pathways for ER-negative tumors. The data provided represent the contribution, standard deviation, and z-scores of each individual gene with distant metastasis-free survival in ER-negative tumors in the top 20 prognostic pathways. [file 1471-2407-7-182-S4.pdf]

Additional file 4: Significant genes in the top 20 prognostic pathways for ER-negative tumors

Association of the expression of individual genes with distant metastasis-free survival (DMFS) time for selected over-represented pathways in ER-negative tumors was studied with the Geneplot function in the Global Test program [1, 2]. The contribution ("Influence") and standard deviation ("SD") of the significant genes (with "z-score" ≥1.96) of those presented in Additional file 3, are listed. The "+" sign in the "DMFS" column reflects a positive association with DMFS time, indicating a higher expression in tumors without metastatic capability. The "-" sign in the "DMFS" column reflects a negative association with distant metastasis-free survival time, indicative of higher expression in tumors with metastatic capability.

References

1. Goeman JJ, van de Geer SA, de Kort F, van Houwelingen HC: **A global test for groups of genes: testing association with a clinical outcome.** *Bioinformatics* 2004, **20:**93-99.
2. Goeman JJ, Oosting J, Cleton-Jansen AM, Anninga JK, van Houwelingen HC: **Testing association of a pathway with survival using gene expression data.** *Bioinformatics* 2005, **21:**1950-1957.

Probe set	Influence	SD	z-score	DMFS	Gene Symbol	Gene Title
**Regulation of cell growth**						
209648_x_at	23.16	5.77	4.01	-	SOCS5	suppressor of cytokine signaling 5
208127_s_at	13.90	3.71	3.75	-	SOCS5	suppressor of cytokine signaling 5
209550_at	18.66	5.88	3.18	-	NDN	necdin homolog (mouse)
201162_at	16.18	5.15	3.14	-	IGFBP7	insulin-like growth factor binding protein 7
212279_at	13.20	4.53	2.91	+	MAC30	hypothetical protein MAC30
213337_s_at	7.30	2.53	2.88	+	SOCS1	suppressor of cytokine signaling 1
213910_at	37.27	12.99	2.87	-	IGFBP7	insulin-like growth factor binding protein 7
217982_s_at	3.33	1.20	2.78	-	MORF4L1	mortality factor 4 like 1
201185_at	10.66	3.90	2.73	-	HTRA1	HtrA serine peptidase 1
209101_at	18.31	6.81	2.69	-	CTGF	connective tissue growth factor

202149_at	12.23	5.12	2.39	-	NEDD9	neural precursor cell expressed, developmentally down-regulated 9
201163_s_at	3.89	1.69	2.31	-	IGFBP7	insulin-like growth factor binding protein 7
208394_x_at	4.40	2.07	2.12	-	ESM1	endothelial cell-specific molecule 1
211513_s_at	23.97	11.32	2.12	+	OGFR	opioid growth factor receptor
211512_s_at	4.18	2.11	1.98	+	OGFR	opioid growth factor receptor
**Regulation of G-protein coupled receptor signaling pathway**						
204337_at	31.44	7.89	3.99	-	RGS4	regulator of G-protein signalling 4
209324_s_at	10.18	2.73	3.73	-	RGS16	regulator of G-protein signalling 16
220300_at	9.44	3.61	2.61	-	RGS3	regulator of G-protein signalling 3
202388_at	24.64	9.45	2.61	-	RGS2	regulator of G-protein signalling 2, 24kDa
204396_s_at	5.77	2.47	2.34	-	GRK5	G protein-coupled receptor kinase 5
**Skeletal development**						
217404_s_at	199.74	50.77	3.93	-	COL2A1	collagen, type II, alpha 1
210135_s_at	14.72	4.62	3.19	-	SHOX2	short stature homeobox 2
205941_s_at	14.81	5.41	2.74	-	COL10A1	collagen, type X, alpha 1
201792_at	8.36	3.08	2.72	-	AEBP1	AE binding protein 1
206091_at	25.05	9.62	2.60	-	MATN3	matrilin 3
208443_x_at	18.61	7.88	2.36	-	SHOX2	short stature homeobox 2
213943_at	3.30	1.48	2.23	-	TWIST1	twist homolog 1(Drosophila)
220076_at	15.77	7.23	2.18	-	ANKH	ankylosis, progressive homolog (mouse)
210427_x_at	1.45	0.69	2.10	-	ANXA2	annexin A2
210809_s_at	3.36	1.64	2.05	-	POSTN	periostin, osteoblast specific factor
210973_s_at	12.86	6.33	2.03	+	FGFR1	fibroblast growth factor receptor 1
213503_x_at	1.24	0.64	1.96	-	ANXA2	annexin A2
**Protein amino acid phosphorylation**						
213595_s_at	70.67	19.13	3.69	-	CDC42BPA	CDC42 binding protein kinase alpha (DMPK-like)
215050_x_at	47.49	13.74	3.46	+	MAPKAPK2	mitogen-activated protein kinase-activated protein kinase 2
208875_s_at	10.32	3.05	3.39	+	PAK2	p21 (CDKN1A)-activated kinase 2
216711_s_at	12.50	3.71	3.37	+	TAF1	TAF1 RNA polymerase II, TATA box binding protein (TBP)-associated factor

203131_at	24.32	7.64	3.18	-	PDGFRA	platelet-derived growth factor receptor, alpha polypeptide
214683_s_at	32.74	10.72	3.05	-	CLK1	CDC-like kinase 1
201401_s_at	103.31	33.85	3.05	+	ADRBK1	adrenergic, beta, receptor kinase 1
203552_at	12.54	4.52	2.77	-	MAP4K5	mitogen-activated protein kinase kinase kinase kinase 5
205880_at	6.18	2.31	2.68	-	PRKD1	protein kinase D1
200604_s_at	20.81	8.27	2.52	+	PRKAR1A	protein kinase, cAMP-dependent, regulatory, type I, alpha
207239_s_at	19.06	7.73	2.47	+	PCTK1	PCTAIRE protein kinase 1
214007_s_at	60.27	24.46	2.46	+	PTK9	PTK9 protein tyrosine kinase 9
212530_at	8.39	3.43	2.45	-	NEK7	NIMA (never in mitosis gene a)-related kinase 7
212740_at	5.21	2.15	2.43	-	PIK3R4	phosphoinositide-3-kinase, regulatory subunit 4, p150
215296_at	42.64	17.82	2.39	-	CDC42BPA	CDC42 binding protein kinase alpha (DMPK-like)
201461_s_at	20.08	8.57	2.34	+	MAPKAPK2	mitogen-activated protein kinase-activated protein kinase 2
204396_s_at	13.51	5.78	2.34	-	GRK5	G protein-coupled receptor kinase 5
207667_s_at	14.58	6.35	2.30	+	MAP2K3	mitogen-activated protein kinase kinase 3
202127_at	10.85	4.86	2.23	-	PRPF4B	PRP4 pre-mRNA processing factor 4 homolog B (yeast)
59644_at	9.95	4.50	2.21	-	BMP2K	BMP2 inducible kinase
207228_at	15.38	6.96	2.21	+	PRKACG	protein kinase, cAMP-dependent, catalytic, gamma
213490_s_at	43.56	20.23	2.15	+	MAP2K2	mitogen-activated protein kinase kinase 2
211599_x_at	8.19	3.83	2.14	+	MET	met proto-oncogene (hepatocyte growth factor receptor)
211208_s_at	7.35	3.44	2.14	+	CASK	calcium/calmodulin-dependent serine protein kinase (MAGUK family)
205578_at	20.67	9.69	2.13	-	ROR2	receptor tyrosine kinase-like orphan receptor 2
204813_at	6.64	3.30	2.01	+	MAPK10	mitogen-activated protein kinase 10
208824_x_at	12.76	6.35	2.01	+	PCTK1	PCTAIRE protein kinase 1
**Cell adhesion**						
212724_at	22.05	6.48	3.40	-	RND3	Rho family GTPase 3
209210_s_at	26.72	8.13	3.28	-	PLEKHC1	pleckstrin homology domain containing, family C member 1
202363_at	24.96	7.95	3.14	-	SPOCK	sparc/osteonectin, cwcv and kazal-like domains proteoglycan (testican)
209651_at	15.39	4.94	3.12	-	TGFB1I1	transforming growth factor beta 1 induced transcript 1
201505_at	21.00	7.24	2.90	-	LAMB1	laminin, beta 1
200771_at	8.56	3.01	2.84	-	LAMC1	laminin, gamma 1 (formerly LAMB2)
213790_at	14.02	4.96	2.83	-	ADAM12	ADAM metallopeptidase domain 12 (meltrin alpha)

203083_at	12.25	4.39	2.79	-	THBS2	thrombospondin 2
222020_s_at	62.24	22.64	2.75	-	HNT	neurotrimin
205532_s_at	42.40	15.54	2.73	+	CDH6	cadherin 6, type 2, K-cadherin (fetal kidney)
201792_at	18.97	6.98	2.72	-	AEBP1	AE binding protein 1
209101_at	19.18	7.13	2.69	-	CTGF	connective tissue growth factor
215904_at	29.42	11.01	2.67	+	MLLT4	myeloid/lymphoid or mixed-lineage leukemia (trithorax homolog, Drosophila); translocated to, 4
201561_s_at	6.71	2.62	2.56	+	CLSTN1	calsyntenin 1
204677_at	11.48	4.53	2.53	-	CDH5	cadherin 5, type 2, VE-cadherin (vascular epithelium)
214212_x_at	10.68	4.26	2.51	-	PLEKHC1	pleckstrin homology domain containing, family C (with FERM domain) member 1
214375_at	23.91	10.02	2.39	-	PPFIBP1	PTPRF interacting protein, binding protein 1 (liprin beta 1)
202149_at	12.81	5.37	2.39	-	NEDD9	neural precursor cell expressed, developmentally down-regulated 9
204955_at	12.74	5.34	2.39	-	SRPX	sushi-repeat-containing protein, X-linked
209873_s_at	11.75	5.14	2.29	+	PKP3	plakophilin 3
211208_s_at	5.66	2.65	2.14	+	CASK	calcium/calmodulin-dependent serine protein kinase (MAGUK family)
205176_s_at	3.87	1.82	2.13	-	ITGB3BP	integrin beta 3 binding protein (beta3-endonexin)
201281_at	2.86	1.39	2.06	+	ADRM1	adhesion regulating molecule 1
212843_at	22.00	10.69	2.06	-	NCAM1	neural cell adhesion molecule 1
210809_s_at	7.63	3.72	2.05	-	POSTN	periostin, osteoblast specific factor
205656_at	4.03	1.96	2.05	-	PCDH17	protocadherin 17
201438_at	5.86	2.89	2.03	-	COL6A3	collagen, type VI, alpha 3
213241_at	6.19	3.06	2.02	-	PLXNC1	plexin C1
218975_at	26.96	13.55	1.99	-	COL5A3	collagen, type V, alpha 3
**Carbohydrate metabolism**						
202499_s_at	39.16	13.68	2.86	-	SLC2A3	solute carrier family 2 (facilitated glucose transporter), member 3
216010_x_at	91.48	32.31	2.83	+	FUT3	fucosyltransferase 3
205799_s_at	17.32	6.72	2.58	+	SLC3A1	solute carrier family 3, member 1
201765_s_at	4.24	2.08	2.04	+	HEXA	hexosaminidase A (alpha polypeptide)
**Nuclear mRNA splicing, via splicesome**						
200686_s_at	20.80	5.76	3.61	-	SFRS11	splicing factor, arginine/serine-rich 11
203376_at	7.88	2.58	3.06	-	CDC40	cell division cycle 40 homolog (yeast)

209162_s_at	45.77	16.98	2.69	+	PRPF4	PRP4 pre-mRNA processing factor 4 homolog (yeast)
201698_s_at	3.64	1.44	2.52	+	SFRS9	splicing factor, arginine/serine-rich 9
200685_at	17.74	7.38	2.40	-	SFRS11	splicing factor, arginine/serine-rich 11
202127_at	10.16	4.55	2.23	-	PRPF4B	PRP4 pre-mRNA processing factor 4 homolog B (yeast)
221546_at	31.79	14.83	2.14	+	PRPF18	PRP18 pre-mRNA processing factor 18 homolog (yeast)
201385_at	3.45	1.66	2.08	-	DHX15	DEAH (Asp-Glu-Ala-His) box polypeptide 15
204064_at	7.66	3.76	2.04	-	THOC1	THO complex 1
214016_s_at	8.09	4.04	2.00	-	SFPQ	Splicing factor proline/glutamine-rich
219119_at	3.44	1.75	1.97	-	LSM8	LSM8 homolog, U6 small nuclear RNA associated
**Signal transduction**						
204337_at	77.97	19.56	3.99	-	RGS4	regulator of G-protein signalling 4
209324_s_at	25.24	6.77	3.73	-	RGS16	regulator of G-protein signalling 16
204464_s_at	14.07	3.89	3.62	-	EDNRA	endothelin receptor type A
202247_s_at	14.76	4.24	3.48	+	MTA1	metastasis associated 1
221773_at	16.08	4.70	3.42	-	ELK3	ELK3, ETS-domain protein (SRF accessory protein 2)
203328_x_at	3.87	1.13	3.41	+	IDE	insulin-degrading enzyme
208875_s_at	10.94	3.23	3.39	+	PAK2	p21 (CDKN1A)-activated kinase 2
201835_s_at	19.43	6.22	3.12	+	PRKAB1	protein kinase, AMP-activated, beta 1 non-catalytic subunit
217496_s_at	6.53	2.13	3.07	+	IDE	insulin-degrading enzyme
209895_at	64.80	21.23	3.05	+	PTPN11	protein tyrosine phosphatase, non-receptor type 11
201401_s_at	109.49	35.88	3.05	+	ADRBK1	adrenergic, beta, receptor kinase 1
202716_at	7.60	2.50	3.05	+	PTPN1	protein tyrosine phosphatase, non-receptor type 1
215984_s_at	129.29	44.77	2.89	+	ARFRP1	ADP-ribosylation factor related protein 1
219837_s_at	84.68	29.97	2.83	-	CYTL1	cytokine-like 1
207987_s_at	96.20	34.37	2.80	-	GNRH1	gonadotropin-releasing hormone 1
204115_at	15.78	5.64	2.80	-	GNG11	guanine nucleotide binding protein (G protein), gamma 11
218157_x_at	13.07	4.70	2.78	+	CDC42SE1	CDC42 small effector 1
211302_s_at	34.25	12.62	2.71	+	PDE4B	phosphodiesterase 4B, cAMP-specific
215904_at	40.46	15.15	2.67	+	MLLT4	myeloid/lymphoid or mixed-lineage leukemia; translocated to, 4
205701_at	32.40	12.37	2.62	+	IPO8	importin 8
202388_at	61.10	23.45	2.61	-	RGS2	regulator of G-protein signalling 2, 24kDa

213446_s_at	17.87	6.86	2.60	+	IQGAP1	IQ motif containing GTPase activating protein 1
222201_s_at	23.74	9.21	2.58	-	CASP8AP2	CASP8 associated protein 2
201065_s_at	8.99	3.55	2.53	+	GTF2I	general transcription factor II, I
35150_at	7.62	3.06	2.49	+	CD40	CD40 antigen (TNF receptor superfamily member 5)
212294_at	10.32	4.16	2.48	-	GNG12	guanine nucleotide binding protein (G protein), gamma 12
200644_at	9.85	4.00	2.46	+	MARCKSL1	MARCKS-like 1
210221_at	14.37	5.85	2.46	+	CHRNA3	cholinergic receptor, nicotinic, alpha polypeptide 3
211245_x_at	28.38	11.62	2.44	+	KIR2DL4	killer cell immunoglobulin-like receptor, two domains, long cytoplasmic tail, 4
211242_x_at	78.57	32.17	2.44	+	KIR2DL4	killer cell immunoglobulin-like receptor, two domains, long cytoplasmic tail, 4
221386_at	17.71	7.29	2.43	+	OR3A2	olfactory receptor, family 3, subfamily A, member 2
202149_at	17.62	7.38	2.39	-	NEDD9	neural precursor cell expressed, developmentally down-regulated 9
201008_s_at	50.83	21.32	2.38	+	TXNIP	thioredoxin interacting protein
202467_s_at	6.12	2.57	2.38	-	COPS2	COP9 constitutive photomorphogenic homolog subunit 2 (Arabidopsis)
204396_s_at	14.32	6.12	2.34	-	GRK5	G protein-coupled receptor kinase 5
396_f_at	9.39	4.05	2.32	+	EPOR	erythropoietin receptor
201488_x_at	2.09	0.91	2.31	+	KHDRBS1	KH domain containing, RNA binding, signal transduction associated 1
221745_at	17.06	7.42	2.30	+	WDR68	WD repeat domain 68
207667_s_at	15.45	6.73	2.30	+	MAP2K3	mitogen-activated protein kinase kinase 3
209505_at	73.82	32.44	2.28	-	NR2F1	Nuclear receptor subfamily 2, group F, member 1
213401_s_at	76.88	33.94	2.27	-	---	---
202091_at	16.37	7.23	2.26	+	ARL2BP	ADP-ribosylation factor-like 2 binding protein
201009_s_at	25.86	11.52	2.25	+	TXNIP	thioredoxin interacting protein
213270_at	5.27	2.36	2.24	+	MPP2	membrane protein, palmitoylated 2 (MAGUK p55 subfamily member 2)
209239_at	4.89	2.27	2.15	+	NFKB1	nuclear factor of kappa light polypeptide gene enhancer in B-cells 1 (p105)
211599_x_at	8.68	4.06	2.14	+	MET	met proto-oncogene (hepatocyte growth factor receptor)
205578_at	21.90	10.27	2.13	-	ROR2	receptor tyrosine kinase-like orphan receptor 2
205176_s_at	5.32	2.50	2.13	-	ITGB3BP	integrin beta 3 binding protein (beta3-endonexin)
206132_at	1.84	0.87	2.11	+	MCC	mutated in colorectal cancers
203218_at	22.38	10.69	2.09	-	MAPK9	mitogen-activated protein kinase 9
33814_at	10.79	5.17	2.09	+	PAK4	p21(CDKN1A)-activated kinase 4
203077_s_at	5.06	2.43	2.08	-	SMAD2	SMAD, mothers against DPP homolog 2 (Drosophila)
201431_s_at	9.40	4.52	2.08	-	DPYSL3	dihydropyrimidinase-like 3

221060_s_at	14.80	7.12	2.08	+	TLR4	toll-like receptor 4
204712_at	58.79	28.53	2.06	-	WIF1	WNT inhibitory factor 1
200923_at	21.83	10.68	2.04	+	LGALS3BP	lectin, galactoside-binding, soluble, 3 binding protein
204064_at	8.66	4.25	2.04	-	THOC1	THO complex 1
218158_s_at	8.68	4.29	2.02	-	APPL	adaptor protein containing pH domain, PTB domain and leucine zipper motif 1
204813_at	7.04	3.50	2.01	+	MAPK10	mitogen-activated protein kinase 10
208486_at	3.82	1.91	2.00	+	DRD5	dopamine receptor D5
**Cation transport**						
205802_at	76.09	17.70	4.30	-	TRPC1	transient receptor potential cation channel, subfamily C, member 1
203688_at	16.25	4.21	3.86	-	PKD2	polycystic kidney disease 2 (autosomal dominant)
205803_s_at	21.92	6.71	3.26	-	TRPC1	transient receptor potential cation channel, subfamily C, member 1
212297_at	4.78	1.92	2.49	-	ATP13A3	ATPase type 13A3
208349_at	5.70	2.33	2.45	+	TRPA1	transient receptor potential cation channel, subfamily A, member 1
**Calcium ion transport**						
205802_at	60.75	14.13	4.30	-	TRPC1	transient receptor potential cation channel, subfamily C, member 1
205803_s_at	17.50	5.36	3.26	-	TRPC1	transient receptor potential cation channel, subfamily C, member 1
219090_at	32.29	13.55	2.38	-	SLC24A3	solute carrier family 24 (sodium/potassium/calcium exchanger), member 3
**Protein modification**						
220483_s_at	131.49	33.34	3.94	+	RNF19	ring finger protein 19
205571_at	16.80	4.32	3.89	-	LIPT1	lipoyltransferase 1
208689_s_at	13.18	4.81	2.74	+	RPN2	ribophorin II
213704_at	12.56	5.11	2.46	-	RABGGTB	Rab geranylgeranyltransferase, beta subunit
**Intracellular signaling cascade**						
209648_x_at	35.05	8.74	4.01	-	SOCS5	suppressor of cytokine signaling 5
208127_s_at	21.05	5.61	3.75	-	SOCS5	suppressor of cytokine signaling 5
219165_at	14.50	4.12	3.52	-	PDLIM2	PDZ and LIM domain 2 (mystique)
212729_at	13.42	3.94	3.41	+	DLG3	discs, large homolog 3 (neuroendocrine-dlg, Drosophila)
221748_s_at	17.17	5.23	3.28	-	TNS1	tensin 1

215829_at	13.31	4.23	3.15	+	SHANK2	SH3 and multiple ankyrin repeat domains 2
209895_at	68.09	22.31	3.05	+	PTPN11	protein tyrosine phosphatase, non-receptor type 11
212801_at	5.40	1.77	3.04	+	CIT	citron (rho-interacting, serine/threonine kinase 21)
202226_s_at	55.90	18.78	2.98	+	CRK	v-crk sarcoma virus CT10 oncogene homolog (avian)
213337_s_at	11.05	3.83	2.88	+	SOCS1	suppressor of cytokine signaling 1
209684_at	5.91	2.06	2.87	-	RIN2	Ras and Rab interactor 2
207732_s_at	17.40	6.20	2.81	+	DLG3	discs, large homolog 3 (neuroendocrine-dlg, Drosophila)
203370_s_at	30.18	11.04	2.73	-	PDLIM7	PDZ and LIM domain 7 (enigma)
213545_x_at	12.62	4.65	2.71	-	SNX3	sorting nexin 3
205880_at	6.88	2.57	2.68	-	PRKD1	protein kinase D1
210648_x_at	10.35	3.91	2.65	-	SNX3	sorting nexin 3
202114_at	10.97	4.15	2.64	-	SNX2	sorting nexin 2
218705_s_at	22.90	8.73	2.62	-	SNX24	sorting nexing 24
220300_at	24.59	9.42	2.61	-	RGS3	regulator of G-protein signalling 3
205147_x_at	5.11	2.01	2.54	+	NCF4	neutrophil cytosolic factor 4, 40kDa
207782_s_at	25.02	9.94	2.52	+	PSEN1	presenilin 1
200604_s_at	23.18	9.21	2.52	+	PRKAR1A	protein kinase, cAMP-dependent, regulatory, type I, alpha
200067_x_at	7.46	3.22	2.32	-	SNX3	sorting nexin 3
207105_s_at	5.09	2.20	2.32	+	PIK3R2	phosphoinositide-3-kinase, regulatory subunit 2 (p85 beta)
205170_at	9.41	4.22	2.23	+	STAT2	signal transducer and activator of transcription 2, 113kDa
215411_s_at	23.50	10.69	2.20	-	TRAF3IP2	TRAF3 interacting protein 2
219457_s_at	15.25	7.45	2.05	-	RIN3	Ras and Rab interactor 3
221526_x_at	12.87	6.32	2.04	+	PARD3	par-3 partitioning defective 3 homolog (C. elegans)
209154_at	3.29	1.66	1.98	-	TAX1BP3	Tax1 binding protein 3
202987_at	19.16	9.79	1.96	-	TRAF3IP2	TRAF3 interacting protein 2
**mRNA processing**						
222040_at	36.12	11.14	3.24	-	HNRPA1	heterogeneous nuclear ribonucleoprotein A1
208765_s_at	21.68	6.81	3.18	+	HNRPR	heterogeneous nuclear ribonucleoprotein R
221919_at	28.33	9.18	3.09	-	---	---
205063_at	23.40	7.98	2.93	-	SIP1	survival of motor neuron protein interacting protein 1
201488_x_at	2.29	0.99	2.31	+	KHDRBS1	KH domain containing, RNA binding, signal transduction associated 1

201224_s_at	10.50	4.62	2.27	+	SRRM1	serine/arginine repetitive matrix 1
**RNA splicing**						
200686_s_at	20.70	5.73	3.61	-	SFRS11	splicing factor, arginine/serine-rich 11
203376_at	7.85	2.56	3.06	-	CDC40	cell division cycle 40 homolog (yeast)
209162_s_at	45.56	16.91	2.69	+	PRPF4	PRP4 pre-mRNA processing factor 4 homolog (yeast)
200685_at	17.66	7.35	2.40	-	SFRS11	splicing factor, arginine/serine-rich 11
201362_at	9.18	4.04	2.27	-	IVNS1ABP	influenza virus NS1A binding protein
202127_at	10.12	4.53	2.23	-	PRPF4B	PRP4 pre-mRNA processing factor 4 homolog B (yeast)
221546_at	31.65	14.76	2.14	+	PRPF18	PRP18 pre-mRNA processing factor 18 homolog (yeast)
214016_s_at	8.05	4.02	2.00	-	SFPQ	Splicing factor proline/glutamine-rich
**Endotosis**						
209839_at	37.68	6.99	5.39	-	DNM3	dynamin 3
209684_at	3.32	1.16	2.87	-	RIN2	Ras and Rab interactor 2
213545_x_at	7.08	2.61	2.71	-	SNX3	sorting nexin 3
210648_x_at	5.81	2.20	2.65	-	SNX3	sorting nexin 3
202114_at	6.16	2.33	2.64	-	SNX2	sorting nexin 2
200067_x_at	4.19	1.81	2.32	-	SNX3	sorting nexin 3
207287_at	7.81	3.74	2.09	-	FLJ14107	hypothetical protein FLJ14107
219457_s_at	8.56	4.18	2.05	-	RIN3	Ras and Rab interactor 3
**Regulation of transcription from PolII promoter**						
219778_at	58.94	14.41	4.09	-	ZFPM2	zinc finger protein, multitype 2
221773_at	13.43	3.93	3.42	-	ELK3	ELK3, ETS-domain protein (SRF accessory protein 2)
211251_x_at	11.18	3.69	3.03	+	NFYC	nuclear transcription factor Y, gamma
202724_s_at	9.60	3.34	2.88	-	FOXO1A	forkhead box O1A
212257_s_at	14.37	5.13	2.80	+	SMARCA2	SWI/SNF related, matrix associated, actin dependent regulator of chromatin, subfamily a, membe
202216_x_at	9.15	3.28	2.79	+	NFYC	nuclear transcription factor Y, gamma
204349_at	9.97	3.90	2.56	-	CRSP9	cofactor required for Sp1 transcriptional activation, subunit 9, 33kDa
200604_s_at	18.43	7.33	2.52	+	PRKAR1A	protein kinase, cAMP-dependent, regulatory, type I, alpha
206858_s_at	13.06	5.74	2.28	-	HOXC6	homeo box C6

205170_at	7.49	3.35	2.23	+	STAT2	signal transducer and activator of transcription 2, 113kDa
213891_s_at	11.07	4.97	2.23	-	TCF4	Transcription factor 4
201073_s_at	9.51	4.49	2.12	+	SMARCC1	SWI/SNF related, matrix associated, actin dependent regulator of chromatin, subfamily c, membe
213251_at	2.17	1.07	2.03	-	SMARCA5	SWI/SNF related, matrix associated, actin dependent regulator of chromatin, subfamily a, membe
209292_at	21.21	10.46	2.03	-	ID4	Inhibitor of DNA binding 4, dominant negative helix-loop-helix protein
209189_at	61.47	30.61	2.01	-	FOS	v-fos FBJ murine osteosarcoma viral oncogene homolog
202172_at	6.04	3.07	1.97	-	ZNF161	zinc finger protein 161
**Regulation of cell cycle**						
216061_x_at	7.05	2.09	3.38	-	PDGFB	platelet-derived growth factor beta polypeptide
209550_at	23.27	7.33	3.18	-	NDN	necdin homolog (mouse)
214683_s_at	30.04	9.83	3.05	-	CLK1	CDC-like kinase 1
211251_x_at	11.58	3.82	3.03	+	NFYC	nuclear transcription factor Y, gamma
202216_x_at	9.48	3.40	2.79	+	NFYC	nuclear transcription factor Y, gamma
205106_at	47.82	17.22	2.78	+	MTCP1	mature T-cell proliferation 1
219910_at	4.96	1.83	2.71	+	HYPE	Huntingtin interacting protein E
207239_s_at	17.48	7.09	2.47	+	PCTK1	PCTAIRE protein kinase 1
202149_at	15.25	6.39	2.39	-	NEDD9	neural precursor cell expressed, developmentally down-regulated 9
38707_r_at	1.72	0.80	2.16	+	E2F4	E2F transcription factor 4, p107/p130-binding
204566_at	6.86	3.21	2.14	-	PPM1D	protein phosphatase 1D magnesium-dependent, delta isoform
201700_at	5.14	2.44	2.11	+	CCND3	cyclin D3
200712_s_at	5.65	2.72	2.07	+	MAPRE1	microtubule-associated protein, RP/EB family, member 1
206272_at	3.58	1.78	2.02	-	SPHAR	S-phase response (cyclin-related)
208824_x_at	11.71	5.83	2.01	+	PCTK1	PCTAIRE protein kinase 1
2028_s_at	1.07	0.55	1.95	+	E2F1	E2F transcription factor 1
**Protein complex assembly**						
212511_at	7.99	2.34	3.41	-	PICALM	phosphatidylinositol binding clathrin assembly protein
216711_s_at	10.27	3.05	3.37	+	TAF1	TAF1 RNA polymerase II, TATA box binding protein (TBP)-associated factor
200771_at	9.13	3.21	2.84	-	LAMC1	laminin, gamma 1 (formerly LAMB2)
201624_at	11.70	4.68	2.50	-	DARS	aspartyl-tRNA synthetase
35150_at	5.91	2.37	2.49	+	CD40	CD40 antigen (TNF receptor superfamily member 5)

213480_at	2.70	1.11	2.44	-	VAMP4	vesicle-associated membrane protein 4
213270_at	4.09	1.83	2.24	+	MPP2	membrane protein, palmitoylated 2 (MAGUK p55 subfamily member 2)
208829_at	8.14	3.73	2.18	+	TAPBP	TAP binding protein (tapasin)
216125_s_at	13.70	6.39	2.15	+	RANBP9	RAN binding protein 9
212128_s_at	12.43	5.88	2.11	+	DAG1	dystroglycan 1 (dystrophin-associated glycoprotein 1)
200841_s_at	41.38	20.07	2.06	+	EPRS	glutamyl-prolyl-tRNA synthetase
221526_x_at	9.49	4.67	2.04	+	PARD3	par-3 partitioning defective 3 homolog (C. elegans)
**Protein biosynthesis**						
218830_at	23.85	6.25	3.82	-	RPL26L1	ribosomal protein L26-like 1
202247_s_at	24.00	6.89	3.48	+	MTA1	metastasis associated 1
214317_x_at	21.82	7.39	2.95	-	RPS9	Ribosomal protein S9
200026_at	5.33	1.91	2.78	-	RPL34	ribosomal protein L34
200963_x_at	4.64	1.76	2.63	-	RPL31	ribosomal protein L31
221693_s_at	25.44	9.85	2.58	+	MRPS18A	mitochondrial ribosomal protein S18A
219762_s_at	15.45	6.27	2.46	-	RPL36	ribosomal protein L36
221593_s_at	22.43	9.34	2.40	-	RPL31	ribosomal protein L31
200091_s_at	3.20	1.36	2.35	-	RPS25	ribosomal protein S25
208756_at	9.21	4.09	2.25	+	EIF3S2	eukaryotic translation initiation factor 3, subunit 2 beta, 36kDa
203781_at	9.61	4.31	2.23	-	MRPL33	mitochondrial ribosomal protein L33
202926_at	9.86	4.58	2.15	+	NAG	neuroblastoma-amplified protein
213687_s_at	6.78	3.19	2.13	-	RPL35A	ribosomal protein L35a
212450_at	11.03	5.32	2.07	-	KIAA0256	KIAA0256 gene product
214143_x_at	4.08	2.08	1.96	-	RPL24	ribosomal protein L24
**Cell cycle**						
216711_s_at	14.05	4.17	3.37	+	TAF1	TAF1 RNA polymerase II, TATA box binding protein (TBP)-associated factor
215747_s_at	17.66	5.57	3.17	+	RCC1	regulator of chromosome condensation 1
203531_at	4.39	1.56	2.81	-	CUL5	cullin 5
213743_at	11.99	4.29	2.79	-	CCNT2	cyclin T2
217301_x_at	21.86	8.16	2.68	+	RBBP4	retinoblastoma binding protein 4
202388_at	64.82	24.87	2.61	-	RGS2	regulator of G-protein signalling 2, 24kDa

209903_s_at	10.39	4.17	2.49	-	ATR	ataxia telangiectasia and Rad3 related
205245_at	8.76	3.79	2.32	+	PARD6A	par-6 partitioning defective 6 homolog alpha (C.elegans)
213151_s_at	2.56	1.13	2.27	-	38967	septin 7
212332_at	63.97	29.53	2.17	+	RBL2	retinoblastoma-like 2 (p130)
205895_s_at	6.88	3.26	2.11	+	NOLC1	nucleolar and coiled-body phosphoprotein 1
206967_at	19.89	9.81	2.03	+	CCNT1	cyclin T1
